# In Pursuit of Low Energy Phosphorescence: Late Metal Coordination Complexes of the Planar, π‐extended Bipyridyl Ligand 6,6′,7,7′‐Biphenanthridine

**DOI:** 10.1002/chem.202501802

**Published:** 2025-09-08

**Authors:** Dion B. Nemez, Robert J. Ortiz, Keighlynn A. Veilleux, J. A. Gareth Williams, David E. Herbert

**Affiliations:** ^1^ Department of Chemistry and the Manitoba Institute for Materials University of Manitoba Winnipeg Manitoba R3T 2N2 Canada; ^2^ Department of Chemistry Durham University Durham DH1 3LE UK

## Abstract

The coordination chemistry of the planar, doubly π‐extended bipyridine analog, 6,6′,7,7′‐biphenanthridine (*p*‐biphe), is presented. The phenanthridine units in *p*‐biphe are fused together at the 6‐ and 7‐ positions, and the resulting rigid ligand is compared with the more flexible parent “biphe” fused only at the 6‐positions. *p*‐Biphe is intensely fluorescent in solution with a much higher quantum yield, but, unlike biphe, at 77 K the fluorescence is not accompanied by any significant phosphorescence. Two four‐coordinate Cu(I) complexes and pseudo‐octahedral Ru(II) and Ir(III) complexes are described: [Cu(*p*‐biphe)_2_]^+^, [(P^P)Cu(*p*‐biphe)]^+^, [Ru(bpy)_2_(*p*‐biphe)]^2+^, and [Ir(ppy)_2_(*p*‐biphe)]^+^, isolated as PF_6_ salts (P^P  =  4,5‐*bis*(diphenylphosphino)‐9,9‐dimethylxanthene; bpy = 2,2′‐bipyridine; ppy = 2‐phenylpyridine). The complexes are strongly coloured due to intense low‐energy absorption to charge‐transfer excited states based on TDDFT calculations. The acceptor nature of the *p*‐biphe ligand is clear from multiple reduction events observed electrochemically. The Ir(III) complex displays remarkably low‐energy phosphorescence (λ_max_ = 812 nm) in solution at room temperature with a vibrational progression evident extending into the near IR, with no contamination from visible light emission. While the heteroleptic Cu(I) complex is phosphorescent at 77 K, no emission is detectable from [Cu(*p*‐biphe)_2_]^+^ or [Ru(bpy)_2_(*p*‐biphe)]^2+^, likely due to competitive nonradiative decay.

## Graphical abstract

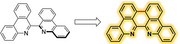

## 1 Introduction

**Figure 1:**
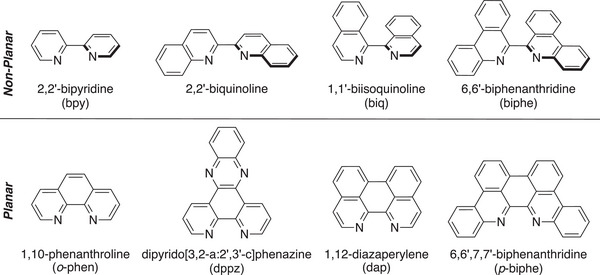
Nonplanar and planar benzannulated bipyridine proligands and their parents bpy and *o*‐phen.

Extending a ligand's π‐system through benzannulation of a conjugated manifold is a popular strategy for tuning the optical and electronic properties of both main group^[^ ^1^, ^2^, ^3^, ^4^, ^5^ ^]^ and transition metal^[^ ^6^, ^7^, ^8^, ^9^, ^10^, ^11^ ^]^ coordination complexes, especially those that can access charge‐transfer excited states involving the ligand. Benzannulation of bidentate ligands can take the form of extending isolated islands of ligand conjugation; compare, for example, the canonical chelating 2,2′‐bipyridine (bpy) with the ligands 2,2′‐biquinoline and 1,1′‐biisoquinoline (biq) (Figure 1).^[^ ^12^ ^]^ In these examples, the ligand's π‐system has been extended via benzannulation, but the two aromatic regions can still twist with respect to each other. This opens up the possibility of axial chirality as in 1,1′‐binaphthalene‐derived ligands such as BINOL or BINAP.^[^ ^13^ ^]^ Site‐selective benzannulation can also fuse together conjugated pyridinyl regions, forming formally planar ligand motifs. The simplest example of this, 1,10‐phenanthroline, is a classic ligand favoured for its versatile metal coordination with high stability constants. Phenanthroline complexes are often intensely luminescent and show proclivity for interacting with biomolecules such as DNA.^[^ ^14^ ^]^ While the extent of conjugation can vary depending on the precise structure,^[^ ^15^ ^]^ these applications commonly exploit both extended planar ligand structures and low‐lying, energetically accessible π*‐orbitals that are stabilized by π‐extension.^[^ ^16^ ^]^ Accordingly, significant efforts have been expended to grow the library of planar, π‐extended bipyridyl‐type ligands beyond the well‐known 1,12‐diazaperylene (dap)^[^ ^17^ ^]^ (Figure 1) and more nitrogenous analogs such as dipyrido[3,2‐a:2′,3′‐c]phenazine (dppz).^[^ ^18^ ^]^ This has led to a wealth of novel ligand classes including “heterosuperbenzenes,”^[^ ^19^ ^]^ helicene‐derived structures,^[^ ^20^ ^]^ “large‐surface area” ligands,^[^ ^21^ ^]^ and more.

**Scheme 1:**
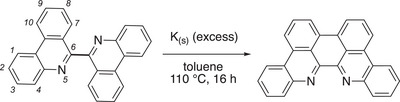
Synthesis of 6,6′,7,7′‐biphenanthridine (*p*‐biphe). Italicized numbers indicate the IUPAC numbering system for phenanthridines.

Our interest in benzannulated *N*‐heterocyclic donors based on phenanthridine (benzo[*c*]quinoline) ligands has ranged from bidentate *P^N* ^[^ ^22^ ^]^ and *C^N* ^[^ ^23^, ^24^ ^]^ donors to tridentate *N^C^N*,^[^ ^25^, ^26^ ^]^ *N^N^N* ^[^ ^27^ ^]^ and *N^N^O* ^[^ ^28^ ^]^ motifs. We recently reported the application of the bidentate 6,6′‐biphenanthridine (biphe, Figure 1), which combines the benzannulation seen in 2,2′‐biquinoline with that of 1,1′‐biisoquinoline, in the synthesis of deep‐red emitting homoleptic and heteroleptic Ru(II) coordination complexes.^[^ ^29^ ^]^ In those complexes, the phenanthridine sub‐units of the biphe ligand are not coplanar thanks to the transannular steric clash of the protons in the 7‐position of each tricyclic ring system (see Scheme 1 for IUPAC phenanthridine numbering system). This introduced a high level of malalignment in the Ru–N_biphe_ bond, increasing ligand photolability and rendering the complexes less suitable for photocatalytic applications. We therefore set out to investigate the consequences of planarizing 6,6′‐biphenanthridine through oxidative coupling in the 7,7′‐ positions. Herein we describe a simple synthetic route to the planar 6,6′,7,7′‐biphenanthridine ligand – which we shall refer to as *p*‐biphe – and a series of coordination complexes featuring this ligand bound to the 1^st^, 2^nd^ and 3^rd^ row transition‐metal ions Cu(I), Ru(II), and Ir(III), respectively. These coordination complexes of *p*‐biphe are found to show broad and intense absorption across the visible range of the electromagnetic spectrum, and photoluminescence extending into the deep red / near‐infrared (NIR).

## 2 Results and Discussion

While this work was underway, access to *p*‐biphe was reported via a one‐pot, sequential Pd‐mediated coupling / carbonyl‐amine condensation using 1,8‐dibromophenanthrene‐9,10‐dione and *N*‐Boc‐protected 2‐aminophenylboronic acid pinacol ester.^[^ ^30^ ^]^ We considered that planar *p*‐biphe can be conceptually derived from biphe by oxidative coupling of the two [CH] units in the 7 and 7′ positions, similar to the construction of dap from biq (Figure 1)^[^ ^17^ ^]^ via potassium‐induced aryl radical deprotonation followed by radical‐radical coupling to form the new C─C bond. Gratifyingly, stirring 6,6′‐biphenanthridine over excess potassium at elevated temperature produced the target molecule with high conversion and good isolated yields (71%).

**Figure 2:**
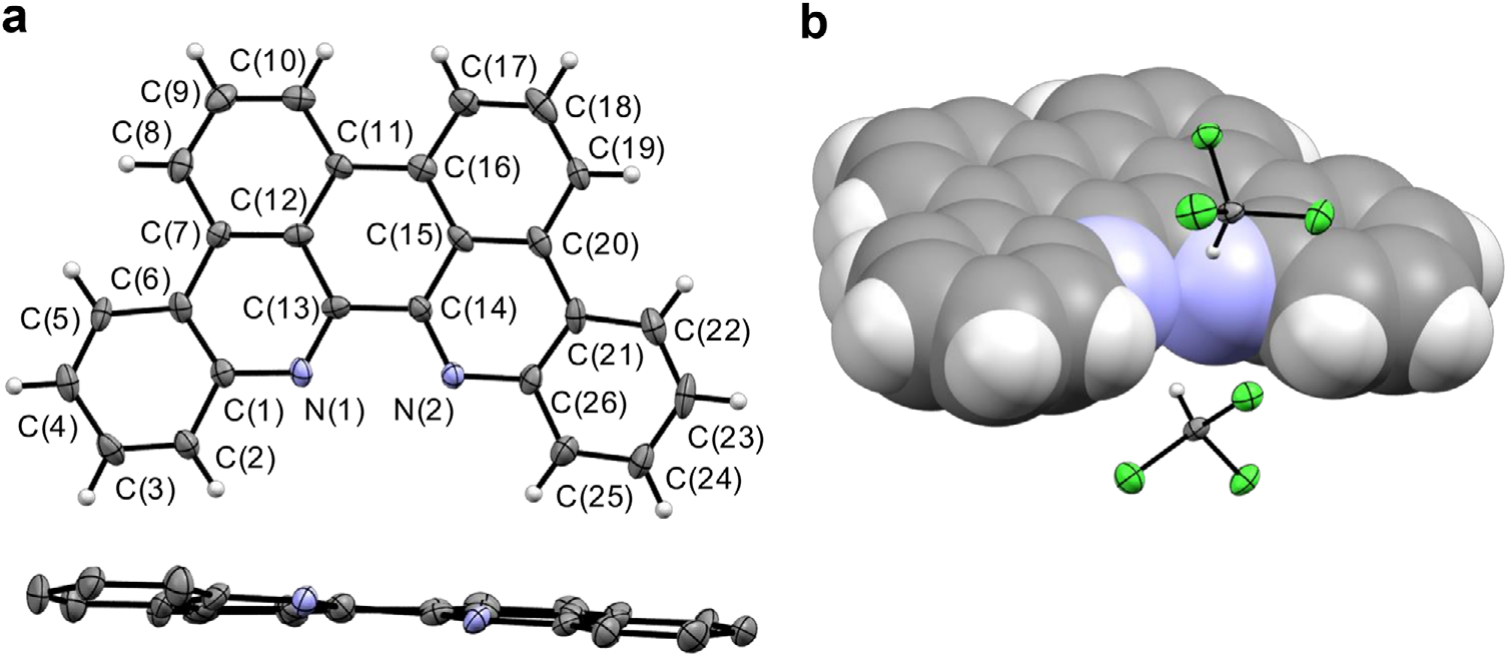
Solid‐state structure of *p*‐biphe (a) with ellipsoids shown at 50% probability and two co‐crystallized chloroform solvent molecules removed for clarity; (b) as a space‐filling model with solvent molecules included. Selected bond lengths (Å) and angles (°): C(13)‐C(14) 1.490(10), C(13)‐N(1) 1.308(9), C(14)‐N(2) 1.312(9), C(11)‐C(16) 1.484(10), C(6)‐C(7) 1.458(10), C(20)‐C(21) 1.450(11); N(1)‐C(13)‐C(14) 117.2(6), N(2)‐C(14)‐C(13) 117.2(6), N(1)‐C(1)‐C(2) 117.1(7), N(2)‐C(26)‐C(25) 117.2(7), N(1)‐C(13)‐C(14)‐N(2) 5.2(9), C(12)‐C(13)‐C(14)‐C(15) 6.5(9).

Somewhat surprisingly given the potential for extensive intermolecular π‐stacking interactions, *p*‐biphe is markedly more soluble than its nonplanar precursor, biphe. As expected, seven unique magnetic environments are observed by ^1^H NMR spectroscopy, with four doublets and three pseudo‐triplets in the aromatic region. The ^13^C{^1^H} NMR spectrum likewise shows the chemical environments of one half of the symmetrically equivalent molecule with thirteen unique signals. Interestingly, the ^13^C of the imine‐like [C═N] unit (6,6′ position) undergoes a significant upfield shift from 158.9 ppm to 149.3 ppm when compared to biphe. Single crystals suitable for X‐ray diffraction analysis were grown from chloroform solution (Figure 2). The analysis reveals the expected planar, fused structure comprising seven six‐membered rings, with the two constituent phenanthridine sub‐units oriented at an angle of 6° with respect to each other. This interplanar angle is slightly larger than that of 4° observed in the ethanol solvate of *p*‐biphe reported by Xu et al.^[^ ^30^ ^]^

With 28 π‐electrons, *p*‐biphe cannot satisfy Hückel's criteria for aromaticity for each six‐membered annular sub‐unit. The length of the C─C bond connecting the 6 and 6′ positions is statistically indistinguishable from that of the nonplanar precursor [C(13)‐C(14) = 1.490(10) Å *cf*. 1.500(2) Å in 6,6′‐biphenanthridine^[^ ^29^ ^]^] or of DAP [1.478(2) Å],^[^ ^31^ ^]^ and only slightly longer than the corresponding bond in 4,5‐dimethyl‐1,10‐phenanthroline [1.448(2) Å].^[^ ^32^ ^]^ Prior computational analysis of *p*‐biphe (undertaken as part of an in silico investigation of the potential of planar biphenanthridine derivatives for singlet‐fission applications)^[^ ^33^ ^]^ revealed the presence of a nodal plane between the two phenanthridine units in the highest occupied molecular orbital (HOMO). The C─C bond connecting the 7‐ and 7′‐ positions, in comparison, is much longer in *p*‐biphe [1.484(10) Å] and DAP [1.471(2) Å] than the corresponding bond in 4,5‐dimethyl‐1,10‐phenanthroline [1.365(2) Å], highlighting the impact of benzannulation in negating the ‘ethene‐bridged bipyridyl’ resonance contribution to the molecular ground‐state.^[^ ^15^ ^]^

**Figure 3:**
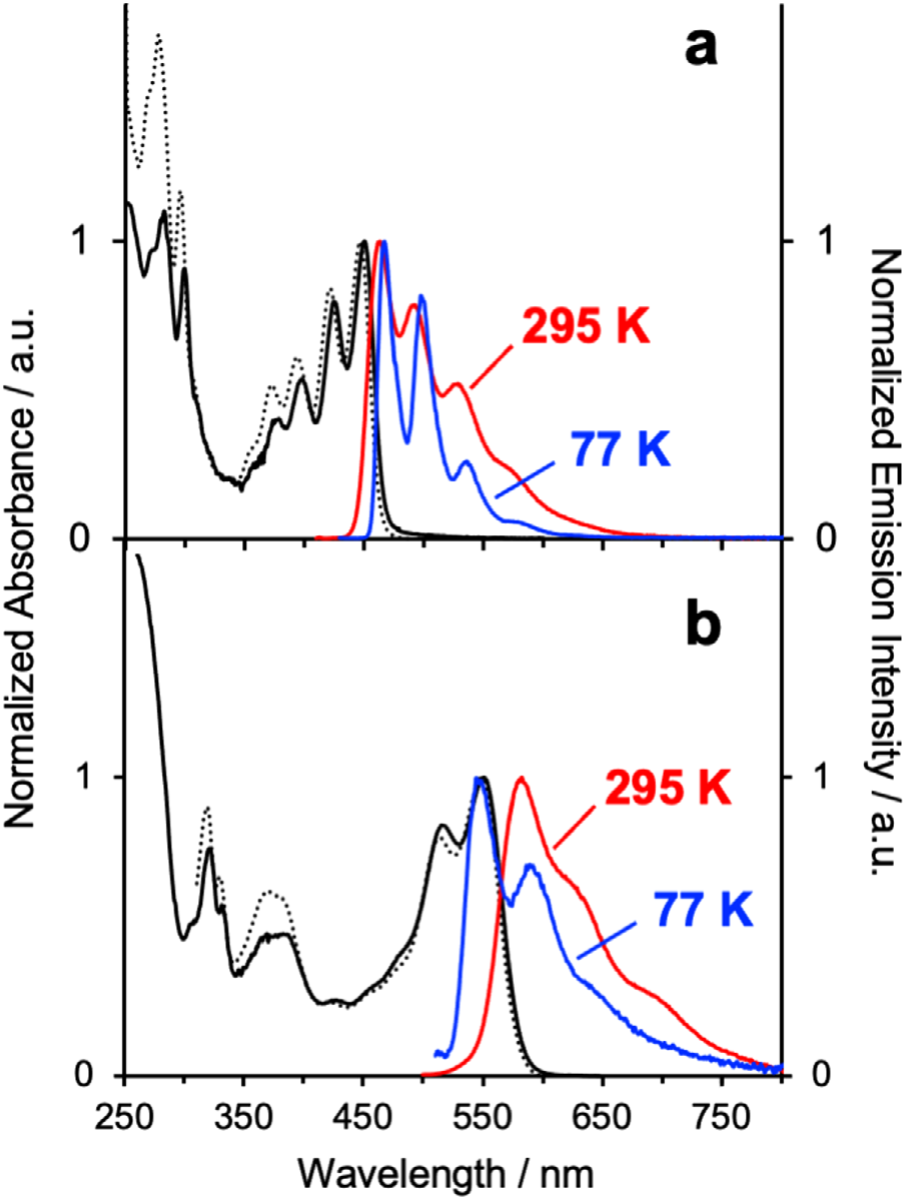
Overlays of the normalized absorption and excitation spectra (black solid and dotted lines respectively) with the emission spectra in CH_2_Cl_2_ at 295 K (red) and in EPA at 77 K (blue) for (a) *p*‐biphe and (b) *p*‐biphe in the presence of 1 equiv. of trifluoroacetic acid {EPA = diethyl ether / isopentane / ethanol (2:2:1 v/v)}.

The electronic absorption spectrum of *p*‐biphe (Figure 3a) is significantly different from that of its nonplanar precursor. While the UV‐Vis spectrum of biphe consists of a strong absorption at 252 nm accompanied by a series of somewhat sharper bands between 300–350 nm characteristic of phenanthridine derivatives,^[^ ^34^ ^]^ more intense and lower‐energy features are present in the spectrum of *p*‐biphe including prominent absorptions at 398, 426 and 451 nm with large molar absorptivity coefficients (5100–9500 M^−1^ cm^−1^; *cf*. λ_max_ = 350 nm, ε  =  4960 M^−1^ cm^−1^ for biphe^[^ ^29^ ^]^). The absorption spectrum is moderately solvatochromic, with the lowest energy absorption in particular exhibiting a bathochromic shift in solvents of increasing polarity (Figure S1). This trend tracks with the Lewis acidity of the solvents as determined by their acceptor number (Figure S2(a)),^[^ ^35^ ^]^ where increased Lewis acidity correlates with a red‐shifted absorption of the lowest energy transition, and also with Reichardt's E_T_(30) parameter (Figure S2(b)).^[^ ^36^ ^]^ We attribute this to stabilization of the LUMO due to solvent interactions in the binding pocket (Figure 2b; vide infra). *p*‐Biphe is intensely fluorescent in dichloromethane solution at room temperature (Figure 3a), red‐shifted compared to biphe ($\lambda_{\mathrm{em}}^{\max}$ = 464 versus 404 nm) and with a much higher quantum yield (Φ_lum_ = 0.41 versus 0.025). The spectrum shows clear vibrational structure, with a progression of around 1400 cm^−1^, while that of biphe does not under these conditions. The fluorescence lifetime is 2.2 ns, whereas that of biphe was too short‐lived to be determined using the available instrumentation.^[^ ^29^ ^]^ The enhanced emission and longer lifetime of *p*‐biphe doubtless reflect the higher rigidity associated with the fused structure, suppressing competitive nonradiative decay pathways. In a frozen glass at 77 K, the structure of the emission spectrum becomes more pronounced and the fluorescence lifetime increases to 5.0 ns, but no accompanying phosphorescence is observed at lower energy, even though the spectrum of biphe at 77 K shows both fluorescence and phosphorescence bands.^[^ ^29^ ^]^ Presumably, the more rigid, ring‐fused structure retards intersystem crossing to the triplet state so that triplet formation cannot compete effectively with fluorescence.

The addition of strong Brønsted acids such as trifluoromethane sulfonic acid or trifluoroacetic acid causes a dramatic bathochromic shift to both absorption and emission ($\lambda_{\mathrm{abs}}^{\max}$ = 549 nm, $\lambda_{\mathrm{em}}^{\max}$ = 583 nm; Figure 3b). We observe isosbestic points in the absorption spectrum at 384 and 456 nm, and at 556 nm in the emission spectrum, upon titrating acetonitrile solutions of *p*‐biphe with up to one equivalent of trifluoroacetic acid (Figure S3), indicating the formation of a single product. Contrary to prior reports,^[^ ^30^ ^]^ addition of further equivalents of acid did not yield any additional changes in the spectra, which we interpret as being consistent with mono‐protonation of the molecule. The luminescence quantum yield falls slightly for protonated *p*‐biphe to Φ_lum_ = 28% but still substantially exceeds that of protonated biphe (λ_max_ = 519 nm; Φ_lum_ = 0.10, τ = 2.4 ns) with a longer lifetime of 7.0 ns. Again, there is no evidence of any accompanying triplet phosphorescence at 77 K, whereas protonated biphe at low temperature shows both short‐lived fluorescence (τ = 8.4 ns) and lower‐energy phosphorescence bands (τ = 1.5 s).^[^ ^29^ ^]^

Previously conducted computational modelling of *p*‐biphe^[^ ^33^ ^]^ revealed that the highest occupied molecular orbital (HOMO) is a π‐bonding orbital with localized contribution from each C═N unit. The overall orbital density resembles two separate out‐of‐phase phenanthridine units, similar to biphe.^[^ ^29^ ^]^ In contrast to the nonplanarized analogue, however, the lowest unoccupied molecular orbital (LUMO) now spans across the N═C─C═N bridge (Figure S4). Monoprotonation breaks the molecular symmetry, resulting in unique orbital character in each phenanthridine half. This narrows the calculated HOMO‐LUMO gap of *p*‐biphe compared to biphe, consistent with the aforementioned red‐shifted absorption and emission. The stabilization of the emissive exited state is also reflected in the calculated energies of the first singlet excited state (S_1_) which for protonated *p*‐biphe lies 2.35 eV above the ground state compared to 2.88 eV for the neutral compound.

**Scheme 2:**
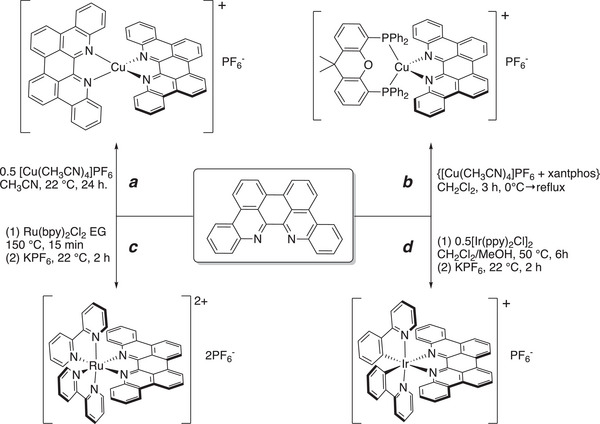
Preparation of (a) [Cu(*p*‐biphe)_2_]PF_6_, (b) [(P^P)Cu(*p*‐biphe)]PF_6_, (c) [Ru(bpy)_2_(*p*‐biphe)](PF_6_)_2_, and (d) [Ir(ppy)_2_(*p*‐biphe)]PF_6_.

*p*‐Biphe can also bind transition metal ions, where a bidentate coordination mode is expected. We prepared four representative coordination complexes for study: a homoleptic *bis*(*p*‐biphe) copper(I) hexafluorophosphate complex, [Cu(*p*‐biphe)_2_]PF_6_; a heteroleptic Cu(I) species [(P^P)Cu(*p*‐biphe)]PF_6_ (where P^P = 4,5‐*bis*(diphenylphosphino)‐9,9‐dimethylxanthene, a chelating bis‐phosphine commonly known as xantphos); a heteroleptic Ru(II) complex with bipyridine ligands [Ru(bpy)_2_(*p*‐biphe)](PF_6_)_2_; and a related heteroleptic Ir(III) complex with cyclometallated phenylpyridine (ppy) ligands [Ir(ppy)_2_(*p*‐biphe)]PF_6_ (Scheme 2). The homoleptic Cu(I) complex was prepared via reaction of two equivalents of *p*‐biphe with [Cu(CH_3_CN)_4_]PF_6_ in acetonitrile at room temperature and isolated as dark green crystals in 83% yield. The heteroleptic [(P^P)Cu(I)(*p*‐biphe)]PF_6_ was obtained in similar yield by first mixing the same Cu(I) reagent with xantphos in an ice‐cooled flask, followed by addition of *p*‐biphe and heating to reflux. The Ru(II) complex was synthesized by reaction of *p*‐biphe with *cis*‐Ru(bpy)_2_Cl_2_ in ethylene glycol at elevated temperature in a microwave reactor, and isolated as dark purple crystals in 66% yield. The cyclometallated Ir(III) complex was prepared from [Ir(ppy)_2_(*μ*‐Cl)]_2_ at a more modest temperature of 50 °C and isolated in similar yield (65%) as dark near‐black blocks. All species were diamagnetic and were characterized in solution by ^1^H and ^13^C NMR spectroscopy.

**Figure 4:**
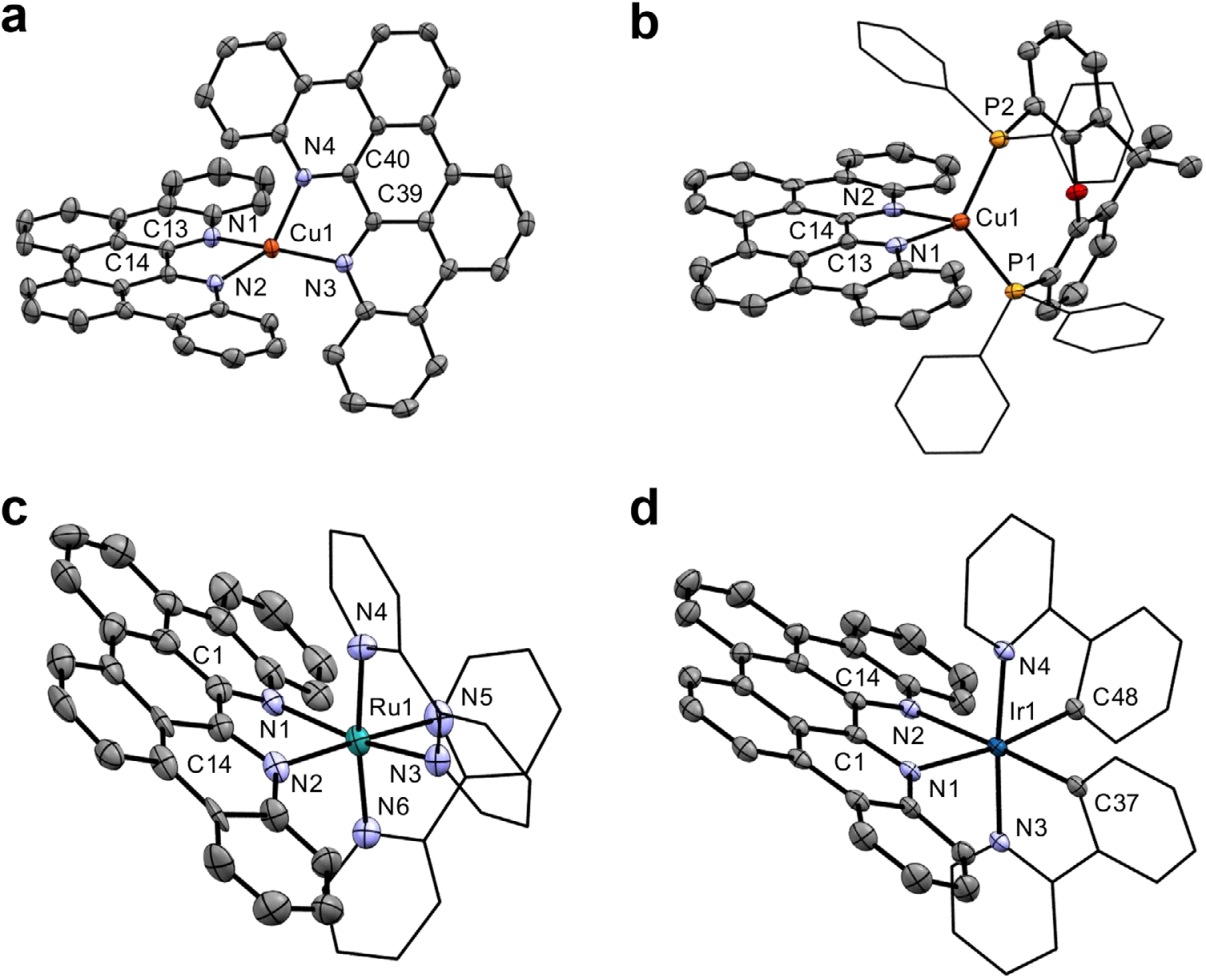
Solid‐state structures of (a) [Cu(*p*‐biphe)_2_]PF_6_, (b) [(P^P)Cu(*p*‐biphe)]PF_6_, (c) [Ru(bpy)_2_(*p*‐biphe)](PF_6_)_2_ and (d) [Ir(ppy)_2_(*p*‐biphe)]PF_6_ with thermal ellipsoids shown at 50% probability levels. Hydrogen atoms, counterions and co‐crystallized solvent molecules are omitted for clarity. Selected bond distances (Å) and angles (°): [Cu(*p*‐biphe)_2_]PF_6_ Cu(1)‐N(3) 1.9947(16), Cu(1)‐N(4) 2.0531(16), Cu(1)‐N(1) 2.0382(16), Cu(1)‐N(2) 1.9998(16); N(1)‐Cu(1)‐N(2) 81.12(6), N(3)‐Cu(1)‐N(4) 81.19(6). [(P^P)Cu(*p*‐biphe)]PF_6_ Cu(1)‐P(1) 2.2800(17), Cu(1)‐P(2) 2.2737(18), Cu(1)‐N(1) 2.070(5), Cu(1)‐N(2) 2.090(5); P(1)‐Cu1‐P(2) 118.51(7), N(1)‐Cu(1)‐N(2) 78.59(19). [Ru(bpy)_2_(*p*‐biphe)](PF_6_)_2_ Ru(1)‐N(1) 2.072(8), Ru(1)‐N(2) 2.085(8); N(1)‐Ru(1)‐N(3) 166.0(3), N(2)‐Ru(1)‐N(5) 177.0(3), N(4)‐Ru(1)‐N(6) 173.1(3). [Ir(ppy)_2_(*p*‐biphe)]PF_6_ Ir(1)‐N(1) 2.1801(15), Ir(1)‐N(2) 2.1853(17), Ir(1)‐C(37) 2.0119(19), Ir(1)‐C(48) 2.0208(18), Ir(1)‐N(3) 2.0382(16), Ir(1)‐N(4) 2.0668(16); N(3)‐Ir(1)‐N(4) 175.16(6), C(37)‐Ir(1)‐N(2) 177.25(7), C(48)‐Ir(1)‐N(1) 170.99(7).

The solid‐state structures of all four complexes were determined by single‐crystal X‐ray diffraction (Figure 4). A crystal structure of the trifluoromethanesulfonate salt of *bis*(*p*‐biphe)copper(I) was reported by Xu et al.^[^ ^30^ ^]^ but without accompanying yields or spectroscopic data beyond an absorption spectrum. The two structures are very similar (Figure S5), with *p‐*biphe bite angles close to 81°, and an average interligand N‐Cu‐N angle of 124.2° versus 125°. The bond lengths are also extremely similar, sporting an average Cu─N bond length of 2.022 Å compared with 2.018 Å. With a bite angle of 81°, the geometry about Cu(I) in the homoleptic complex sits exactly on the border between distorted tetrahedral and distorted sawhorse, as quantified by a τ_δ_ parameter of 0.63, while the previously reported structure sits a little closer to tetrahedral with a τ_δ_ parameter of 0.71.^[^ ^37^ ^]^ The approximate planarity of the *p*‐biphe is retained (interplanar angle between phenanthridine fragments of 5.1/7.6°). The two *p*‐biphe ligands do not sit orthogonal to one another; instead, the interplanar angle is 73.6°. When paired with the xanthphos ligand with its wider bite angle, [P(1)‐Cu1‐P(2) 118.51(7)°], the Cu(I) is more rigidly tetrahedral (τ_δ_ = 0.87). In response, the two phenanthridine units of the *p*‐biphe ligand are canted slightly relative to one another, with a slightly larger interplanar angle (11.7°). With Cu─N distances of 1.995(2) and 2.090(5) Å, the metal‐ligand interactions are similar to Cu(I) complexes of sterically encumbered 1,10‐phenanthrolines such as the 2,9‐dimethyl derivative [*d*(Cu‐N) = 2.019(2) – 2.047(2) Å].^[^ ^38^ ^]^ The σ‐bonding overlap between N and Cu is not overly distorted, with roughly linear (∼170°) angles formed by the metal centre, the ligating N atom and the centroids of the C_5_N ring of the phenanthridine sub‐units.

The consequence of planarization of *p*‐biphe is also stark in the pseudo‐octahedral Ru(II) and Ir(III) complexes, [Ru(bpy)_2_(*p*‐biphe)]^2+^ and [Ir(ppy)_2_(*p*‐biphe)]^+^. Compared to [Ru(bpy)_2_(biphe)]^2+^,^[^ ^29^ ^]^ the *p*‐biphe is approximately planar (inter‐phenanthridine angle of 9.7° *cf*. 63° for biphe) thanks to relief of transannular steric interaction through removal of the protons in the 7‐position of each tricyclic ring system. In the biphe complex, the pronounced dihedral angle led to the two phenanthridine units sitting on opposite sides of the equatorial plane of the pseudo‐octahedral Ru(II) center. In the *p*‐biphe complex, the planar ligand is forced to occupy only one side of the equatorial plane. The least‐squares plane occupied by the *p*‐biphe ligand sits at an angle of ∼35° to that formed by the equatorial donor atoms [N(1), N(2), N(3), N(4) and Ru(1)]. For the related Ir(III) complex, this angle is relaxed somewhat (∼26°) and the *p*‐biphe is more aligned with the equatorial plane of the heavier metal ion. Planarization of 6,6′,7,7′‐biphenanthridine in forming *p*‐biphe also impacts the octahedricity of these two complexes: the octahedricity parameter (Σ) calculated using the Octadist program^[^ ^39^ ^]^ for [Ru(bpy)_2_(*p*‐biphe)]^2+^ (92.0) is more in line with [Ru(bpy)_2_(biq)]^2+^ (80.3)^[^ ^40^ ^]^ than [Ru(bpy)_2_(biphe)]^2+^ (119.6),^[^ ^29^ ^]^ indicative of a less distorted structure. The corresponding value for [Ir(ppy)_2_(*p*‐biphe)]^+^ is 99.1 (Figure S6, Table S1). Remarkably, the distortions in both pseudo‐octahedral geometries appear to originate in how (mis‑)aligned the *p*‐biphe donor lone pairs are with the metal's equatorial plane. Space‐filling diagrams (Figure S7) appear to suggest room for relaxation of this parameter, which implies an electronic reason for this distortion (vide infra).

**Figure 5:**
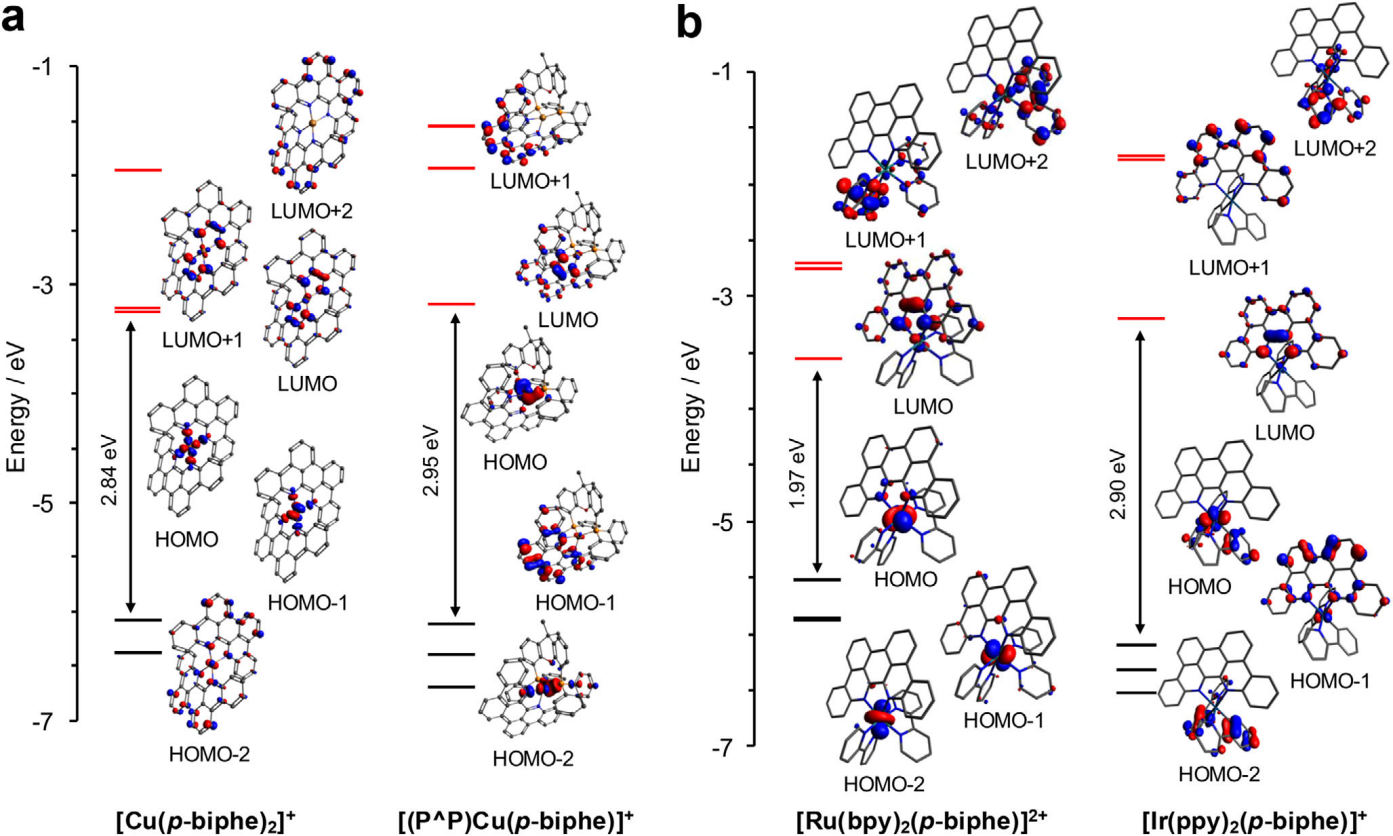
Molecular orbital energy level diagrams for (a) the four‐coordinate Cu(I) complexes and (b) the six‐coordinate Ru(II) and Ir(III) species. Isosurfaces of selected MOs are shown at isosurface values of 0.05.

The misalignment of nitrogen σ‐donor orbitals with the σ‐acceptor orbitals on the metal such as seen in [Ru(bpy)_2_(*p*‐biphe)]^2+^ and [Ir(ppy)_2_(*p*‐biphe)]^+^ is a common feature of complexes of atropisomeric ligands such as 1,1′‐biisoquinoline.^[^ ^41^ ^]^ Molecular orbitals (MOs) generated from the ground‐state equilibrium geometries of the cationic portions of the four coordination complexes were used to assess the bonding between the nitrogen donors of the ligands and the various metal ions (Figure 5). As geometry and electronic structure are, of course, intimately intertwined, we discuss the two pseudo‐tetrahedral Cu(I) species first (Figure 5a), followed by their six‐coordinate, pseudo‐octahedral counterparts (Figure 5b). The distorted, flattened structure of the homoleptic [Cu(*p*‐biphe)_2_]^+^ cation predicted by its τ_δ_ angle breaks the degeneracy of the three highest‐energy occupied MOs (HOMO through HOMO‐2) which visually present *d*_xy_, *d*_yz_ and *d*_xz_‐type character, though the Cu participation in the HOMO‐2 quantified by the Hirshfeld atomic population method is limited (Table S2). The HOMO has the most rigorously metal‐ligand σ* anti‐bonding character, while the metal's contribution to the HOMO‐2 is substantially lower than to the HOMO and HOMO‐1 (Table S2). The LUMO and LUMO+1 are nearly degenerate and present considerable π*(C═N) character within each phenanthridine moiety and also show π(C═C) bonding character to the C─C unit joining these two fragments at the 6,6′‐positions. For the heteroleptic Cu(I) cation in [(P^P)Cu(*p*‐biphe)]^+^, the HOMO has additional contributions from the xantphos phosphine donors. The LUMO has similar character to that in [Cu(*p*‐biphe)_2_]^+^ (Table S3). The three highest‐energy occupied MOs in the pseudo‐octahedral Ru(II) dication each have sizeable metal character and resemble what would be the *t*_2g_ orbitals in an octahedral complex (Table S4). For the Ir(III) monocation, the HOMO and HOMO‐2 both have significant 2‐phenylpyridyl character, with extensive metal participation only obvious in the HOMO and HOMO‐3 (Table S5). In comparision, in Ir(ppy)_3_, each of the HOMO, HOMO‐1 and HOMO‐2 show substantial metal‐ligand mixing with metal 5d character ranging from 44 to 52%.^[^ ^42^ ^]^ For [Ir(ppy)_2_(*p*‐biphe)]^+^, the HOMO‐1 has considerable orbital density localized on the planar 6,6′,7,7′‐biphenanthridine ligand. In fact, three of the four complexes presented here show substantial 6,6′,7,7′‐biphenanthridine ligand participation in the filled orbital manifolds: for [Cu(*p*‐biphe)_2_]^+^, the HOMO‐2 (95%); for [(P^P)Cu(*p*‐biphe)]^+^, the HOMO‐1 (96%); for [Ir(ppy)_2_(*p*‐biphe)]^+^, the HOMO‐1 (87%). The LUMOs of both [Ru(bpy)_2_(*p*‐biphe)]^2+^ and [Ir(ppy)_2_(*p*‐biphe)]^+^ both show the extensive phenanthridine π*(C = N) character expected of this ligand class.

**Figure 6:**
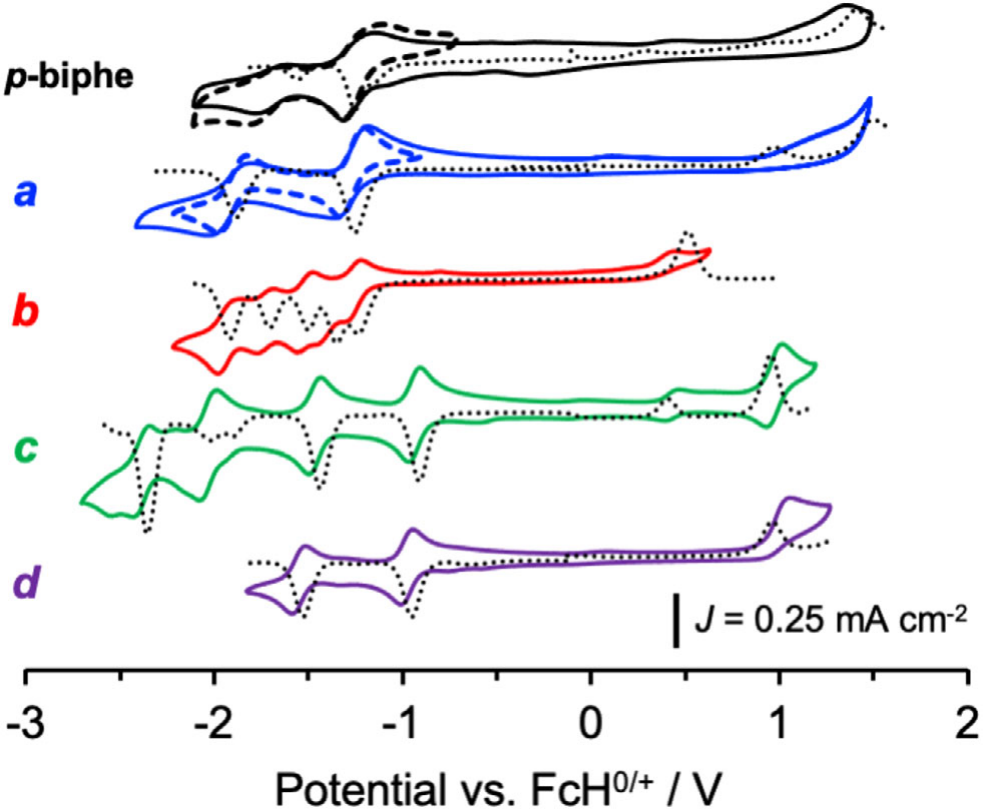
Cyclic voltammograms (scan rate = 100 mV s^−1^; solid lines) and differential pulse voltammograms (dotted lines) of *p*‐biphe (black lines, top) and (a) [(P^P)Cu(*p*‐biphe)]PF_6_ (blue), (b) [Cu(*p*‐biphe)_2_]PF_6_ (red), (c) [Ru(bpy)_2_(*p*‐biphe)](PF_6_)_2_ (green) and (d) [Ir(ppy)_2_(*p*‐biphe)]PF_6_ (purple); [analyte] = 0.3 mM, 0.1 M *n*Bu_4_NPF_6_, carbon disk working electrode, Pt wire counter electrode, Ag/Ag^+^ reference electrode; solvent = CH_3_CN for *p*‐biphe, *c* and *d*; CH_2_Cl_2_ for *a* and *b*.

The accessibility of the LUMO of 6,6′,7,7′‐biphenanthridine itself is highlighted by the observation of a quasi‐reversible reduction at ‐1.23 V versus ferrocene/ferrocenium (FcH^0/+^; Figure 6) in the cyclic voltammetry (CV). A second reduction is accessible at ‐1.54 V, but it shows a much lower degree of electrochemical reversibility. A similar reduction is accessible for the tetrahedral Cu(I) complexes [Cu(*p*‐biphe)_2_]PF_6_ (−1.26 V) and [(P^P)Cu(*p*‐biphe)]PF_6_ (−1.23 V). Neither 6,6′,7,7′‐biphenanthridine nor the two Cu(I) species showed reversible oxidation events. The homoleptic Cu(I) complex shows five reduction events in the CV (−1.23, −1.35, −1.50, −1.69, and‑1.92 V versus FcH^0/+^) which are well‐resolved by differential pulse voltammetry (DPV). All five one‐electron redox processes show a high level of reversibility. The Ru(II) complex [Ru(bpy)_2_(*p*‐biphe)](PF_6_)_2_ also shows a remarkable series of five reduction events at ‑0.91, −1.44, −1.90, −2.01 and −2.35 versus FcH^0/+^, the first three of which showed promising reversibility. These are anodically shifted compared to the analogous reductions of [Ru(bpy)_2_(biphe)]^2+^ (−1.18, −1.59, −2.02, −2.42 V versus FcH^0/+^), which are themselves at significantly less negative potentials than observed for [Ru(bpy)_3_]^2+^, [Ru(2,2′‐biquinoline)_3_]^2+^ or [Ru(3,3′‐biisoquinoline)_3_]^2+^, consistent with a more π‐extended scaffold.^[^ ^29^ ^]^ An accessible oxidation (+0.96 V versus FcH^0/+^) is also evident. The Ir(III) congener has two reversible reduction events (‐0.95, ‐1.53 V versus FcH^0/+^) and an irreversible oxidation at + 0.95 V versus FcH^0/+^. The observation of multiple, (quasi‐)reversible reductions is consistent with the energetic accessibility of the large, vacant π* system of the *p*‐biphe ligand. Molecular orbital modelling from single‐point calculations (Figure 5) predicts each metal complex should have multiple, low‐lying empty orbitals localized on different parts of the *p*‐biphe ligand. This suggests largely ligand‐centered character to these reductions.

**Figure 7:**
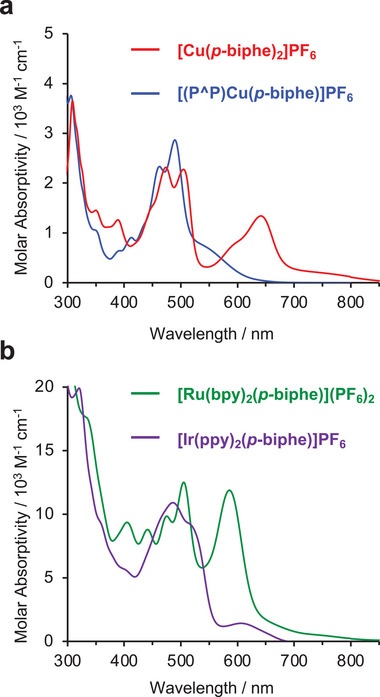
Electronic absorption spectra (295 K) of (a) [Cu(*p*‐biphe)_2_]PF_6_ and [(P^P)Cu(*p*‐biphe)]PF_6_ in CH_2_Cl_2_ and (b) [Ru(bpy)_2_(*p‐*biphe)](PF_6_)_2_ in CH_3_CN and [Ir(ppy)_2_(*p*‐biphe)]PF_6_ in CH_2_Cl_2_.

All four coordination complexes are strongly colored, influenced by the extended π‐system of *p*‐biphe. The colors of the Cu(I) complexes are impacted by the geometry and ligand identity. For example, the homoleptic species [Cu(*p*‐biphe)_2_]PF_6_ was isolated as dark green crystals, while the heteroleptic [(P^P)Cu(*p*‐biphe)]PF_6_ crystallized as a red‐brown solid. In solution, while the low‐energy tail of the absorption spectra of the heteroleptic species cuts off just past 600 nm, [Cu(*p*‐biphe)_2_]PF_6_ absorbs quite strongly to at least 800 nm with a discernible peak at 646 nm (ε = 1300 M^−1^ cm^−1^; Figure 7a and Table 1). Both complexes absorb to considerably longer wavelengths than the *p*‐biphe proligand on its own. Time‐dependent density functional theory (TDDFT) models the lowest energy absorption for [(P^P)Cu(*p*‐biphe)]^+^ to be dominated by the HOMO‐LUMO transition (Table S6). Inspecting the frontier orbital isosurfaces and the electron‐hole maps generated by TDDFT (Figure S10) enables assignment of this transition as metal‐to‐ligand charge transfer (MLCT), with the acceptor orbital localized on the *p*‐biphe ligand. In the homoleptic [Cu(*p*‐biphe)_2_]PF_6_, the red‐shifted lowest energy absorption is of mixed HOMO‐LUMO/(HOMO‐1)‐(LUMO+1) character (Table S7), which can be described as MLCT (Figure S11). The absorption spectrum of [Cu(*p*‐biphe)_2_]PF_6_ rivals the panchromatic absorption reported for heteroleptic nac‐nac Cu(I) bis‐chelates.^[^ ^43^ ^]^ Both Cu(I) *p*‐biphe complexes absorb to remarkably low energies compared to typical Cu(I) chromophores.^[^ ^44^ ^]^

**Table 1:** Photophysical properties of the four coordination complexes of *p*‐biphe.

	Absorption^[^ ^a^ ^]^ λ_max_ /nm (ε / M^−1^ cm^−1^)	Emission^[^ ^a^ ^]^ λ_max_ / nm	Φ_lum_^[^ ^a^ ^]^ / 10^−2^	τ^[^ ^a^ ^]^ /ns	Emission ^[^ ^b^ ^]^ 77 K	
					λ_max_/nm	τ/ns
[Cu(*p*‐biphe)_2_]PF_6_	505 (2180), 593sh, 646 (1300), 700–850sh	535 (^1^LC^[^ ^c^ ^]^)	0.54	9.3	465	4.8
[(P^P)Cu(*p*‐biphe)]PF_6_	305 (3640), 463 (2330), 490 (2850), 525–650sh	534 (^1^LC^[^ ^c^ ^]^)	0.08	9.3	465 (^1^LC) 734, 757, 818 (^3^LC/^3^MLCT)	4.8 (^1^LC) 40 × 10^3^ (^3^LC/^3^MLCT)
[Ru(bpy)_2_(*p*‐biphe)](PF_6_)_2_	483 (7950), 515 (11 690), 582 (11 800), 635–780sh	534 (^1^LC^[^ ^c^ ^]^)	0.02	7.9	466	7.9
[Ir(ppy)_2_(*p*‐biphe)]PF_6_	323 (19 920), 485 (10 910), 521sh, 609 (1420), 800 (220)	812 (^3^LC/^3^MLCT)	0.26	55	797	100

^[a]^ In CH_2_Cl_2_ at 295 K, except for the Ru(II) complex, for which the solvent was acetonitrile.

^[b]^ Cu(I) complexes in diethylether / isopentane / ethanol (2:2:1 v/v); Ru(II) and Ir(III) complexes in butyronitrile.

^[c]^ Note: the higher energy emission observed here using shorter wavelength excitation (λ_ex_ < 525 nm) is quite weak (Figure S15) precluding conclusive assignment of its origin.

The two pseudo‐octahedral complexes are likewise strongly colored (Figure 7b). The absorption spectrum of [Ru(bpy)_2_(*p‐*biphe)](PF_6_)_2_ is both bathochromically shifted and more intense than the biphe analogue [Ru(bpy)_2_(biphe)](PF_6_)_2_ [λ_max_ = 582 (ε = 11 800 M^−1^ cm^−1^), with a low energy shoulder tailing almost to 800 nm, versus λ_max_ = 530 nm (ε = 2300 M^−1^ cm^−1^)^[^ ^29^ ^]^] (Table 1). Thus, planarization of the biphenanthridine ligand leads to a stabilization of the MLCT manifold by nearly 1700 cm^−1^. This amounts to about half of the 3100 cm^−1^ stabilization that accompanies the replacement of bpy by (nonplanar) biphe in [Ru(bpy)_2_(biphe)]^2+^ compared to [Ru(bpy)_3_]^2+^.^[^ ^29^ ^]^ TDDFT calculations (Figure S12; Table S8) point toward the expected metal‐to‐ligand charge‐transfer (MLCT) character of the lowest energy absorption band, as the HOMO through HOMO‐2 all bear considerable metal ‘*t*_2g_’‐type character. The “acceptor” sites do differ: the LUMO, which is involved in the lowest energy calculated transitions, is localized on the *p‐*biphe (Figure 5), while the LUMO+1/LUMO+2 are bpy‐based (Table S4). The absorption spectrum of [Ir(ppy)_2_(*p*‐biphe)]PF_6_ is likewise starkly shifted to lower energy compared to, say, the archetypal [Ir(ppy)_2_(bpy)]^+^.^[^ ^45^ ^]^ Accordingly, *p*‐biphe dominates the acceptor orbital (LUMO) involved in the lowest energy transitions calculated by TDDFT (Table S9, Figure S13). While the HOMO has a large degree of metal participation (35%; Table S5), the HOMO‐2 bears considerable ppy character. This lends these lowest energy transitions mixed MLCT/LLCT character (ligand‐ligand charge transfer), typical of cyclometallated Ir(III) chromophores.^[^ ^46^ ^]^ There are also non‐negligible metal contributions to the LUMO through LUMO+2 ([Ru(bpy)_2_(*p*‐biphe)]^2+^, 4–8%; [Ir(ppy)_2_(*p*‐biphe)]^+^, 1–4%). Interestingly, concentrated solutions allow a weak but quite sharp absorption band to be detected at around 800 nm (ε ∼ 220 M^−1^ cm^−1^). Based on the position of the λ^0,0^ of the observed phosphorescence at only slightly longer wavelength than this (vide infra), we attribute this band to direct, formally forbidden S_0_→T_1_ excitation facilitated by the metal. While sometimes visible in Pt(II) complexes, it is unusual for absorption to the triplet state to be resolved from stronger overlapping singlet excitations in Ir(III) complexes.

**Figure 8:**
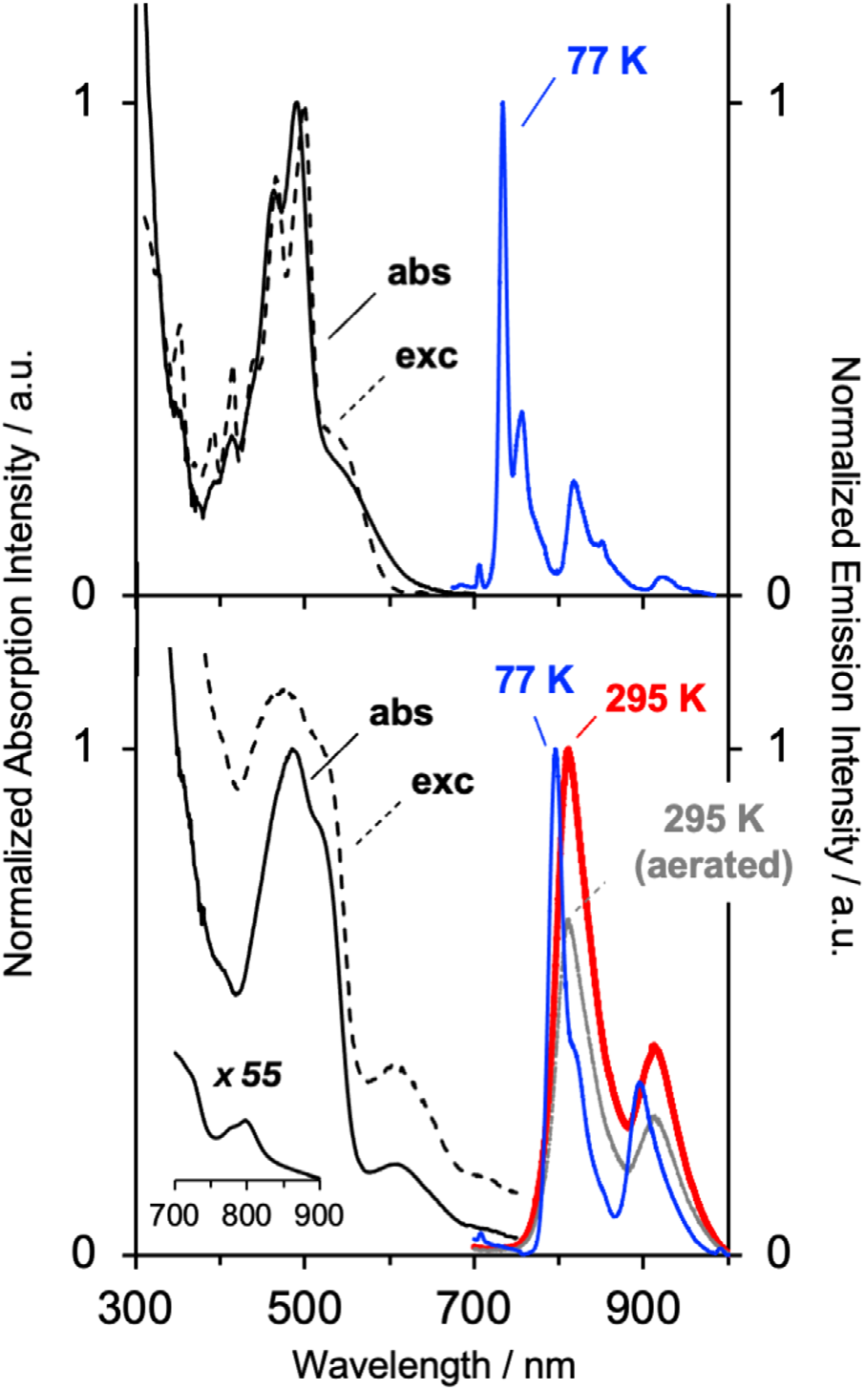
*Top*: Overlay of the absorption spectrum of [(P^P)Cu(*p*‐biphe)]PF_6_ in CH_2_Cl_2_ at 295 K (black line) with its excitation (λ_em_ = 755 nm, dashed black line) and emission (λ_ex_ = 550 nm) spectra in EPA at 77 K (blue line; EPA = diethylether / isopentane / ethanol; 2:2:1 v/v). *Bottom*: Overlay of the absorption spectrum of [Ir(ppy)_2_(*p*‐biphe)]PF_6_ (black line) with its excitation (λ_em_ = 755 nm, dashed black line) and emission spectra (λ_ex_ = 590 nm, red line for degassed, grey line for air‐equilibrated solutions, respectively) in CH_2_Cl_2_ at 295 K, and the emission spectrum in butyronitrile at 77 K (blue line). Inset: expansion showing a weak but definitive and sharp absorption band at 800 nm assigned to direct S_0_→T_1_ excitation.

Upon excitation into the lowest‐energy absorption band, only the Ir(III) complex displays detectable luminescence in deoxygenated solution at room temperature (Figure 8 and Table 1). The emission lies >800 nm, unusually long wavelengths for Ir(III)‐based emitters.^[^ ^47^ ^]^ Vibrational structure is well‐resolved, with λ_max_ = λ^0,0^ = 812 nm followed by a second main band at 913 nm, corresponding to a progression of around 1400 cm^−1^, typical of the C═C stretches of aromatic diamine ligands. This constitutes a challenging region of the spectrum for detectors. The spectrum shown in Figure 8 was recorded using a CCD detector, but its sensitivity — though superior to visible‐light photomultiplier tubes — falls off at λ > 950 nm. The use of a NIR PMT (sensitive to λ > 950 nm) shows that the emission tails far into the NIR to >1300 nm, with the 3^rd^ and even the 4^th^ components of the ∼1400 cm^−1^ vibrational progression resolved (Figure S14). The excitation spectrum closely matches the absorption spectrum (Figure 8). Although weak (Φ_lum_ = 0.26%), the deep‐red, solution‐phase phosphorescence is at remarkably low energy compared to state‐of‐the‐art, low‐energy emitting Ir(III) phosphors.^[^ ^48^, ^49^ ^]^ The limited examples of Ir(III) chromophores exhibiting emission wholly shifted above 700 nm present significant π‐extension, for example, via 1,4‐di(5‐*n*‐octylthiophen‐2‐yl)benzo[*g*]phthalazine substituents^[^ ^50^ ^]^ or multi‐nucleating ligand designs.^[^ ^51^ ^]^ The low quantum yield and relatively short phosphorescence lifetime of 55 ns indicate that nonradiative decay processes are highly competitive with emission from the triplet state, as is of course typical for low‐energy emitters (through the influence of the so‐called “energy gap law”). The emission is only quenched a little by oxygen in aerated solution (Figure 8), reflecting the rather short lifetime. At 77 K, the emission is similar to that at room temperature but slightly blue‐shifted, and the lifetime is increased to 100 ns.

The heteroleptic copper(I) complex [(P^P)Cu(*p*‐biphe)]PF_6_ also shows low‐energy emission upon excitation into its MLCT band – but only detectable at 77 K in this instance (Figure 8, Table 1). The emission is structured (λ_max_ = λ^0,0^ = 734 nm) and long‐lived (τ = 40 µs), suggesting that it originates from a largely ligand‐centered triplet state. The excitation spectrum tracks the room‐temperature absorption spectrum quite closely. The observation of structured low‐temperature emission can be put into the general context of heteroleptic phosphine / diimine Cu(I) complexes in the literature. For example, the 77 K emission spectrum of [(PPh_3_)_2_Cu(bpy)]^+^ shows a single broad peak at 610 nm, which shifts only slightly (to 612 nm) in the analogue with P^P‐chelating dppe.^[^ ^52^ ^]^ Meanwhile, the 77 K spectrum of [(PPh_3_)_2_Cu(*o*‐phen)]^+^ shows a long‐lived, structured component to the emission (τ = 115 µs) along with a broad band emitting on a much faster timescale (τ < 1 ns).^[^ ^53^ ^]^ It is unlikely that the low‐energy emission seen in our case for [(P^P)Cu(*p*‐biphe)]PF_6_ is due to phosphorescence from traces of dissociated free *p*‐biphe ligand, as *p*‐biphe itself showed no such emission (vide supra).

In contrast, no emission was detectable (even at 77 K) from the homoleptic Cu(I) complex or the Ru(II) complex upon excitation into their low‐energy MLCT absorption bands. Quite weak emission was detectable from these two complexes at room temperature when excited at higher energy (λ_ex_ < 525 nm; Figure S15). At 295 K, the emissive state generated by this higher‐energy excitation has a similar energy ($\lambda_{\mathrm{em}}^{\max}$ = 534 nm) and lifetime for [Cu(*p*‐biphe)_2_]PF_6_ (τ = 9.3 ns; Φ_lum_ = 0.0054) and for [Ru(bpy)_2_(*p*‐biphe)](PF_6_)_2_ (τ = 7.9 ns; Φ_lum_ = 0.0002) and, indeed, also for the heteroleptic [(P^P)Cu(*p*‐biphe)]PF_6_ (τ = 9.3 ns; Φ_lum_ = 0.0008) when excited at higher energy. In each case, the excitation spectra track the absorption spectra, apart from the lowest energy MLCT band. Collectively, these features might indicate spin‐conserved fluorescence from a *p*‐biphe‐centered excited state. We emphasize that this assignment is tentative; we cannot completely rule out the possibility of traces of the free or protonated ligand being responsible, though the emission maxima and lifetimes are inconsistent with such an explanation.

Spin‐density plots for the lowest‐energy optimized triplet states (T_1_) of all four complexes are rather similar, showing both metal and *p*‐biphe ligand involvement (Figures S16 and S17). Experimentally, the well‐resolved vibrational structure in the emission spectrum of [Ir(ppy)_2_(*p*‐biphe)]PF_6_ is consistent with predominant ligand‐centered (LC) character to the emissive triplet state. This is borne out by the Mulliken spin populations, which show minor (∼6%) Ir involvement at the calculated T_1_ geometry (Table S10). In all cases, ligand spin density is localized on the {N═C─C═N} bridge of the *p*–biphe as predicted by the LUMO isosurface. Interestingly, for the low‐energy emitter, [Ir(ppy)_2_(*p*‐biphe)]PF_6_, the spin density is more delocalized on the *p*‐biphe ligand when calculated at the optimized T_1_ geometry compared with at the ground‐state (S_0_) optimized geometry. Phosphorescence from T_1_ can be calculated for [Ir(ppy)_2_(*p*‐biphe)]^+^ at 1.25 eV or ∼990 nm (Table S11), giving reasonable agreement with the experimental emission maximum of 813 nm (∼1.5 eV). For [(P^P)Cu(*p*‐biphe)]PF_6_, the calculated energy difference between the T_1_ and S_0_ surfaces at the optimized triplet state geometry is 0.93 eV (1300 nm), as the T_1_ state is quite distorted compared to the ground‐state geometry (Table S12). However, the difference between the calculated T_1_ and S_0_ energies at the ground‐state geometry (i.e., using T_1_ at the ground‐state geometry as a stand‐in for a “frozen” structure) is 1.69 eV (734 nm), which agrees well with the experimentally observed emission at 77 K. Commonly, solution‐based emission is not observed at room temperature in copper complexes due to the potential for significant molecular distortion upon excitation, resulting in a nonradiative geometric “flattened state”,^[^ ^54^ ^]^ but upon cooling, emission may be turned on due to enhanced rigidity. In comparison, the calculated energy difference between the T_1_ and S_0_ states at the optimized triplet geometry of [Cu(*p*‐biphe)_2_]PF_6_ is far outside our window of detection (0.37 eV, 3351 nm; 1.14 eV, 1090 nm at the ground‐state geometry) and emission probably cannot compete with rapid nonradiative decay as per the ‘Energy Gap Law’.^[^ ^55^ ^]^ Optimization of the lowest‐lying triplet excited state of [Ru(bpy)_2_(*p*‐biphe)](PF_6_)_2_ also predicts considerable distortion (Table S13, Figure S18), leading to a very low energy T_1_ state (ΔE_T1‐S0_ = 1.08 eV, λ_em_ (calc) ∼1150 nm at the optimized T_1_ geometry). This predicted low‐energy emission is consistent with increased LUMO stabilization via planarization when comparing *p*‐biphe to biphe, as the analogous nonplanar complex [Ru(bpy)_2_(biphe)]^2+^ emits at 752 nm at room temperature.^[^ ^29^ ^]^ The lack of detectable emission from [Ru(bpy)_2_(*p*‐biphe)](PF_6_)_2_ compared to [Ir(ppy)_2_(*p*‐biphe)]PF_6_ likely results from smaller spin‐orbit coupling (SOC) for the 2^nd^ row metal, which is generally less effective at promoting radiative decay, conspiring with energetically accessible nonradiative pathways as predicted by the Energy Gap Law.

## 3 Conclusions

In conclusion, the rigidification achieved in *p*‐biphe compared to biphe – by fusing the 6‐ and the 7‐positions of the two constituent phenanthridine rings – is found to modify the properties of both the ligand and its complexes in significant ways. For instance, in the free ligand itself, the rigidification greatly enhances the fluorescence quantum yield in solution, by supressing nonradiative decay processes. Interestingly, at 77 K fluorescence is not accompanied by any significant phosphorescence, unlike for biphe. Meanwhile, in the complexes, it leads to lower‐energy excited states of charge‐transfer character. The acceptor nature of the *p*‐biphe ligand is also evident in the multiple, accessible reduction events observed electrochemically. Notably, the [Ir(ppy)_2_(*p*‐biphe)]^+^ complex is phosphorescent in solution at room temperature, with unusually narrow‐band emission deep into the near‐infrared (λ_max_ = 812 nm) with vibrational shoulders evident at wavelengths >1000 nm, and with no contamination from visible light emission. The heteroleptic Cu(I) complex [(P^P)Cu(*p*‐biphe)]PF_6_ is phosphorescent at 77 K at similarly long wavelengths (λ_emission_ = 734, 757, 818 nm). The structured nature of the emission spectrum is consistent with significant ligand‐localized character to the excited state, which is corroborated by spin‐density calculations. The NIR emission of [Ir(ppy)_2_(*p*‐biphe)]PF_6_, in particular, is quite remarkable;^[^ ^56^ ^]^ very few examples of Ir chromophores with comparable photophysical properties have been reported, and are limited to a binuclear motif^[^ ^51^ ^]^ or a homoleptic neutral species supported by 1,4‐di(thiophen‐2‐yl)benzo[*g*]phthalazine ligands.^[^ ^57^ ^]^ The presence of the large, planar ligand surface thus shifts absorption and emission further to very low energies, enhancing its intensity compared to complexes of nonplanar biphe. Efforts to exploit these favorable properties in applications including photocatalysis are currently underway.

## 4 Experimental Section

### 4.1 Materials and Methods

Air‐sensitive manipulations were carried out in either an N_2_‐filled glove box or using standard Schlenk techniques under Ar. 6,6′‐Biphenanthridine,^[^ ^29^ ^]^ Ru(bpy)_2_Cl_2_ (bpy = 2,2′‐bipyridine)^[^ ^11^ ^]^ and dichlorotetrakis(2‐(2‐pyridinyl)phenyl)diiridium(III)^[^ ^58^ ^]^ were prepared according to literature procedures. All other reagents were purchased from commercial suppliers and used without further purification. Organic solvents were dried and distilled using appropriate drying agents, and distilled water was degassed prior to use. 1‐ and 2D NMR spectra were recorded on a Bruker Avance 400 MHz or Bruker Avance – III 500 MHz spectrometer. ^1^H and ^13^C{^1^H} NMR spectra were referenced to residual solvent peaks. High resolution mass spectra (HRMS) were recorded using a Bruker microOTOF‐QIII. Cyclic voltammetry (CV) and differential pulse voltammetry (DPV) experiments were performed using a CHI 760c bipotentiostat, freshly polished (0.05 µm alumina paste) glassy carbon (GCE) disc working electrode, a Pt wire counter electrode, and a Ag/AgCl aqueous quasi‐reference electrode. Electrochemical grade *n*Bu_4_NPF_6_ was used as electrolyte (Sigma Aldrich, 0.1 M). Cyclic voltammetric (CV) experiments were conducted using scan rate of 100–300 mV/s. Differential pulse voltammetry (DPV) experiments were performed using a 5 mV increment, 50 mV amplitude, 0.1 s pulse width, 0.0167 s sample width, and 0.5 s pulse period. Ferrocene (FcH) was added as an internal standard to each solution upon completion of each set of cyclic voltammetry experiments, allowing potentials to be referenced to the ferrocene/ferrocenium (FcH^0/+^) redox couple.

#### 4.1.1 Synthesis of 6,6′,7,7′‐biphenanthridine (*p*‐biphe)

A 100 mL Teflon stoppered flask was charged with 6,6′‐biphenanthridine (0.306 g, 0.864 mmol) and dried in an oven at ∼100 °C for 2 d. Anhydrous toluene (40 mL) was then added, followed by metallic potassium (0.202 g, 5.17 mmol; 6 equiv.) under an atmosphere of Ar. This mixture was vigorously stirred in an oil bath set to 100 °C for 5 d, after which time the mixture had darkened to a near‐black. The mixture was neutralized via slow addition of methanol, followed by water, with stirring. After removing the methanol under reduced pressure, the resulting suspension was filtered, and the precipitate washed several times with ethanol (3×50 mL) then dried to leave a dark brown solid. Additional material was extracted from the water / ethanol filtrate with chloroform. The combined solids were then sonicated in ethanol (15 mL) and isolated via centrifugation/decanting. This was repeated three times. The product was then dried under vacuum to give a beige solid. Yield = 0.216 g (71%). ^1^H NMR (400 MHz, CDCl_3_, 22 °C): δ 8.67 (d, 2H, *J* = 8.2 Hz), 8.62 (t, 4H, *J* = 7.8 Hz), 8.52 (d, 2H, *J* = 7.6 Hz), 7.97 (t, 2H, *J* = 7.9 Hz), 7.84 (ddd, 2H, *J* = 8.3, 7.0, 1.4 Hz), 7.75 ppm (ddd, 2H, *J* = 8.2, 7.0, 1.4 Hz). ^1^H NMR (500 MHz, DMSO‐d_6_, 22 °C): δ 8.95 (d, 2H, *J* = 8.2 Hz), 8.88 (dd, 2H, *J* = 8.3, 1.4 Hz), 8.83 (d, 2H, *J* = 7.6 Hz), 8.40 (dd, 2H, *J* = 8.2, 1.3 Hz), 8.10 (t, 2H, *J* = 7.9 Hz), 7.92 (ddd, 2H, *J* = 8.1, 6.8, 1.3 Hz), 7.84 ppm (ddd, 2H, *J* = 8.2, 6.9, 1.4 Hz). ^13^C{^1^H} NMR (125 MHz, DMSO‐d_6_, 22 °C): δ 149.3, 144.6, 133.2, 131.6, 130.8, 130.6, 129.7, 128.3, 123.9, 123.5, 123.4, 123.1, 121.6 ppm.

#### 4.1.2 Synthesis of [Cu(*p*‐biphe)_2_]PF_6_

A 10 mL Teflon stoppered flask was charged with anhydrous acetonitrile (5 mL), *tetrakis*(acetonitrile)copper(I) hexafluorophosphate (0.025 g, 0.067 mmol, 1.0 equiv.) and *p*‐biphe (0.050 g, 0.141 mmol, 2.1 equiv.) and the mixture stirred at ambient temperature for 24 h. The resulting suspension was filtered, and the precipitate was washed with acetonitrile. The filtrate was collected and combined with the washings, and the solvent removed via reduced pressure. The resulting powder was then dissolved in degassed anhydrous dichloromethane and was recrystalized via layering with hexanes, giving dark green crystals. Yield = 0.058 g (94%). ^1^H NMR (500 MHz, CD_2_Cl_2_, 22 °C): δ 8.95 (d, 4H, *J* = 7.8 Hz, *H*_10_), 8.91 (d, 4H, *J* = 8.3 Hz, *H*_8_), 8.76 (d, 4H, *J* = 8.2 Hz, *H*_5_), 8.35 (t, 4H, *J* = 8.0 Hz, *H*_9_), 7.98 (d, 4H, *J* = 8.2 Hz, *H*_2_), 7.70 (t, 4H, *J* = 8.2 Hz, *H*_4_), 7.33 ppm (t, 4H, *J* = 8.2 Hz, *H*_3_). ^13^C{^1^H} NMR (125 MHz, CD_2_Cl_2_, 22 °C): δ 148.2, 142.2, 133.9, 133.0 (*C*_9_), 131.0, 130.4 (*C*_3_), 129.8 (*C*_4_), 128.9, 128.5 (*C*_2_), 128.1, 126.9, 125.2, 124.4 (*C*_10_), 123.4 (*C*_8_), 123.3 (*C*_5_), 122.1 ppm. HR‐MS (ESI‐TOF/MS, m/z) calcd. for C_52_H_28_CuN_4_ [M]^+^, 771.1604; found 771.1663. UV‐Vis (CH_2_Cl_2_, 22 °C): 646 nm (MLCT; 1300 M^−1^ cm^−1^).


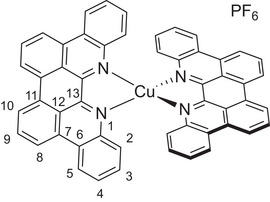


#### 4.1.3 Synthesis of [(P^P)Cu(*p*‐biphe)]PF_6_

A 10 mL Teflon stoppered flask was charged with anhydrous dichloromethane (5 mL), *tetrakis*(acetonitrile)copper(I) hexafluorophosphate (0.026 g, 0.070 mmol, 1.0 equiv.) and xantphos (0.041 g, 0.070 mmol, 1.0 equiv.) and the resulting mixture stirred under reflux for 16 h. The reaction mixture was then cooled to 0 °C using an ice bath. Under an Ar atmosphere, *p*‐biphe (0.026 g, 0.073 mmol, 1.05 equiv.) was added and the mixture heated at reflux for an additional 3 h. The reaction was then left to cool and the product precipitate by addition of hexanes (5 mL). Recrystallization from dichloromethane and hexanes gave red‐brown crystals. Yield = 0.066 g (83%). ^1^H NMR (500 MHz, CD_2_Cl_2_, 22 °C): δ 8.75–8.76 (d, 2H, *J* = 8.3 Hz, *H*_27_), 8.74–8.75 (d, 2H, *J* = 7.7 Hz, *H*_29_), 8.64 (d, 2H, *J* = 8.3 Hz, *H*_24_), 8.21 (t, 2H, *J* = 8.0 Hz, *H*_28_), 8.12–8.14 (d, 2H, *J* = 8.3 Hz, *H*_21_), 7.78–7.80 (d, 2H, *J* = 8.0 Hz, *H_Ar_*), 7.66–7.69 (t, 2H, *J* = 7.7 Hz, *H*_23_), 7.15–7.19 (m, 6H, *H_Ar_*), 7.07 (t, 2H, *J* = 7.7 Hz, *H*_22_), 6.95–7.03 (m, 16H, *H_Ar_*), 6.77–6.81 (m, 2H, *H_Ar_*), 1.91 ppm (s, 6H, *H*_1_). ^13^C{^1^H} NMR (125 MHz, CD_2_Cl_2_, 22 °C): δ 155.4 (*C*_32_), 142.5, 134.4, 133.9, 133.4, 133.3 (*C*_28_), 131.2, 130.9 (*C*_21_), 130.5, 130.0 (*C*_23_), 129.6 (*C*_22_), 129.0, 128.4, 128.2, 128.1, 128.0, 126.1, 125.9, 124.4 (*C*_29_), 123.5 (*C*_27_), 123.4 (*C*_24_), 121.9, 120.7, 35.0 (*C*_2_), 28.8 (*C*_1_) ppm. HR‐MS (ESI‐TOF/MS, m/z) calcd. for C_65_H_46_CuN_2_OP_2_ [M]^+^, 995.2376; found 995.2446. UV‐Vis (CH_2_Cl_2_, 22 °C): 553(sh) nm (MLCT; 750 M^−1^ cm^−1^).


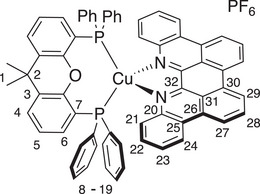


#### 4.1.4 Synthesis of [Ru(bpy)_2_(*p*‐biphe)](PF_6_)_2_

A glass vessel was charged with *p*‐biphe (0.061 g, 0.17 mmol, 1.0 equiv.), *bis*(2,2′‐bipyridine)(*cis*‐dichloro)ruthenium dihydrate (0.089 g, 0.17 mmol, 1.0 equiv.) and a stir bar. Ethylene glycol (3 mL) was added and the suspension was sparged with Ar for 15 min then sealed with a gas‐tight gap. The suspension was heated with stirring in an Anton‐Parr Monowave reactor to 150 °C for 15 min, then cooled to room temperature. An aqueous solution of potassium hexafluorophosphate was added (0.25 M, 2 mL), and, after 4 h of stirring, the resulting suspension was filtered over Celite and washed with water (5 mL) and diethylether (5 mL). The solid product was extracted off the Celite pad using acetonitrile. The crude product was recrystalized by layering an acetonitrile with diethylether, giving dark purple crystals. Yield = 0.120 g (66%). ^1^H NMR (400 MHz, CD_3_CN, 22 °C): δ 8.88 (dd, 4H, *J* = 11.67, 8.02 Hz, *H*_8,10_), 8.78 (d, 2H, *J* = 8.78 Hz, *H*_5_), 8.40–8.44 (m, 4H, *H*_17,20_), 8.24 (t, 2H, *J* = 8.0 Hz, *H*_15_), 8.00 (td, 2H, *J* = 8.0, 8.0, 1.5 Hz, *H*_9_), 7.96 (td, 2H, *J* = 8.0, 8.0, 1.5 Hz, *H*_22_), 7.85 (dd, 2H, *J* = 5.8, 1.5 Hz, *H*_2_), 7.75 (ddd, 2H, *J* = 8.2, 5.6, 2.6 Hz, *H*_16_), 7.69 (dd, 2H, *J *= 5.7, 1.5 Hz, *H*_21_), 7.43 (ddd, 2H, *J* = 7.5, 5.8, 1.3 Hz, *H*_4_), 7.20–7.30 (m, 4H, *H*_14,23_), 7.19 ppm (ddd, 2H, *J* = 7.4, 5.8, 1.3 Hz, *H*_3_). ^13^C{^1^H} NMR (100 MHz, CD_3_CN, 22 °C): δ 158.8, 158.3, 154.6, 152.4 (*C*_2_), 147.2, 139.9 (*C*_15_), 139.3 (*C*_22_), 134.2 (*C*_9_), 133.4, 131.0, 130.8, 130.3, 128.8 (*C*_4_), 128.7, 126.5, 126.4, 125.7 (*C*_17/20_), 125.6 (*C*_17/20_), 125.2 (*C*_5_), 125.1 (*C*_10_), 124.4 (*C*_8_), 122.6 ppm. HR‐MS (ESI‐TOF/MS, m/z) calcd. for RuC_46_H_30_N_6_ [M]^2+^, 384.0788; found 384.0767. UV‐Vis (CH_3_CN, 22 °C): 585 nm (MLCT; 11 800 M^−1^ cm^−1^).


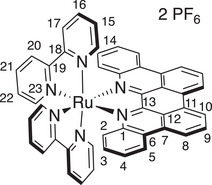


#### 4.1.5 Synthesis of [Ir(ppy)_2_(*p*‐biphe)]PF_6_

A Teflon stoppered flask was charged with a 2:1 mixture of dichloromethane/methanol (5 mL), *p*‐biphe (0.050 g, 0.14 mmol, 2.2 equiv.) and *tetrakis*(2‐(2‐pyridinyl)phenyl)diiridium(III) dichloride (0.068 g, 0.063 mmol, 1.0 equiv.). The mixture was sparged with Ar for 5 min. The flask was then heated with stirring for 6 h in an oil bath set to 50 °C. After cooling to room temperature, potassium hexafluorophosphate (0.10 g, 0.54 mmol, 8.6 equiv.) was added to the flask under a positive pressure of Ar. The resulting suspension was then filtered, and the precipitate washed with diethylether (10 mL). Diffusion of pentane vapours into a chloroform gave the product as dark, black crystalline blocks. Yield = 0.082 g (65%). ^1^H NMR (400 MHz, CD_3_CN, 22 °C): δ 8.87 (t, 4H, *J* = 8.5 Hz*, H*_8,10_), 8.74 (d, 2H, *J* = 8.3 Hz*, H*_5_), 8.25 (t, 2H, *J* = 8.1 Hz*, H*_9_), 8.19 (d, 2H, *J* = 8.7 Hz*, H*_14_), 8.00 (d, 2H, *J* = 8.6 Hz*, H*_17_), 7.68–7.79 (m, 8H, *H*_2,3,4,20_), 7.16 (t, 2H, *J* = 8.3 Hz*, H*_15_), 7.01 (t, 2H, *J* = 7.2 Hz*, H*_21_), 6.91 (t, 2H, *J* = 7.6 Hz*, H*_22_), 6.84 (t, 2H, *J* = 6.7 Hz*, H*_16_), 6.24 ppm (d, 2H, *J* = 7.6 Hz*, H*_23_). ^13^C{^1^H} NMR (100 MHz, CD_3_CN, 22 °C): δ 167.3, 157.2, 150.9, 149.9, 143.8, 143.0, 138.6, 134.2, 134.1, 130.9, 130.8, 130.2, 130.1, 129.4, 129.2, 126.5, 124.9, 124.8, 123.6, 123.4, 122.9, 122.4, 120.1 ppm. HR‐MS (ESI‐TOF/MS, m/z) calcd. for IrC_48_H_30_N_4_ [M]^+^, 855.2097; found 855.2018. UV‐Vis (CH_3_CN, 22 °C): 608 nm (MLCT; 1400 M^−1^ cm^−1^).


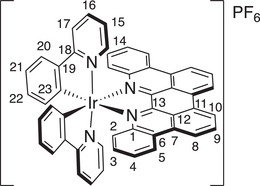


### 4.2 UV‐Vis Absorption and Luminescence Measurements

Absorption spectra were measured on a Biotek Instruments XS spectrometer, using quartz cuvettes of 1 cm pathlength. Steady‐state luminescence spectra were measured using a Horiba Fluorolog‐3 spectrofluorimeter, fitted with a red‐sensitive Hamamatsu R928 photomultiplier tube in the first instance; the spectra shown are corrected for the wavelength dependence of the detector, and the quoted emission maxima refer to the values after correction. The low‐energy emission spectra of the Cu(I) and Ir(III) complexes tail far into the NIR region, where the response of the PMT rapidly falls off. The emission spectra shown in Figure 8 were therefore recorded on the same instrument but using a back‐illuminated deep depletion CCD detector (Horiba Synapse) that offers good sensitivity up to 1000 nm. The emission maxima of the 0,0 bands matched closely (to within ±1 nm) to those from the corrected spectra recorded with the PMT. Samples for emission measurements were contained within quartz cuvettes of 1 cm pathlength modified with appropriate glassware to allow connection to a high‐vacuum line. Degassing was achieved via a minimum of three freeze‐pump‐thaw cycles whilst connected to the vacuum manifold; final vapor pressure at 77 K was <5 × 10^−2 ^mbar, as monitored using a Pirani gauge. Measurements were made under this vacuum followed by corresponding aerated measurements upon allowing the solutions to equilibrate with air. Luminescence quantum yields were determined through the following equation:

$$\Phi_{sample}=\left(I_{sample}/I_{std}\right)\times \left(A_{std}/A_{sample}\right)\times {\left(n_{sample}/n_{std}\right)}^{2}\times \Phi_{std}$$

where *I*_sample_ and *I*_std_ are the integrated areas under the corrected emission spectra of sample and standard, *A*_sample_ and *A*_std_ are the respective absorbances at the excitation wavelength employed, *n*_sample_ and *n*_std_ are the refractive indices of CH_2_Cl_2_ and H_2_O respectively, and Φ_std_ is the quantum yield of the standard. The standard used was [Ru(bpy)_3_]Cl_2_ (bpy = 2,2′‐bipyridine) in air‐equilibrated aqueous solution, for which the now widely accepted value of Φ is 0.040 ± 0.002.^[^ ^59^ ^]^ The estimated uncertainty on the quantum yields obtained in this way on the instrumentation employed is up to ± 20%. The quantum yield of the Ir(III) complex measured in this way using the visible‐light CCD detector was determined to be 0.0020, but that value is clearly an underestimate. Based on the proportion of the integrated emission at λ > 950 nm in the composite spectrum of Figure S14, we estimate the quantum yield to be around 1.3 × that based on emission < 950 nm, giving a value of 0.0026.

The luminescence lifetimes of the complexes were measured by time‐correlated single‐photon counting, following excitation at 405 nm with a pulsed‐diode laser. The emitted light was detected at 90° using a Peltier‐cooled R928 PMT after passage through a monochromator. For all measurements, the decays were much longer than the instrument response, and data were therefore analyzed by tail fitting rather than by deconvolution of the response function. The estimated uncertainty in the quoted lifetimes is ± 10% or better.

### 4.3 Computational Details

All computational work was carried out using Orca v.5.0.2.^[^ ^60^, ^61^, ^62^ ^]^ DFT optimizations were done at the PBE0/def2SVP^[^ ^63^, ^64^, ^65^ ^]^ level of theory ([Ru(bpy)_2_(*p*‐biphe)]^2+^; [Ir(ppy)_2_(*p*‐biphe)]^+^) or O3LYP/def2SVP^[^ ^65^, ^66^ ^]^ ([Cu(*p*‐biphe)_2_]^+^; [(P^P)Cu(*p*‐biphe)]^+^), using the SMD^[^ ^67^ ^]^ solvent model (CH_3_CN) and Grimme's D3 dispersion correction with Becke‐Johnson damping (D3BJ).^[^ ^68^ ^]^ The def2‐ECP^[^ ^69^ ^]^ is automatically used for Ru and Ir. The following convergence criteria were used during optimization: tightopt, tightscf. A frequency calculation was performed on all optimizations to confirm the optimized geometries were at a minimum. The lowest energy triplet states were calculated using the same method. TD‐DFT and single point calculations were performed at the PBE0 level of theory,^[^ ^63^, ^64^ ^]^ using ZORA^[^ ^70^ ^]^ and the ZORA‐DEF2‐TZVP^[^ ^65^ ^]^ basis set on all atoms to account for relativistic effects for [Cu(*p*‐biphe)_2_]^+^ and [(P^P)Cu(*p*‐biphe)]^+^. TD‐DFT and single point calculations were performed at the O3LYP^[^ ^66^ ^]^ ([Ru(bpy)_2_(*p*‐biphe)]^2+^) or M06^[^ ^71^ ^]^ ([Ir(ppy)_2_(*p*‐biphe)]^+^) level of theory, using ZORA^[^ ^70^ ^]^ and the OLD‐ZORA‐TZVP basis set on all atoms to account for relativistic effects. Also incorporated was the SMD^[^ ^67^ ^]^ solvent model (DCM: [Cu(*p*‐biphe)_2_]^+^, [(P^P)Cu(*p*‐biphe)]^+^; CH_3_CN: [Ru(bpy)_2_(*p*‐biphe)]^2+^, [Ir(ppy)_2_(*p*‐biphe)]^+^), and the resolution of identity approximation (RIJCOSX),^[^ ^72^ ^]^ to speed up the calculations, with the SARC/J auxiliary basis set.^[^ ^73^ ^]^ For TDDFT and single point calculations on [Ru(bpy)_2_(*p*‐biphe)]^2+^ and [Ir(ppy)_2_(*p*‐biphe)]^+^, a special grid was placed on the heavy transition metal (RIJCOSX; intaccx: 4.34, 4.34, 4.67; gridx: 2,2,2; specialgridintacc: 9),^[^ ^72^ ^]^ along with SARC‐TZVP^[^ ^65^ ^]^ and the corresponding auxiliary basis sets^[^ ^73^, ^74^, ^75^, ^76^, ^77^ ^]^ on the Ru and Ir atoms. SCF convergence was set to TightSCF. Molecular orbital analyses were carried out using the Hirshfeld partition method^[^ ^78^ ^]^ available in Multiwfn software.^[^ ^79^ ^]^ Avagadro^[^ ^80^ ^]^ was employed to visualize the molecular orbitals. The results of TD‐DFT were analyzed using Multiwfn^[^ ^79^ ^]^ and spin density maps were printed out using Gabedit.^[^ ^81^ ^]^ To calculate ground‐state, excited‐state, and reorganization energies, the following, previously established, protocol was followed: (1) The S_0_ geometry was first optimized by restricted DFT (charge = 1 or 2, multiplicity = 1) using the crystal structure coordinates as starting input. The results were compared to the crystal structure to verify the accuracy of the level of theory. Using this verified method, the rest of the complexes S_0_ geometries were optimized and compared to crystal structures where available. The T_1_ geometries were optimized with unrestricted DFT (charge = 1 or 2, multiplicity = 3) using the optimized S_0_ geometry as starting input. Frequency calculations were carried out with each optimization to confirm that these structures are at a minimum. (2) The electronic energies, *E*(S_0_) and *E*(T_1_), obtained from the single point calculations of S_0_ and T_1_, in their respective minimum, were used to estimate the adiabatic energy (*E* ^adia^), where *E* ^adia ^= *E*(T_1_) – *E*(S_0_). (3) TD‐DFT was then carried out on the first 75 S*_n_* ← S_0_ singlet‐singlet transitions with charge = 1 or 2, and multiplicity = 1. Population analysis was performed to determine the relative molecular fragment contributions to the frontier MOs which contribute to the electronic transitions. (4) *E* ^vert‐phos^ (T_1_ → T_1_@S_0_) was estimated as the ΔSCF between single point energies of the T_1_ (charge = 1 or 2, multiplicity = 3) and T_1_@S_0_ (charge = 1 or 2, multiplicity = 1), both at the optimized T_1_ geometry.

### 4.4 X‐Ray Crystallography Data

X‐ray crystal structure data were collected from multi‐faceted crystals of suitable size and quality, selected from a representative sample of crystals of the same habit using an optical microscope. In each case, crystals were mounted on MiTiGen loops and data collection carried out in a cold stream of nitrogen (150 K; Bruker D8 QUEST ECO; Mo Kα radiation). All diffractometer manipulations were carried out using Bruker APEX3 software.^[^ ^82^ ^]^ Structure solution and refinement was carried out using XS, XT and XL software, embedded within the Olex2 GUI.^[^ ^83^ ^]^ For each structure, the absence of additional symmetry was confirmed using ADDSYM incorporated in the PLATON program.^[^ ^84^ ^]^

#### 4.4.1 *p*‐Biphe (CCDC 2431210)

Crystals suitable for X‐ray diffraction were grown through the slow evaporation of a chloroform solution containing *p*‐Biphe. Yellow plates; C_28_H_16_N_2_Cl_6_, 453.13 g/mol, triclinic, space group *P*‐1; *a* = 9.836(3) Å, *b* = 9.847(4) Å, *c* = 13.567(5) Å, *α =* 106.483(15)°, *β* = 94.182(14)°, *γ* = 93.908(14)°, V = 1251.2(8) Å^3^; Z = 2, *ρ*_calcd_ = 1.574 g cm^−3^; crystal dimensions 0.140 × 0.050 × 0.020 mm; 2*θ*_max_ = 50.738°; 33 816 reflections, 4574 independent (R_int_ = 0.0789, intrinsic phasing; absorption coeff (μ = 0.710 mm^−1^), absorption correction semi‐empirical from equivalents (SADABS); refinement (against F_o_^2^) with SHELXTL V6.1, 325 parameters, 0 restraints, *R_1_* = 0.0926 (*I* > 2*σ*) and *wR_2_* = 0.2298 (all data), Goof = 1.184, residual electron density 0.96/−0.62 Å^−3^.

#### 4.4.2 [Cu(*p*‐biphe)_2_]PF_6_ (CCDC 2431207)

Crystals suitable for X‐ray diffraction were grown through the layering of diethyl ether over a solution of [Cu(*p*‐biphe)_2_]PF_6_ in acetonitrile. Green blocks; C_54_H_31_CuF_6_N_5_P, 958.35 g/mol, triclinic, space group *P*‐1; *a* = 9.1606(5) Å, *b* = 14.0928(7) Å, *c* = 16.7140(9) Å, *α =* 74.100(2)°, *β* = 75.446(2)°, *γ* = 86.265(2)°, V = 2008.59(19) Å^3^; Z = 2, *ρ*_calcd_ = 1.585 g cm^−3^; crystal dimensions 0.145 × 0.136 × 0.126 mm; 2*θ*_max_ = 54.308°; 69 594 reflections, 8882 independent (R_int_ = 0.0325, intrinsic phasing; absorption coeff (μ = 0.661 mm^−1^), absorption correction semi‐empirical from equivalents (SADABS); refinement (against F_o_^2^) with SHELXTL V6.1, 605 parameters, 0 restraints, *R_1_* = 0.0391 (*I* > 2*σ*; 7984 reflections) and *wR_2_* = 0.1020 (all data), Goof = 1.035, residual electron density 0.65/‐0.47 Å^−3^.

#### 4.4.3 [Cu(P^P)(*p*‐biphe)]PF_6_ (CCDC 2431208)

Crystals suitable for X‐ray diffraction were grown through the layering of hexanes over a solution of [Cu(P^P)(*p*‐biphe)]PF_6_ in dichloromethane. Brown blocks; C_66_H_48_Cl_2_CuF_6_N_2_OP_3_, 1226.41 g/mol, triclinic, space group *P*‐1; *a* = 10.802(3) Å, *b* = 15.453(4) Å, *c* = 17.243(5) Å, *α =* 86.42(2)°, *β* = 77.35(2), *γ* = 80.422(18), V = 2768.3(13) Å^3^; Z = 2, *ρ*_calcd_ = 1.471 g cm^−3^; crystal dimensions 0.320 × 0.270 × 0.070 mm; 2*θ*_max_ = 49.634°; 66 867 reflections, 9217 independent (R_int_ = 0.0565, intrinsic phasing; absorption coeff (μ = 0.646 mm^−1^), absorption correction semi‐empirical from equivalents (SADABS); refinement (against F_o_^2^) with SHELXTL V6.1, 732 parameters, 0 restraints, *R_1_* = 0.0856 (*I* > 2*σ*; 8022 reflections) and *wR_2_* = 0.2041 (all data), Goof = 1.153, residual electron density 1.50/‐1.46 Å^−3^.

#### 4.4.4 [Ru(bpy)_2_(*p*‐biphe)](PF_6_)_2_ (CCDC 2431209)

Crystals suitable for X‐ray diffraction were grown through the layering of diethyl ether over a solution of [Ru(bpy)_2_(*p*‐biphe)](PF_6_)_2_ in acetonitrile. Purple needles; C_46_H_30_F_12_N_6_P_2_Ru, 1057.77 g/mol, monoclinic, space group *P*2_1_/c; *a* = 9.7888(18) Å, *b* = 17.774(5) Å, *c* = 28.566(5) Å, *α = γ* = 90°, *β* = 90.006(12), V = 4970.3(18) Å^3^; Z = 4, *ρ*_calcd_ = 1.414 g cm^−3^; crystal dimensions 0.760 × 0.030 × 0.020 mm; 2*θ*_max_ = 49.6°; 103 210 reflections, 8474 independent (R_int_ = 0.0863, intrinsic phasing; absorption coeff (μ = 0.462 mm^−1^), absorption correction semi‐empirical from equivalents (SADABS); refinement (against F_o_^2^) with SHELXTL V6.1, 604 parameters, 6 restraints, *R_1_* = 0.1156 (*I* > 2*σ*) and *wR_2_* = 0.2538 (all data), Goof = 1.199, residual electron density 1.96/−2.66 Å^−3^. SIMU was applied to C(33) and C(30) to account for oblate ellipsoid shape.

#### 4.4.5 [Ir(ppy)_2_(*p*‐biphe)]PF_6_ (CCDC 2431318)

Crystals suitable for X‐ray diffraction were grown through the layering of diethyl ether over a solution of [Ir(ppy)_2_(*p*‐biphe)]PF_6_ in acetonitrile. Black blocks; C_100_H_66_F_12_N_10_P_2_F_12_Ir, 2081.96 g/mol, monoclinic, space group *P*2_1_/*c*; *a* = 14.793(3) Å, *b* = 10.023(2) Å, *c* = 27.709(7) Å, *α = γ* = 90°, *β* = 101.479, V = 4026.2(15) Å^3^; Z = 2, *ρ*_calcd_ = 1.717 g cm^−3^; crystal dimensions 0.630 × 0.490 × 0.460 mm; 2*θ*_max_ = 56.626°; 154 718 reflections, 9994 independent (R_int_ = 0.0197, intrinsic phasing; absorption coeff (μ = 3.429 mm^−1^), absorption correction semi‐empirical from equivalents (SADABS); refinement (against F_o_^2^) with SHELXTL V6.1, 569 parameters, 0 restraints, *R_1_* = 0.0174 (*I* > 2*σ*) and *wR_2_* = 0.0405 (all data), Goof = 1.164, residual electron density 0.63/−1.17 Å^−3^.

## Supporting Information

Additional X‐ray figures, computational discussion, supporting figures and tables; multi‐nuclear NMR and HR‐MS spectra of all new compounds; crystallographic information files containing all X‐ray data. Deposition Number(s) 2431207‐2431210 and 2431318 contain(s) the supplementary crystallographic data for this paper. These data are provided free of charge by the joint Cambridge Crystallographic Data center and Fachinformationszentrum Karlsruhe Access Structures service.

## Author Contributions

The manuscript was written through contributions of all authors. All authors have given approval to the final version of the manuscript.

## Conflict of Interest

The authors declare no conflict of interest.

## Supporting information



Supporting Information

Supporting Information

## Data Availability

The data underlying this study are available in the published article and its Supporting Information.
