# Supplementary material for: In Pursuit of Low Energy Phosphorescence: Late Metal Coordination Complexes of the Planar, π‐extended Bipyridyl Ligand 6,6′,7,7′‐Biphenanthridine
Source: Chemistry. 2025 Sep 8;31(63):e01802. doi: 10.1002/chem.202501802 (PMC12619056; doi:10.1002/chem.202501802)
Supplement: Supplementary file 1 — Supporting Information [file CHEM-31-e01802-s001.pdf]

Supporting Information

for

Investigating Strong Absorbance and Low Energy Phosphorescence of Late Metal Coordination Complexes of the Planar, π-extended Bipyridyl Ligand 6,6',7,7'-Biphenanthridine

Dion B. Nemez,[a] Robert J. Ortiz,[a] Keighlynn A. Veilleux,[a] J. A. Gareth Williams,[b] and David E. Herbert[a*]*

[a] Department of Chemistry and the Manitoba Institute for Materials, University of Manitoba, 144 Dysart Road, Winnipeg, Manitoba, R3T 2N2, Canada; *david.herbert@umanitoba.ca

[c] Department of Chemistry, Durham University, Durham, DH1 3LE, UK; *j.a.g.williams@durham.ac.uk

Table of Contents

Figure S1. Absorption spectra of *p*-biphe in various solvents. 5
Figure S2. Graphical representation of the bathochromic response at lowest energy wavelength to solvent polarity explained either via (a) acceptor number of the solvent or (b) Reichardt's E_T values.[1] 6
Figure S3. (a) Changes in the absorption spectra of *p*-biphe in acetonitrile (blue trace) at 295 K following incremental addition of trifluoromethane sulfonic acid (black traces). The red trace shows the absorption spectrum following addition of one full equivalent, which corresponds to that of the isolated monoprotonated species. Further addition does not lead to further changes; the green trace is after addition of 1.5 equivalents. (b) Changes in the emission spectra (λ_{ex} = 415 nm) showing the same cessation of changes after reaching one equivalent of acid. The red checkered trace shows the intensity gain when excited at the λ_{max} of the mono-protonated species (λ_{ex} = 530 nm). 7
Figure S4. Two views of the lowest unoccupied molecular orbital (LUMO) of *p*-biphe. See reference[2] for computational details. 7
Figure S5. Overlay of the cationic portions of the crystal structures of the acetonitrile solvate of *bis*(*p*-biphe) copper(I) hexafluorophosphate ([Cu(*p*-biphe)$_2$]PF_6) and the tetrahydrofuran solvate of [Cu(*p*-biphe)$_2$]PF_6 from ref[3] .. 8
Figure S6. Octahedricity parameters of [Ru(bpy)$_2$(*p*-biphe)](PF_6)$_2$ and [Ir(ppy)$_2$(*p*-biphe)]PF_6. 9
Table S1. Tabulated octahedricity parameters calculated using Octadist[4] 10
Figure S7. Space-filling diagram of the cationic portions of [Ru(bpy)$_2$(*p*-biphe)](PF_6)$_2$ and [Ir(ppy)$_2$(*p*-biphe)]PF_6 highlighting the angle of the *p*-biphe ligand with respect to the rest of the coordination sphere of the metals. 10
Table S2. Fragment contributions (%) to the frontier MOs of [Cu(*p*-biphe)$_2$]$^+$ using the Hirshfeld[5] atomic population method. [a] 11
[a] RIJCOSX-ZORA-SMD-PBE0/ZORA-def2TZVP+SARC/J//SMD-O3LYP-D4/def2-SVP 11
Table S3. Fragment contributions (%) to the frontier MOs of [(P^P)Cu(*p*-biphe)]$^+$ using the Hirshfeld's atomic population method.[5] [a] 11
[a] RIJCOSX-ZORA-SMD-PBE0/ZORA-def2TZVP+SARC/J//SMD-O3LYP-D4/def2-SVP 11
Table S4. Fragment contributions (%) to the frontier MOs of [Ru(bpy)$_2$(*p*-biphe)]$^{2+}$ using the Hirshfeld[5] atomic population method. [a] 12
[a] RIJCOSX-ZORA-SMD-O3LYP/old-ZORA-TZVP+SARC/J//SMD-PBE0-D3(BJ)/def2-SVP 12
Figure S8. Figure relating the colour coding from Table S4 above for [Ru(bpy)$_2$(*p*-biphe)]$^{2+}$. 12
Table S5. Fragment contributions (%) to the frontier MOs of [Ir(ppy)$_2$(*p*-biphe)]$^+$ using the Hirshfeld[5] atomic population method. [a] 13
Figure S9. Figure relating the colour coding from Table S5 above for [Ir(ppy)$_2$(*p*-biphe)]$^+$. 13
Table S6. TD-DFT calculated electronic transitions along with their corresponding excitation energies and oscillator strengths (f_{osc} > 0.04) for [(P^P)Cu(*p*-biphe)]$^+$ (contributions > 10%). Only the first 75 transitions were considered. 14
Figure S10. Comparison of the experimental (-) with the computed scalar-only (---) spectra, and vertical energy transitions (red solid line) for [(P^P)Cu(*p*-biphe)]$^+$ in dichloromethane (FWHM = 0.35 eV; RIJCOSX-ZORA-SMD-PBE0/ZORA-def2TZVP+SARC/J//SMD-O3LYP-D4/def2-SVP). Only transitions with oscillator strengths > 0.04 are shown. Electron-hole density maps shown for three major lowest energy transitions. 16
Table S7. TD-DFT calculated electronic transitions along with their corresponding excitation energies and oscillator strengths (f_{osc} > 0.04) for [Cu(*p*-biphe)$_2$]$^+$ (contributions > 10%). Only the first 75 transitions were considered. 17
Figure S11. Comparison of the experimental (-) with the computed scalar-only (---) spectra, and vertical energy transitions (red solid line) for [Cu(*p*-biphe)$_2$]$^+$ in dichloromethane (FWHM = 0.35 eV; RIJCOSX-ZORA-SMD-PBE0/ZORA-def2TZVP+SARC/J//SMD-O3LYP-D4/def2-SVP). Only transitions with oscillator strengths > 0.04 are shown and electron hole density maps are shown for four of the major low energy transitions. 18
Table S8. TD-DFT calculated electronic transitions along with their corresponding excitation energies and oscillator strengths (f_{osc} > 0.04; f_{osc} > 0.01 at lower energy than 500 nm) for [Ru(bpy)$_2$(biphe)][PF_6]$_2$ (contributions > 10%). Only the first 50 transitions were considered. 19
Figure S12. Comparison of the experimental (-) with the computed scalar-only (---) spectra, and vertical energy transitions (red solid line) for [Ru(bpy)$_2$(biphe)][PF_6]$_2$ in acetonitrile (FWHM = 0.35 eV; RIJCOSX-ZORA-SMD-O3LYP/old-ZORA-TZVP+SARC/J//SMD-PBE0-D3(BJ)/def2-SVP). At energies < 2.5 eV only transitions with

oscillator strengths > 0.01 are shown; at energies > 2.5 eV only transitions with oscillator strengths > 0.04 are shown. Electron-hole density maps shown for three major lowest energy transitions. 20
Table S9. TD-DFT calculated electronic transitions along with their corresponding excitation energies and oscillator strengths ((f_{osc} > 0.04; f_{osc} > 0.03 at lower energy than 500 nm) for [Ir(ppy)$_2$(*p*-biphe)]PF$_6$ (contributions > 10%). Only the first 50 transitions were considered. 21
Figure S13. Comparison of the experimental (-) with the computed scalar-only (---) spectra, and vertical energy transitions (red solid line) for [Ir(ppy)$_2$(*p*-biphe)]PF$_6$ in acetonitrile (FWHM = 0.35 eV; RIJCOSX-ZORA-SMD-M06/OLD-ZORA-TZVP+SARC/J//SMD-PBE0-D3(BJ)/def2-SVP). Only transitions with oscillator strengths > 0.03 are shown and electron hole density maps (isosurface = 0.002) shown for the three major lowest energy transitions. 23
Figure S14. Emission spectra of [Ir(ppy)$_2$(*p*-biphe)]PF$_6$ recorded in in CH_2Cl_2 at 295 K using a CCD detector sensitive to the visible range of the electromagnetic spectrum (blue) and a NIR sensitive PMT detector (red). The 3rd and 4th vibrational shoulders are evident > 1000 nm. 24
Figure S15. Spectra of the weak fluorescence displayed by the Cu(I) complexes and the Ru(II) complex when excited at higher energies (*i.e.*, *not* into the low-energy absorption band assigned as MLCT), at 295 K in CH_2Cl_2 (Cu) or MeCN (Ru) respectively (red lines). The corresponding excitation spectra registered at λ_{em} = 580 nm are shown as dashed black lines, and the absorption spectra are in blue where asterisks mark the low-energy bands that are missing from the excitation spectra. The emission is tentatively attributed to *p*-biphe-based ligand-centred states. 25
Figure S16. Spin density plot of [(P^P)Cu(*p*-biphe)]$^+$ (right) and [Cu(*p*-biphe)$_2$]$^+$ (left). 26
Isosurface value = 0.004; RIJCOSX-ZORA-SMD-PBE0/ZORA-def2TZVP+SARC/J//SMD-O3LYP-D4/def2-SVP 26
Figure S17. Spin density plot of [Ru(bpy)$_2$(*p*-biphe)]$^{2+}$ (left) and [Ir(ppy)$_2$(*p*-biphe)]$^+$ (right). 27
Table S10. Metal Mulliken populations in the spin-density plots shown in Figures S14 and S15. 28
Table S11. Calculated photophysical parameters from single point calculations in eV. 28
Table S12. DFT (SMD-PBE0-D3(BJ)/def2-SVP) optimized ground state and lowest energy excited triplet state bond lengths (Å) and angles (°). 29
Table S13. DFT (SMD-PBE0-D3(BJ)/def2-SVP) optimized ground state and lowest energy excited triplet state bond lengths (Å) and angles (°). 30
Figure S18. Octahedricity parameters of [Ru(bpy)$_2$(*p*-biphe)]$^{2+}$ comparing XRD with the computationally determined ground state and lowest energy triplet state geometry (SMD-PBE0-D3(BJ)/def2-SVP) 31
Figure S19. Octahedricity parameters of [Ir(ppy)$_2$(*p*-biphe)]$^+$ comparing XRD with the computationally determined ground state and lowest energy triplet state geometry (SMD-PBE0-D3(BJ)/def2-SVP) 32
Table S14. Tabulated Cyclic Voltammetry (CV) and Spectroscopic Data[a] 33
Figure S20. Correlation between (i) absorption and (ii) emission energies and redox energy $\Delta E_{1/2}$ for (a) [Ru(3,3'-biisoquinoline)$_3$]$^{2+}$, (b) [Ru(3,3'-biisooquinoline)$_2$(2,2'-bipyridine)]$^{2+}$, (c) [Ru(1,10-phenanthroline)$_3$]$^{2+}$, (d) [Ru(2,2'-bipyridine)$_2$(4,4'-diphenyl-2-2'-bipyridine)]$^{2+}$, (e) [Ru(2,2'-bipyridine)$_2$(2,2'-bipyrimidine)]$^{2+}$, (f) [Ru(2,2'-bipyridine)$_2$(2-(2-pyridyl)-quinoline)]$^{2+}$, (g) [Ru(2,2'-bipyridine)$_2$(2,2'-biquinoline)]$^{2+}$, (h) [Ru(2,2'-bipyridine)$_2$(2,2'-bipyrazine)]$^{2+}$, (i) [Ru(2,2'-bipyridine)$_2$(dipyrido[3,2-*a*:2',3'-*c*]phenazine)]$^{2+}$, (j) [Ru(2,2'-bipyridine)$_2$(dipyrido[3,2-*c*:2',3'-*e*]pyridazine)]$^{2+}$, (k) [Ru(2,2'-bipyridine)$_3$]$^{2+}$, (l) [Ru(6,6'-biphenanthridine)$_3$]$^{2+}$ o, (m) [Ru(2,2'-bipyridine)$_2$(6,6'-biphenanthridine)]$^{2+}$ o, (n) [Ru(2,2'-bipyridine)$_2$(tetra-*peri*-(*tert*-butyl-benzo)-di-peri-(pyrimidino)-coronene)]$^{2+}$,[6] (o) [Ru(3,3'-biisooquinoline)$_3$]$^{2+}$, (p) Ru(bpy)$_2$(*p*-biphe)$^{2+}$ o, and (q) Ir(ppy)$_2$(*p*-biphe)$^+$ o. Both the adaptation of the figure and the acquisition of the data (unless otherwise cited) was from Juris *et al*.[7] Line of best fit was generated using only open circles. 34

NMR Spectra 35

Figure S21. ^{1}H NMR (500 MHz, 22 °C, DMSO-d_6) of *p*-biphe. 35
Figure S22. ^{13}C NMR (125 MHz, 22 °C, DMSO-d_6) of *p*-biphe. 35
Figure S23. ^{1}H-^{13}C HSQC NMR (22 °C, DMSO-d_6) of *p*-biphe. 36
Figure S24. ^{1}H-^{1}H COSY NMR (22 °C, DMSO-d_6) of *p*-biphe. 36
Figure S25. ^{1}H-^{13}C HMBC NMR (22 °C, DMSO-d_6) of *p*-biphe. 37
Figure S26. ^{1}H NMR (400 MHz, 22 °C, CD_2Cl_2) of [Cu(*p*-biphe)$_2$]PF$_6$. 37
Figure S27. ^{13}C NMR (100 MHz, 22 °C, CD_2Cl_2) of [Cu(*p*-biphe)$_2$]PF$_6$. 38
Figure S28. ^{1}H-^{13}C HSQC NMR (22 °C, CD_2Cl_2) of [Cu(*p*-biphe)$_2$]PF$_6$. 38

Figure S29. ^{1}H-^{1}H COSY NMR (22 °C, CD_2Cl_2) of [Cu(*p*-biphe)$_2$]PF_6. ... 39
Figure S30. ^{1}H-^{13}C HMBC NMR (22 °C, CD_2Cl_2) of [Cu(*p*-biphe)$_2$]PF_6. ... 39
Figure S31. ^{1}H NMR (500 MHz, 22 °C, CD_2Cl_2) of [(P^P)Cu(*p*-biphe)]PF_6. ... 40
Figure S32. ^{13}C NMR (125 MHz, 22 °C, CD_2Cl_2) of [(P^P)Cu(*p*-biphe)]PF_6. ... 40
Figure S33. ^{1}H-^{13}C HSQC NMR (22 °C, CD_2Cl_2) of [(P^P)Cu(*p*-biphe)]PF_6. ... 41
Figure S34. ^{1}H-^{1}H COSY NMR (22 °C, CD_2Cl_2) of [(P^P)Cu(*p*-biphe)]PF_6. ... 41
Figure S35. ^{1}H-^{13}C HMBC NMR (22 °C, CD_2Cl_2) of [(P^P)Cu(*p*-biphe)]PF_6. ... 42
Figure S36. ^{1}H NMR (400 MHz, 22 °C, CD_3CN) of [Ru(bpy)$_2$(*p*-biphe)](PF_6)$_2$. ... 42
Figure S37. ^{13}C NMR (100 MHz, 22 °C, CD_3CN) of [Ru(bpy)$_2$(*p*-biphe)](PF_6)$_2$. ... 43
Figure S38. ^{1}H-^{13}C HSQC NMR (22 °C, CD_3CN) of [Ru(bpy)$_2$(*p*-biphe)](PF_6)$_2$. ... 43
Figure S39. ^{1}H-^{1}H COSY NMR (22 °C, CD_3CN) of [Ru(bpy)$_2$(*p*-biphe)](PF_6)$_2$. ... 44
Figure S40. ^{1}H-^{13}C HMBC NMR (22 °C, CD_3CN) of [Ru(bpy)$_2$(*p*-biphe)](PF_6)$_2$. ... 44
Figure S41. ^{1}H NMR (400 MHz, 22 °C, CD_3CN) of [Ir(ppy)$_2$(*p*-biphe)]PF_6. ... 45
Figure S42. ^{13}C NMR (100 MHz, 22 °C, CD_3CN) of [Ir(ppy)$_2$(*p*-biphe)]PF_6. ... 45
Figure S43. ^{1}H-^{13}C HSQC NMR (22 °C, CD_3CN) [Ir(ppy)$_2$(*p*-biphe)]PF_6. ... 46
Figure S44. ^{1}H-^{1}H COSY NMR (22 °C, CD_3CN) of [Ir(ppy)$_2$(*p*-biphe)]PF_6. ... 46

MASS SPECTRA ... 47

Figure S45: HR-MS (ESI-TOF/MS) of *p*-biphe. ... 47
Figure S46. HR-MS (ESI-TOF/MS) of [Cu(*p*-biphe)$_2$]PF_6. ... 48
Figure S47. HR-MS (ESI-TOF/MS) of [(P^P)Cu(*p*-biphe)]PF_6. ... 49
Figure S48. HR-MS (ESI-TOF/MS) of [Ru(bpy)$_2$(*p*-biphe)](PF_6)$_2$. ... 50
Figure S49. HR-MS (ESI-TOF/MS) of [Ir(ppy)$_2$(pBiphe)]PF_6. ... 51

SUPPLEMENTARY COMPUTATIONAL TABLES ... 52

Table S15. Comparison of experimentally and computationally[a] determined bond distances (Å) and angles (°) for [Cu(*p*-biphe)$_2$]$^+$. ... 52
[Cu(*p*-biphe)$_2$]$^+$ ... 52
[Cu(*p*-biphe)$_2$] ... 52
Table S16. Comparison of experimentally and computationally[a] determined bond distances (Å) and angles (°) for [(P^P)Cu(*p*-biphe)]$^+$. ... 53
[(P^P)Cu(*p*-biphe)] ... 53
[(P^P)Cu(*p*-biphe)] ... 53
Table S17. Comparison of experimentally and computationally[a] determined bond distances (Å) and angles (°) for [Ru(bpy)$_2$(*p*-biphe)]$^{2+}$. ... 54
[Ru(bpy)$_2$(*p*-biphe)] ... 54
[Ru(bpy)$_2$(*p*-biphe)] ... 54
Table S18. Comparison of experimentally and computationally[a] determined bond distances (Å) and angles (°) for [Ir(ppy)$_2$(*p*-biphe)]$^+$. ... 55
[Ir(ppy)$_2$(*p*-biphe)] ... 55
[Ir(ppy)$_2$(*p*-biphe)] ... 55

REFERENCES ... 56

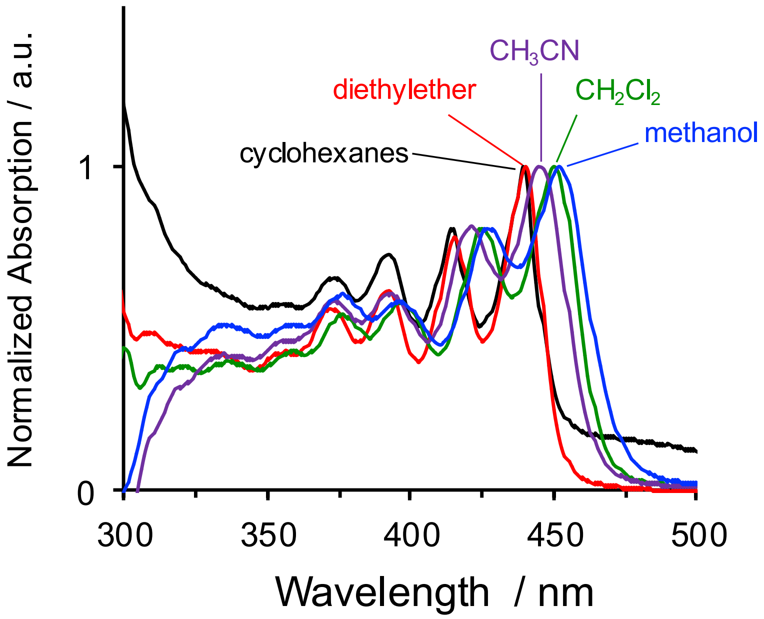

Figure S1. Absorption spectra of *p*-biphe in various solvents.

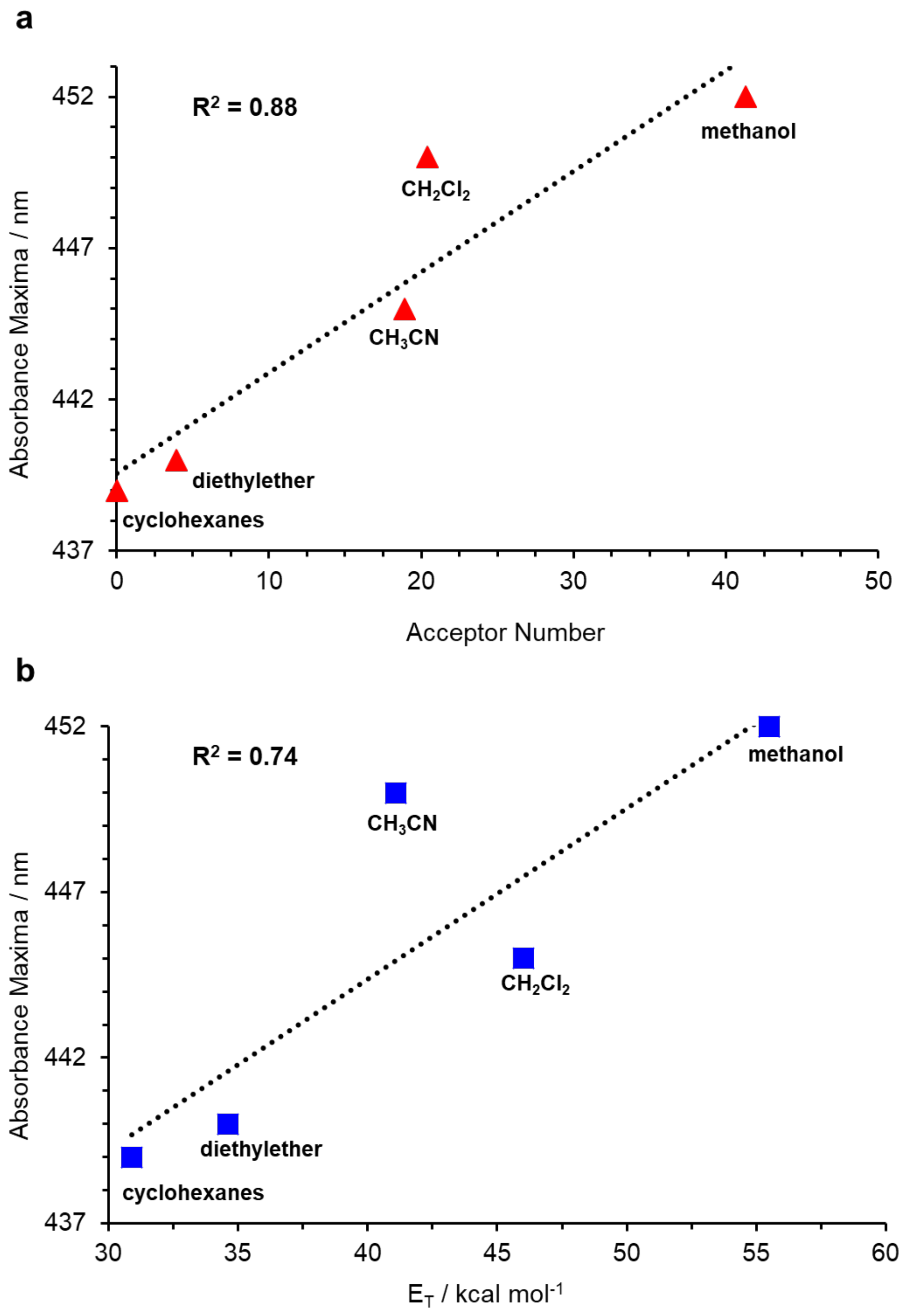

Figure S2. Graphical representation of the bathochromic response at lowest energy wavelength to solvent polarity explained either via (a) acceptor number of the solvent or (b) Reichardt's E_T values.[1]

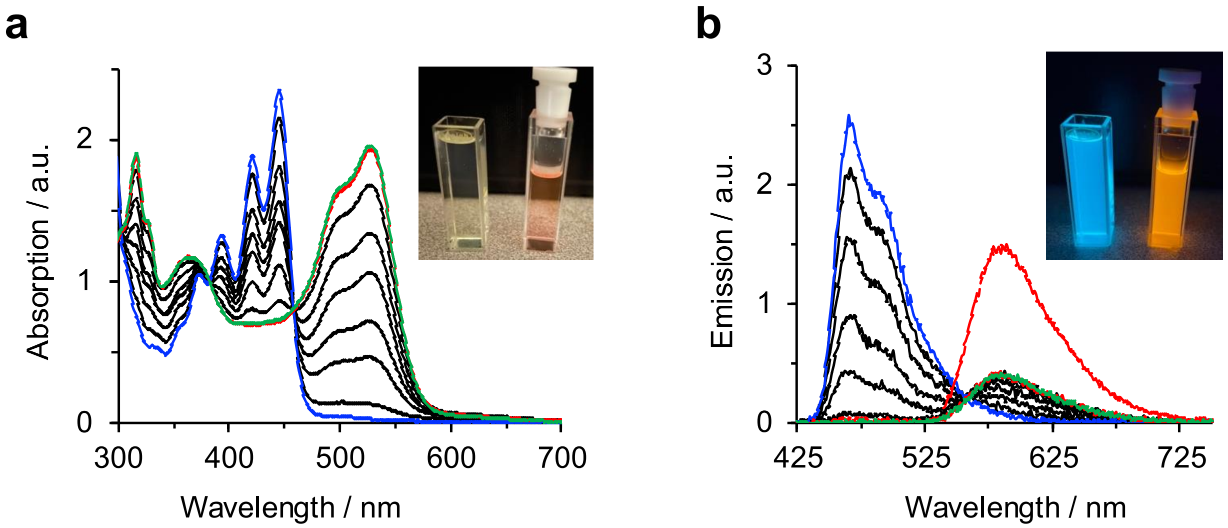

Figure S3. (a) Changes in the absorption spectra of *p*-biphe in acetonitrile (blue trace) at 295 K following incremental addition of trifluoromethane sulfonic acid (black traces). The red trace shows the absorption spectrum following addition of one full equivalent, which corresponds to that of the isolated monoprotonated species. Further addition does not lead to further changes; the green trace is after addition of 1.5 equivalents. (b) Changes in the emission spectra (λ_{ex} = 415 nm) showing the same cessation of changes after reaching one equivalent of acid. The red checkered trace shows the intensity gain when excited at the λ_{max} of the mono-protonated species (λ_{ex} = 530 nm).

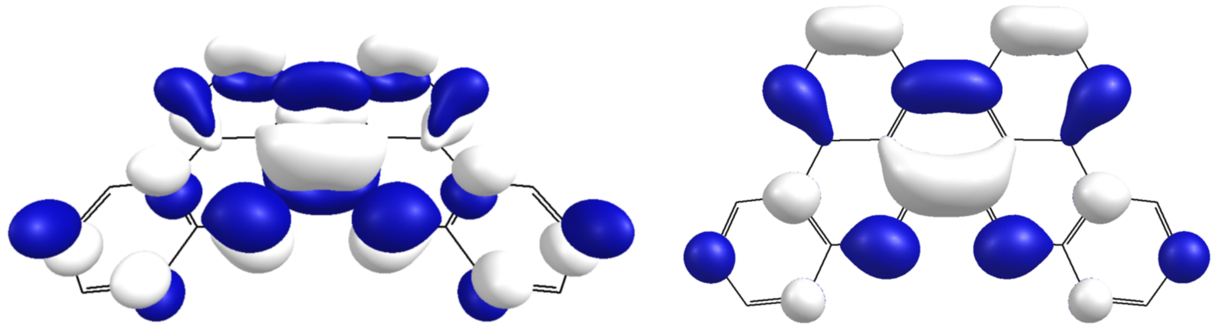

Figure S4. Two views of the lowest unoccupied molecular orbital (LUMO) of *p*-biphe. See reference[2] for computational details.

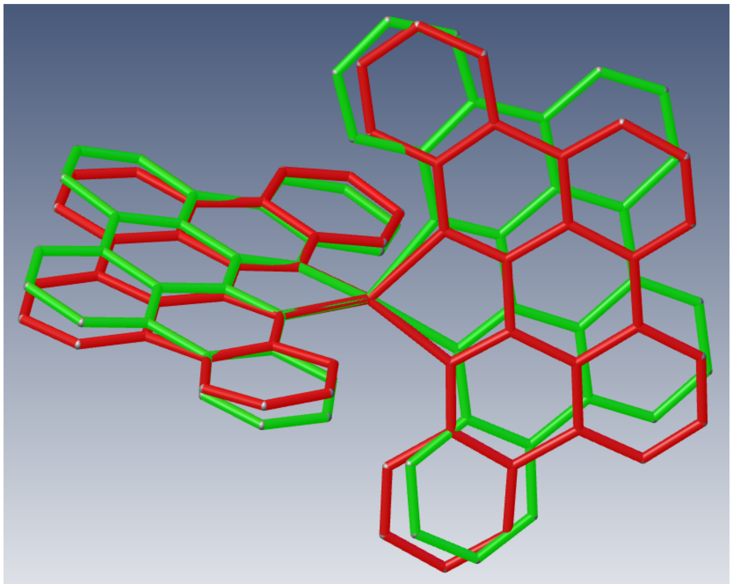

Figure S5. Overlay of the cationic portions of the crystal structures of the acetonitrile solvate of *bis*(*p*-biphe) copper(I) hexafluorophosphate ([Cu(*p*-biphe)$_2$]PF_6) and the tetrahydrofuran solvate of [Cu(*p*-biphe)$_2$]PF_6 from ref[3]

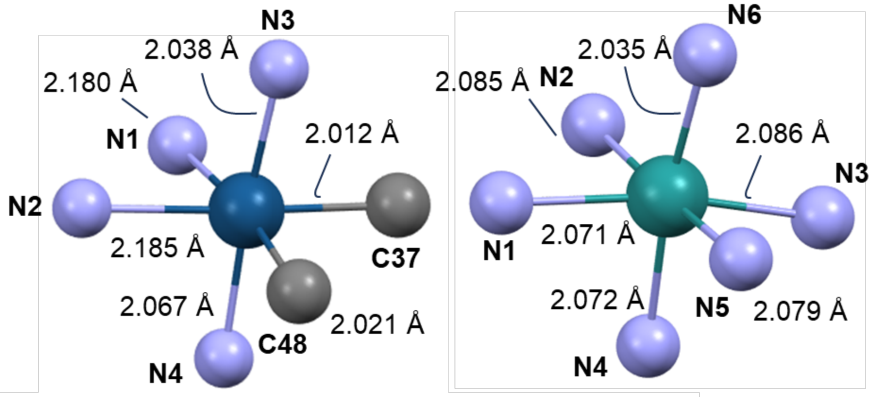

Parameter	Ir (XRD)	Ru (XRD)
d_{mean}[a] / Å	2.08	2.07
ζ[b] / Å	0.396	0.072
Δ[c]	0.00119	0.00007
Σ[d] / °	99.1	92.0
Θ[e] / °	284	245

[a] Average Metal-Ligand Distance [b] $\zeta = \sum_{i=1}^{6} |d_i - d_{\mathrm{mean}}|$ [c] $\Delta = \frac{1}{6}\sum_{i=1}^{6}(\frac{d_i - d}{d})^2$
[d] $\Sigma = \sum_{i=1}^{12} |\phi_i - 90|$ [e] $\Theta = \sum_{i=1}^{24} |\theta_i - 60|$

Figure S6. Octahedricity parameters of [Ru(bpy)$_2$(*p*-biphe)](PF$_6$)$_2$ and [Ir(ppy)$_2$(*p*-biphe)]PF$_6$.

Table S1. Tabulated octahedricity parameters calculated using Octadist[4]

Compound	d_{mean}[a] (Å)	ζ[b] (Å)	Δ[c]	Σ[d] (°)	Θ[e] (°)
$[Ru(bpy)_2(p\text{-biphe})]^{2+}$	2.071	0.0726	0.000068	92.03	244.51
$[Ir(ppy)_2(p\text{-biphe})]^{+}$	2.084	0.3957	0.001194	99.05	283.59
$[Ru(biphe)_3]^{2+}$ [f]	2.116	0.1074	0.000091	149.82	416.86
$[Ru(bpy)_2(biphe)]^{2+}$	2.080	0.2228	0.000363	119.59	330.10
$[Ru(bpy)_3]^{2+}$	2.0562	0.0000	0.000000	74.35	229.94
$Ir(ppy)_3$	2.0734	0.3424	0.000760	65.68	208.40

[a] The average metal-ligand bond length

[b] The average of the sum of the deviation of 6 unique metal–ligand bond lengths around the central metal atom (d_i) from the average value (d_{mean}). $\zeta = \sum_{i=1}^{6} |d_i - d_{mean}|$

[c] $\Delta = \frac{1}{6}\sum_{i=1}^{6}\left(\frac{d_i - d}{d}\right)^2$

[d] The sum of the deviation of 12 unique *cis* ligand–metal–ligand angles (ϕ_i) from 90°.

$$\Sigma = \sum_{i=1}^{6} |\phi_i - 90|$$

[e] The degree of trigonal distortion of the coordination geometry from an octahedron towards a trigonal prism. The Θ parameter is the sum of the deviation of 24 unique torsional angles between the ligand atoms on opposite triangular faces of the octahedron viewed along the pseudo-threefold axis (θ_i) from 60°. $\Theta = \sum_{i=1}^{6} |\theta_i - 60|$

[f] One molecule of two in the crystal structure was selected.

a b

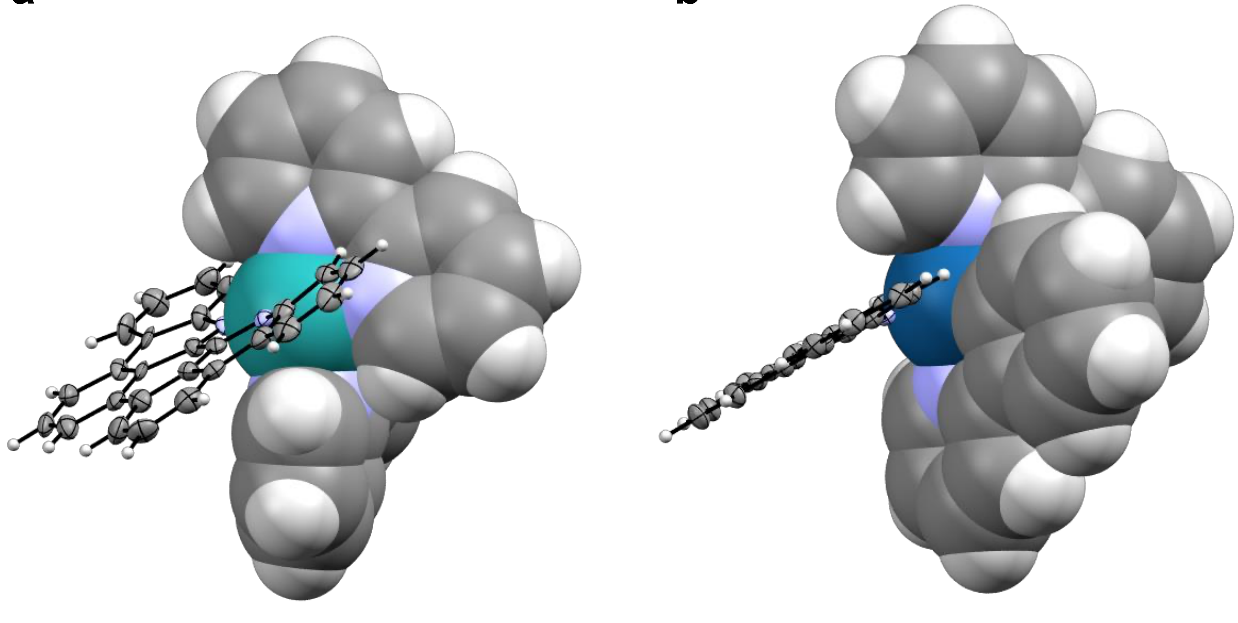

Figure S7. Space-filling diagram of the cationic portions of $[Ru(bpy)_2(p\text{-biphe})](PF_6)_2$ and $[Ir(ppy)_2(p\text{-biphe})]PF_6$ highlighting the angle of the *p*-biphe ligand with respect to the rest of the coordination sphere of the metals.

Table S2. Fragment contributions (%) to the frontier MOs of $[Cu(p\text{-biphe})_2]^+$ using the Hirshfeld[5] atomic population method. [a]

MOs	E / eV	Cu	N=C-C=N	Ar
LUMO+2	-1.96	0	3	96
LUMO+1	-3.23	6	36	58
LUMO	-3.26	5	37	58
HOMO	-6.10	61	22	17
HOMO-1	-6.38	63	19	17
HOMO-2	-6.39	6	15	80

[a] RIJCOSX-ZORA-SMD-PBE0/ZORA-def2TZVP+SARC/J//SMD-O3LYP-D4/def2-SVP

Table S3. Fragment contributions (%) to the frontier MOs of $[(P\text{^}P)Cu(p\text{-biphe})]^+$ using the Hirshfeld's atomic population method.[5] [a]

MOs	E / eV	Cu	N=C-C=N	Phen(1)	Phen(2)	$(P(Ph)_2)_2$	Xanphos
LUMO+2	-1.55	0	8	42	43	4	2
LUMO+1	-1.95	0	3	48	46	2	0
LUMO	-3.18	4	35	28	28	4	1
HOMO	-6.13	35	5	4	4	44	8
HOMO-1	-6.40	3	13	41	42	1	0
HOMO-2	-6.71	42	9	4	4	33	9

[a] RIJCOSX-ZORA-SMD-PBE0/ZORA-def2TZVP+SARC/J//SMD-O3LYP-D4/def2-SVP

Table S4. Fragment contributions (%) to the frontier MOs of $[Ru(bpy)_2(p\text{-biphe})]^{2+}$ using the Hirshfeld[5] atomic population method. [a]

MOs	E / eV	Ru	N=C-C=N	Ar(1)	Ar(2)	bipyr(1)	bipyr(2)
LUMO+2	-2.71	8	2	2	1	66	21
LUMO+1	-2.77	4	1	1	1	24	70
LUMO	-3.56	4	34	25	25	7	2
HOMO	-5.53	57	9	8	10	6	9
HOMO-1	-5.87	53	7	13	7	11	9
HOMO-2	-5.89	64	6	5	5	8	11

[a] RIJCOSX-ZORA-SMD-O3LYP/OLD-ZORA-TZVP+SARC/J//SMD-PBE0-D3(BJ)/def2-SVP

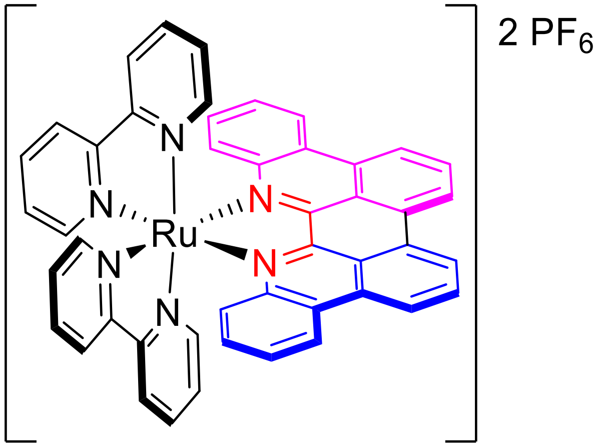

Figure S8. Figure relating the colour coding from Table S4 above for $[Ru(bpy)_2(p\text{-biphe})]^{2+}$.

Table S5. Fragment contributions (%) to the frontier MOs of $[Ir(ppy)_2(p\text{-biphe})]^+$ using the Hirshfeld[5] atomic population method. [a]

MOs	E / eV	Ir	N=C-C=N	Ar(1)	Ar(2)	ppy(1)	ppy(2)
LUMO+3	-1.66	4	1	1	1	35	57
LUMO+2	-1.75	3	1	4	5	52	35
LUMO+1	-1.80	1	3	46	43	5	2
**LUMO**	**-3.21**	**4**	**38**	**28**	**27**	**1**	**2**
**HOMO**	**-6.11**	**35**	**2**	**2**	**2**	**31**	**27**
HOMO-1	-6.33	8	11	39	37	2	3
HOMO-2	-6.53	3	1	3	4	44	46
HOMO-3	-6.76	36	2	11	20	17	15

[a] RIJCOSX-ZORA-SMD-M06/OLD-ZORA-TZVP+SARC/J//SMD-PBE0-D3(BJ)/def2-SVP

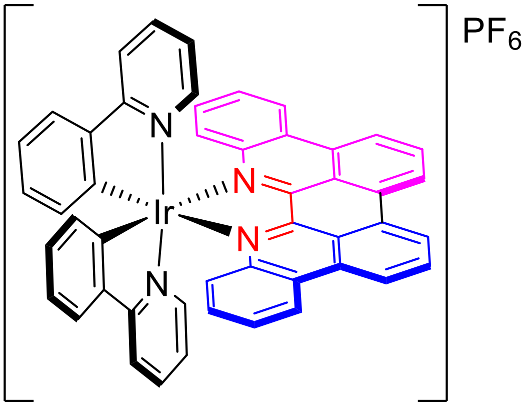

Figure S9. Figure relating the colour coding from Table S5 above for $[Ir(ppy)_2(p\text{-biphe})]^+$.

Table S6. TD-DFT calculated electronic transitions along with their corresponding excitation energies and oscillator strengths (f_{OSC} > 0.04) for [(P^P)Cu(*p*-biphe)]$^+$ (contributions > 10%). Only the first 75 transitions were considered.

No.	E / nm	f_{OSC}	Composition
**1**	**578.1**	**0.1262**	**HOMO→LUMO (92%)**
3	480.8	0.2866	HOMO-1→LUMO (68%), HOMO-3→LUMO (24%)
4	477.0	0.1186	H-2→L (55%), H-1→L (19%), H-3→L (14%)
7	377.1	0.0683	H-6→L (92%)
11	344.0	0.0487	H-7→L (15%), H-15→L (13%), H-18→L (12%), H-8→L (12%), H-20→L (11%)
18	322.3	0.0734	H-1→L+1 (48%), H-15→L (13%)
26	301.2	0.1971	H-1→L+2 (55%), H→L+5 (15%)
28	298.1	0.0435	H-3→L+1 (41%), H-2→L+1 (30%), H-1→L+2 (11%)
29	296.7	0.0881	H→L+5 (54%), H→L+6 (13%), H-1→L+2 (11%)
31	293.8	0.0402	H-19→L (49%), H-20→L (23%)
32	290.8	0.1130	H→L+7 (60%), H→L+6 (14%)
37	282.7	0.1260	H→L+8 (73%)
40	277.9	0.0584	H-1→L+3 (21%), H-2→L+3 (18%), H-5→L+1 (12%), H-2→L+2 (11%)
41	276.8	0.1300	H-5→L+1 (66%)
46	272.0	0.0738	H-6→L+1 (34%), H-1→L+7 (27%)
52	264.8	0.0472	H-4→L+4 (31%), H-4→L+2 (19%), H→L+12 (11%)
53	263.5	0.0603	H-6→L+1 (28%), H-1→L+7 (22%)
55	262.4	0.0544	H→L+12 (57%)

56	261.7	0.0416	H-2→L+6 (34%), H-1→L+6 (20%)
58	260.2	0.0514	H-4→L+2 (24%), H-1→L+8 (20%), H-23→L (12%), H-4→L+4 (10%)
59	259.6	0.0542	H-23→L (42%)

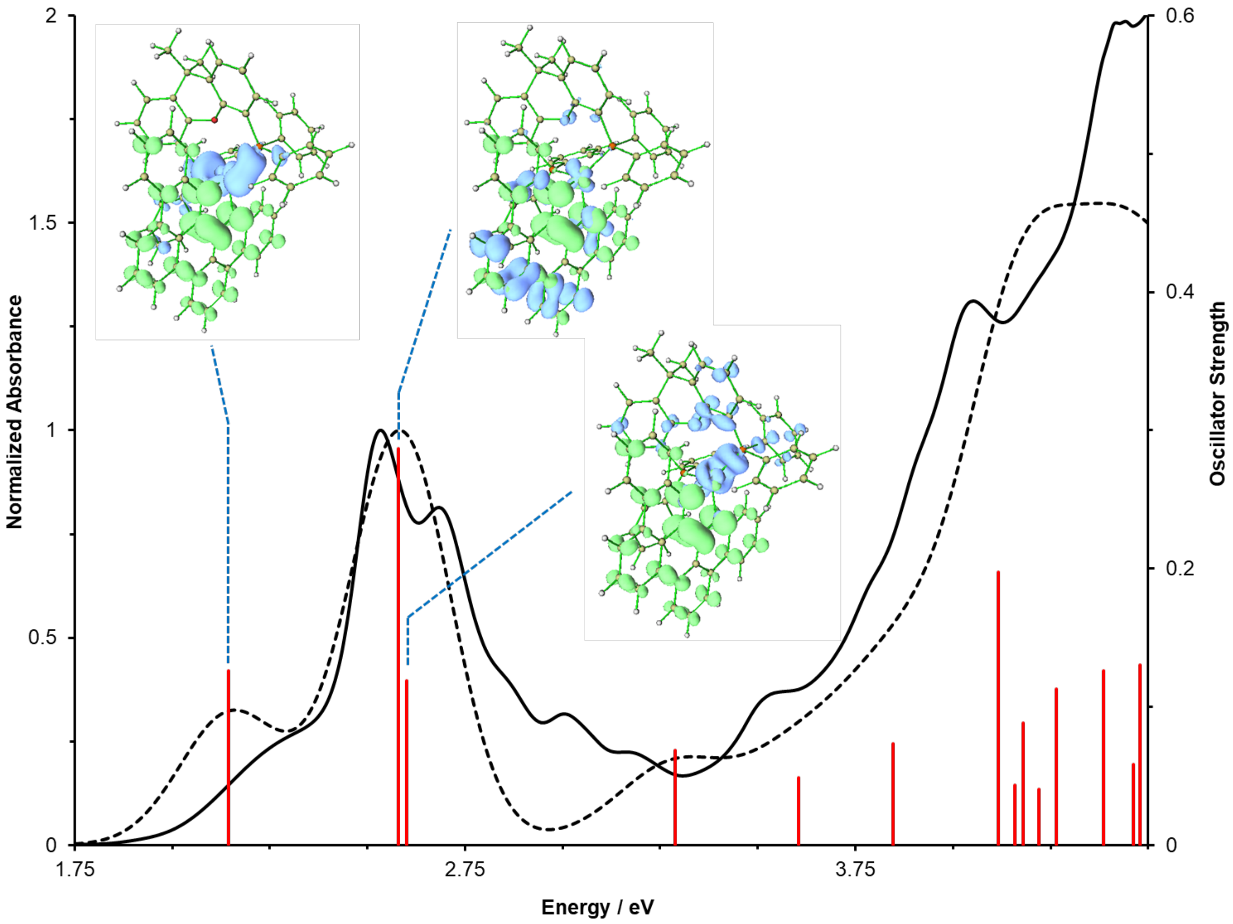

Figure S10. Comparison of the experimental (-) with the computed scalar-only (---) spectra, and vertical energy transitions (red solid line) for [(P^P)Cu(*p*-biphe)]$^{+}$ in dichloromethane (FWHM = 0.35 eV; RIJCOSX-ZORA-SMD-PBE0/ZORA-def2TZVP+SARC/J//SMD-O3LYP-D4/def2-SVP). Only transitions with oscillator strengths > 0.04 are shown. Electron-hole density maps shown for three major lowest energy transitions.

Table S7. TD-DFT calculated electronic transitions along with their corresponding excitation energies and oscillator strengths ($f_{OSC} > 0.04$) for $[Cu(p\text{-biphe})_2]^+$ (contributions > 10%). Only the first 75 transitions were considered.

No.	E / nm	f_{OSC}	Composition
**1**	**744.7**	**0.0431**	**HOMO→LUMO (78%), HOMO-1→LUMO+1 (20%)**
**3**	**606.1**	**0.3561**	**HOMO-1→LUMO+1 (77%), HOMO→LUMO (20%)**
4	507.7	0.2561	H-2→L (75%)
5	507.4	0.1883	H-2→L+1 (78%)
8	473.9	0.1560	H-3→L (80%)
10	462.8	0.2300	H-3→L+1 (62%), H-6→L+1 (20%), H-5→L+1 (13%)
15	383.2	0.0457	H-5→L (47%), H-6→L (28%), H-9→L+1 (13%)
16	380.1	0.0717	H-5→L+1 (46%), H-6→L+1 (25%)
17	373.2	0.0922	H-7→L (55%), H-8→L+1 (36%)
25	338.8	0.0761	H-11→L+1 (47%), H-14→L+1 (33%)
26	338.4	0.1052	H-11→L+1 (42%), H-14→L (37%)
31	323.1	0.0825	H-2→L+2 (60%), H-3→L+3 (20%)
32	322.8	0.0500	H-2→L+3 (55%), H-3→L+2 (29%)
34	319.7	0.1058	H-12→L (52%), H-13→L+1 (39%)
36	308.9	0.0599	H-1→L+5 (95%)
45	299.8	0.8065	H-2→L+4 (55%), H-3→L+5 (25%)
55	277.6	0.0889	H-5→L+2 (41%), H-4→L+3 (33%), H-4→L+2 (12%)
56	277.6	0.1247	H-4→L+2 (48%), H-5→L+3 (28%)
61	268.1	0.1242	H-2→L+9 (32%), H-3→L+8 (26%), H-7→L+2 (15%), H-8→L+3 (12%)

67	261.9	0.2438	H-16→L (49%), H-17→L+1 (31%)
75	257.7	0.2933	H-7→L+2 (44%), H-8→L+3 (20%), H-2→L+9 (16%)

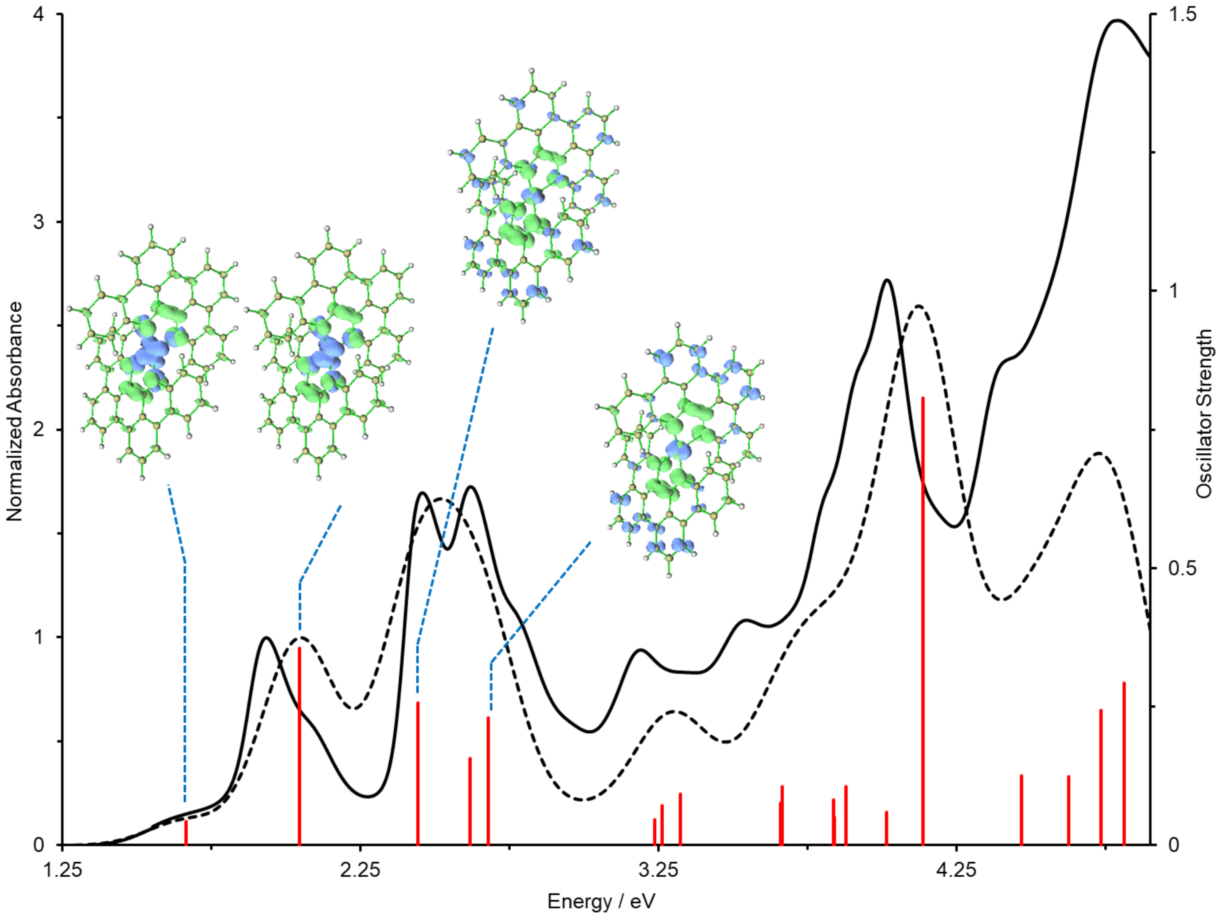

Figure S11. Comparison of the experimental (-) with the computed scalar-only (---) spectra, and vertical energy transitions (red solid line) for $[Cu(p\text{-biphe})_2]^+$ in dichloromethane (FWHM = 0.35 eV; RIJCOSX-ZORA-SMD-PBE0/ZORA-def2TZVP+SARC/J//SMD-O3LYP-D4/def2-SVP). Only transitions with oscillator strengths > 0.04 are shown and electron hole density maps are shown for four of the major low energy transitions.

Table S8. TD-DFT calculated electronic transitions along with their corresponding excitation energies and oscillator strengths (f_{OSC} > 0.04; f_{OSC} > 0.01 at lower energy than 500 nm) for $[Ru(bpy)_2(biphe)][PF_6]_2$ (contributions > 10%). Only the first 50 transitions were considered.

No.	E / nm	f_{OSC}	Composition
1	807.7	0.0117	H→L (94%)
3	582.4	0.2340	H-1→L (60%), H-2→L (32%)
4	536.1	0.0211	H→L+1 (59%), H→L+2 (28%)
5	532.9	0.0122	H→L+2 (53%), H→L+1 (34%)
6	524.5	0.1808	H-3→L (76%), H→L+2 (16%)
9	451.2	0.0926	H-2→L+2 (45%), H-1→L+1 (39%)
10	432.5	0.0854	H-4→L (83%)
19	378.9	0.0743	H→L+5 (72%), H→L+6 (14%)
20	374.2	0.0503	H-8→L (55%), H→L+6 (17%), H-1→L+3 (16%)
21	373.1	0.0401	H→L+6 (59%), H-8→L (13%), H-1→L+3 (10%)
22	371.7	0.0421	H-1→L+3 (59%), H-8→L (17%), H-2→L+3 (11%)
27	355.7	0.0461	H-1→L+4 (68%)
30	344.5	0.0453	H-2→L+5 (36%), H-4→+1 (31%), H-1→L+5 (23%)
31	344.0	0.0531	H-4→L+1 (59%), H-2→L+5 (21%), H-1→L+5 (11%)
47	311.7	0.0540	H-3→L+5 (25%), H-1→L+9 (24%), H→L+10 (10%)

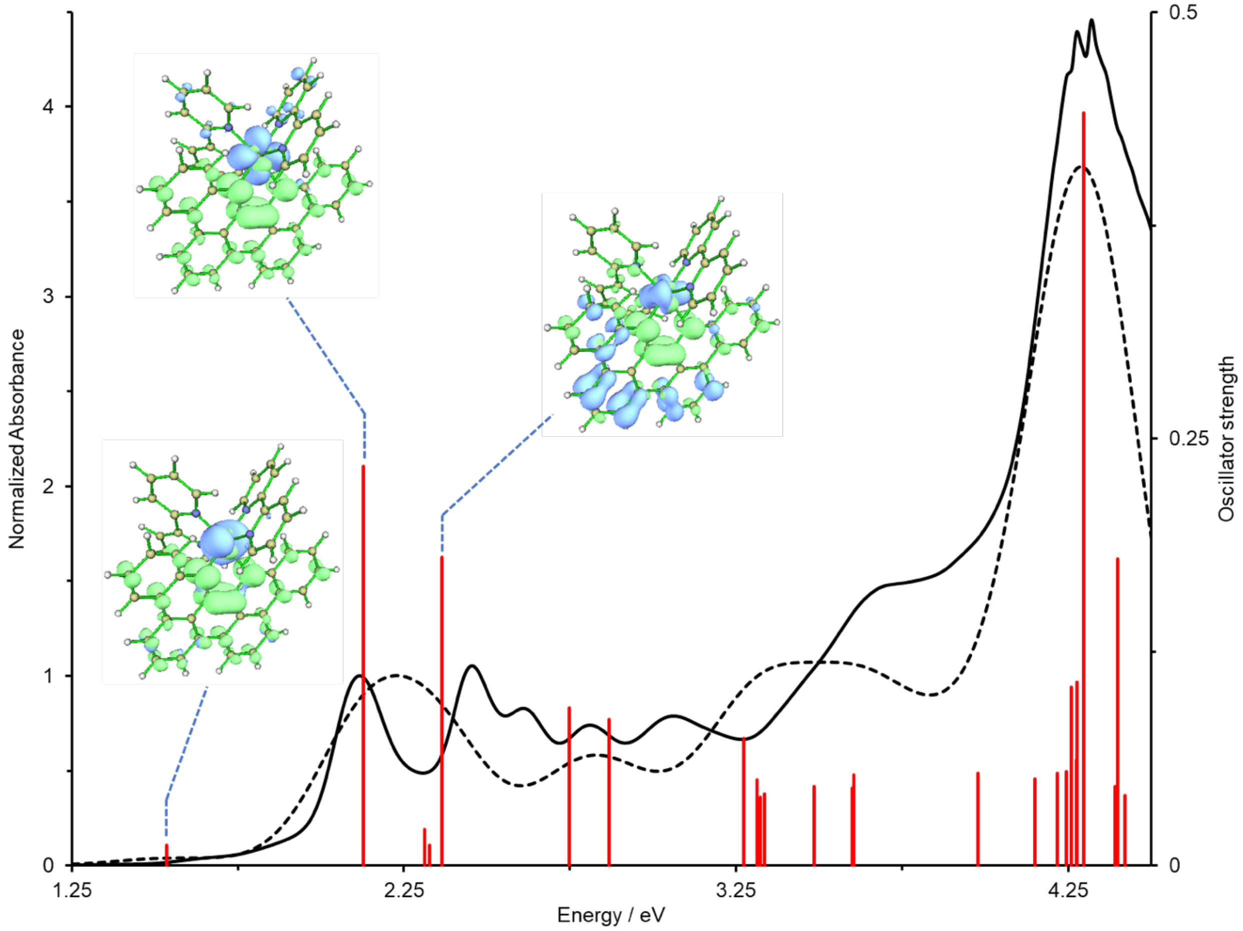

Figure S12. Comparison of the experimental (-) with the computed scalar-only (---) spectra, and vertical energy transitions (red solid line) for [Ru(bpy)$_2$(biphe)][PF$_6$]$_2$ in acetonitrile (FWHM = 0.35 eV; RIJCOSX-ZORA-SMD-O3LYP/OLD-ZORA-TZVP+SARC/J//SMD-PBE0-D3(BJ)/def2-SVP). At energies < 2.5 eV only transitions with oscillator strengths > 0.01 are shown; at energies > 2.5 eV only transitions with oscillator strengths > 0.04 are shown. Electron-hole density maps shown for three major lowest energy transitions.

Table S9. TD-DFT calculated electronic transitions along with their corresponding excitation energies and oscillator strengths ((f_{OSC} > 0.04; f_{OSC} > 0.03 at lower energy than 500 nm) for $[Ir(ppy)_2(p\text{-biphe})]PF_6$ (contributions > 10%). Only the first 50 transitions were considered.

No.	E / nm	f_{OSC}	Composition
1	619.5	0.0367	HOMO→LUMO (96%)
2	539.2	0.1870	HOMO-1→LUMO (85%), HOMO-3→LUMO (10%)
3	480.8	0.0519	HOMO-2→LUMO (82%), HOMO-3→LUMO (12%)
4	472.8	0.1170	HOMO-3→LUMO (65%), HOMO-2→LUMO (15%)
5	465.0	0.1636	HOMO-4→LUMO (83%)
10	377.1	0.0601	HOMO→LUMO+2 (60%), HOMO-8→LUMO (20%)
12	356.4	0.0995	HOMO-10→LUMO (66%), HOMO-9→LUMO (24%)
15	329.9	0.0637	HOMO-1→LUMO+2 (49%), HOMO-3→LUMO+2 (24%), HOMO-1→LUMO+1 (12%)
16	325.2	0.0448	HOMO-1→LUMO+1 (58%), HOMO-11→LUMO (13%), HOMO-1→LUMO+2 (11%)
20	319.5	0.0489	HOMO→LUMO+4 (60%), HOMO→LUMO+5 (22%)
21	316.0	0.0679	HOMO-2→LUMO+3 (57%), HOMO→LUMO+5 (11%)
22	315.5	0.0482	HOMO→LUMO+5 (46%), HOMO-2→LUMO+3 (19%), HOMO→LUMO+4 (16%)
24	311.1	0.0424	HOMO-4→LUMO+2 (55%)
25	307.4	0.1932	HOMO-1→LUMO+4 (51%), HOMO-4→LUMO+3 (20%)
26	305.6	0.1177	HOMO-4→LUMO+3 (41%), HOMO→LUMO+6 (24%)
32	291.9	0.0438	HOMO-3→LUMO+1 (46%)
33	289.8	0.0574	N/A [a]

35	287.5	0.1003	HOMO-13→LUMO (21%), HOMO-4→LUMO+1 (12%), HOMO-5→LUMO+2 (12%)
36	286.8	0.0444	HOMO-13→LUMO (32%), HOMO-5→LUMO+2 (16%), HOMO→LUMO+7 (15%)
37	285.9	0.1099	HOMO→LUMO+7 (52%)
38	282.5	0.0467	HOMO-1→LUMO+7 (22%), HOMO-1→LUMO+5 (18%), HOMO-3→LUMO+5 (14%), HOMO-1→LUMO+6 (13%)
44	275.4	0.0831	HOMO-2→LUMO+5 (29%), HOMO-5→LUMO+3 (18%), HOMO-4→LUMO+4 (12%)
45	274.6	0.0996	HOMO-4→LUMO+4 (29%), HOMO-6→LUMO+2 (13%), HOMO-5→LUMO+3 (13%)
46	274.5	0.1289	HOMO-14→LUMO (31%), HOMO-6→LUMO+2 (16%), HOMO-3→LUMO+4 (12%)
48	273.1	0.0448	HOMO-1→LUMO+6 (26%), HOMO-3→LUMO+4 (15%), HOMO-14→LUMO (10%), HOMO-1→LUMO+7 (10)%

[a] No contributions above 10%

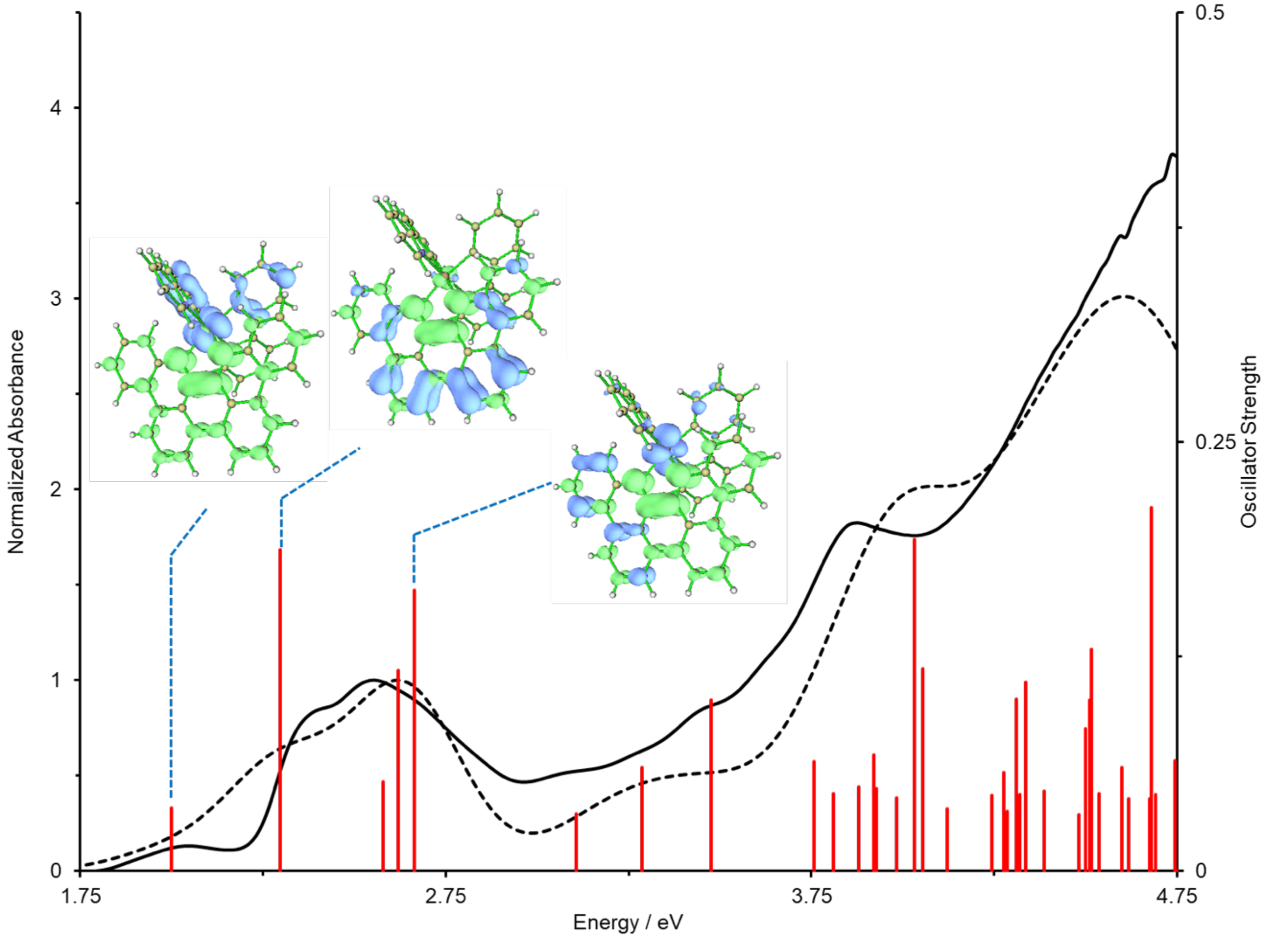

Figure S13. Comparison of the experimental (-) with the computed scalar-only (---) spectra, and vertical energy transitions (red solid line) for $[Ir(ppy)_2(p\text{-biphe})]PF_6$ in acetonitrile (FWHM = 0.35 eV; RIJCOSX-ZORA-SMD-M06/OLD-ZORA-TZVP+SARC/J//SMD-PBE0-D3(BJ)/def2-SVP). Only transitions with oscillator strengths > 0.03 are shown and electron hole density maps (isosurface = 0.002) shown for the three major lowest energy transitions.

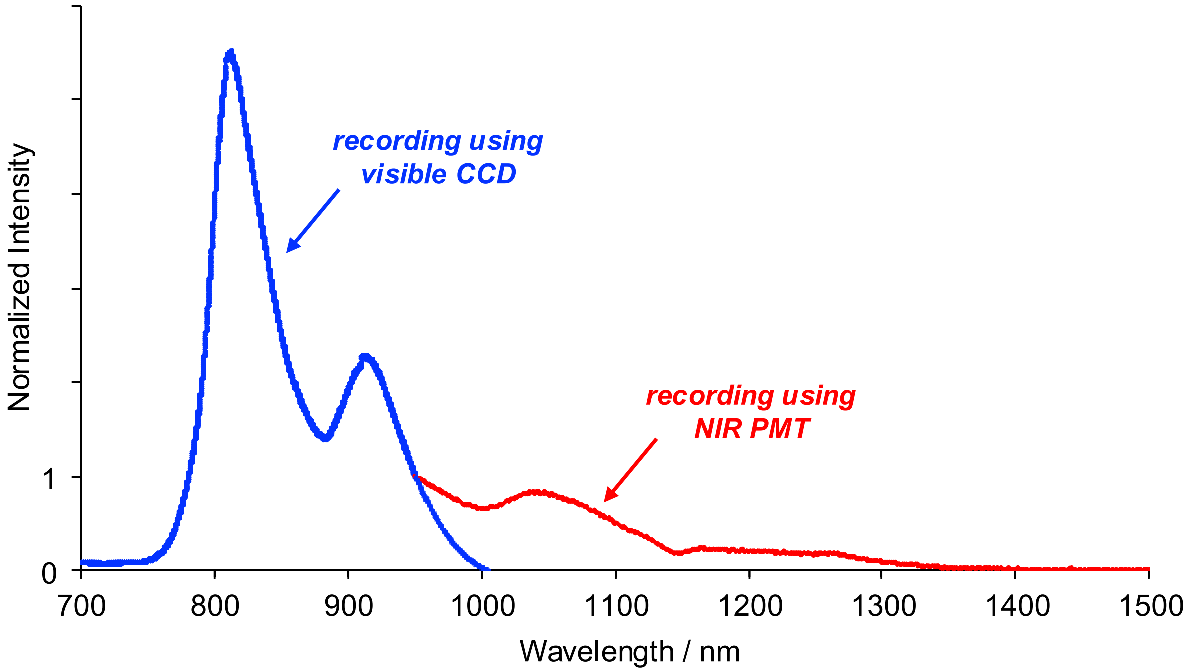

Figure S14. Emission spectra of [Ir(ppy)$_2$(*p*-biphe)]PF$_6$ recorded in in CH_2Cl_2 at 295 K using a CCD detector sensitive to the visible range of the electromagnetic spectrum (blue) and a NIR sensitive PMT detector (red). The 3rd and 4th vibrational shoulders are evident > 1000 nm.

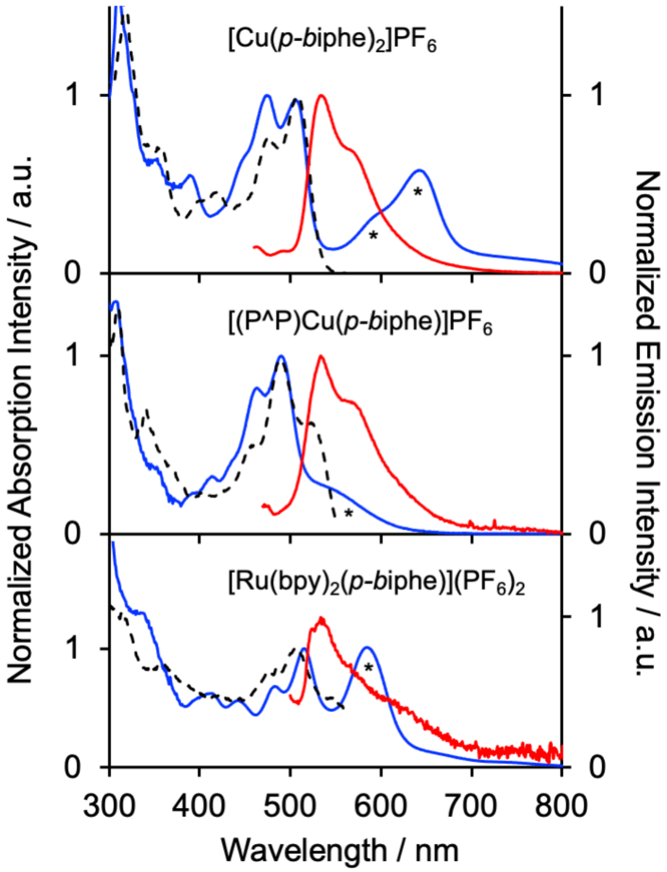

Figure S15. Spectra of the weak fluorescence displayed by the Cu(I) complexes and the Ru(II) complex when excited at higher energies (*i.e.*, *not* into the low-energy absorption band assigned as MLCT), at 295 K in CH_2Cl_2 (Cu) or MeCN (Ru) respectively (red lines). The corresponding excitation spectra registered at λ_{em} = 580 nm are shown as dashed black lines, and the absorption spectra are in blue where asterisks mark the low-energy bands that are missing from the excitation spectra. The emission is tentatively attributed to *p*-biphe-based ligand-centred states.

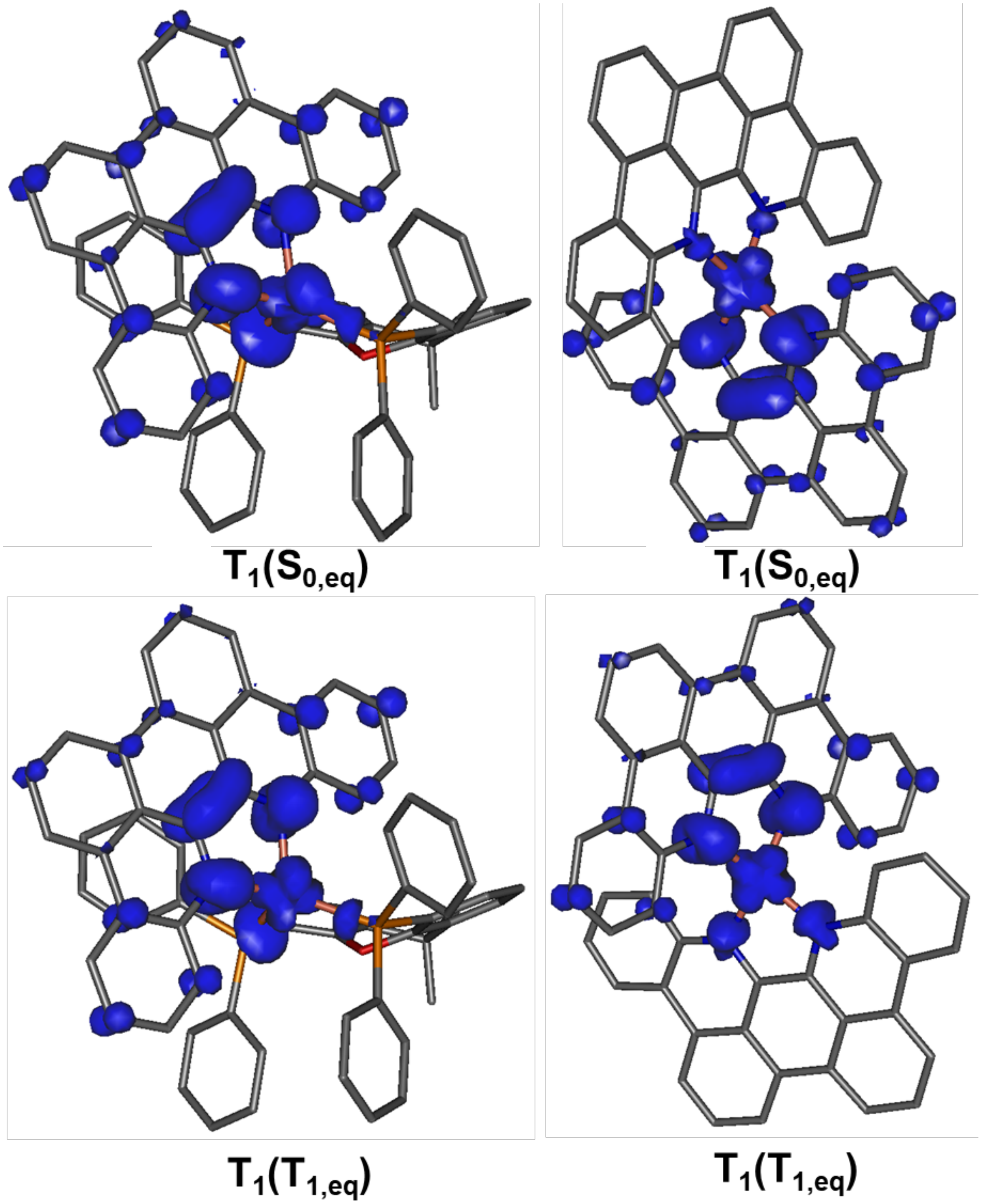

Figure S16. Spin density plot of [(P^P)Cu(*p*-biphe)]$^+$ (right) and [Cu(*p*-biphe)$_2$]$^+$ (left).

Isosurface value = 0.004; RIJCOSX-ZORA-SMD-PBE0/ZORA-def2TZVP+SARC/J//SMD-O3LYP-D4/def2-SVP

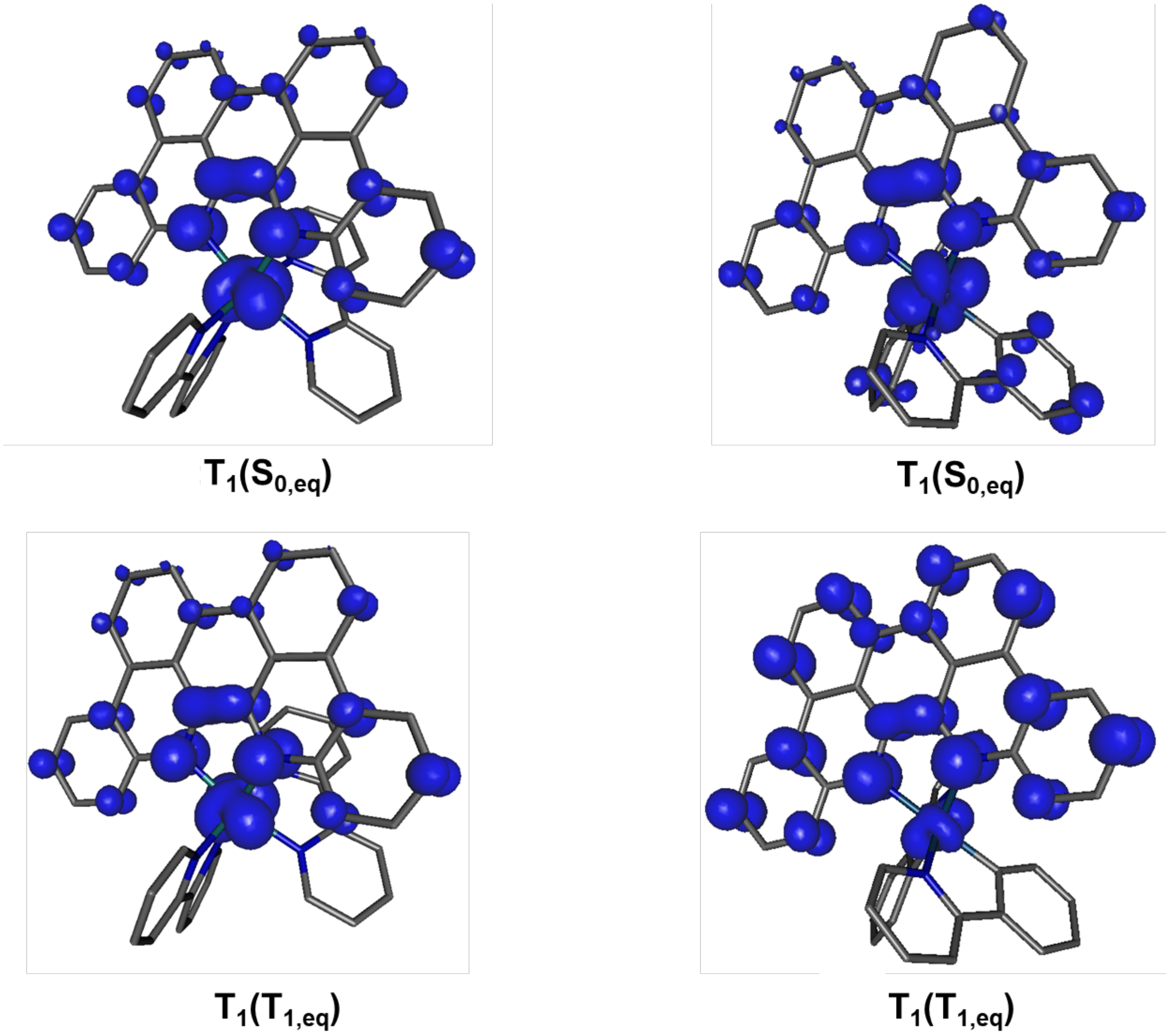

Figure S17. Spin density plot of $[Ru(bpy)_2(p\text{-biphe})]^{2+}$ (left) and $[Ir(ppy)_2(p\text{-biphe})]^{+}$ (right).

Isosurface value = 0.004

$[Ru(bpy)_2(p\text{-biphe})]^{2+}$ = RIJCOSX-ZORA-SMD-O3LYP/OLD-ZORA-TZVP+SARC/J//SMD-PBE0-D3(BJ)/def2-SVP

$[Ir(ppy)_2(p\text{-biphe})]^{+}$ = RIJCOSX-ZORA-SMD-M06/OLD-ZORA-TZVP+SARC/J//SMD-PBE0-D3(BJ)/def2-SVP).

Table S10. Metal Mulliken populations in the spin-density plots shown in Figures S14 and S15.

Complex	At the S_0 Geometry	At the T_1 Geometry
$[Cu(p\text{-biphe})_2]^+$	0.713	0.658
$[(P^\wedge P)Cu(p\text{-biphe})]^+$	0.532	0.553
$[Ru(bpy)_2(p\text{-biphe})]^{2+}$	0.726	0.726
$[Ir(ppy)_2(p\text{-biphe})]^+$	0.583	0.112

Table S11. Calculated photophysical parameters from single point calculations in eV.

Compound	$E_{adiabatic}$ (T_1-S_0)	$E_{vert,phos}$ (T_1-T_1@S_0)[c]	λ_T (T_1@S_0-S_0)[d]
**$[(P^\wedge P)Cu(p\text{-biphe})]^+$** [a]	1.31	0.93	0.38
**$[Cu(p\text{-biphe})_2]^+$** [a]	0.91	0.37	0.55
**$[Ru(bpy)_2(p\text{-biphe})]^{2+}$** [b]	1.23	1.08	0.15
**$[Ir(ppy)_2(p\text{-biphe})]^+$** [c]	1.54	1.25	0.29

[a] RIJCOSX-ZORA-SMD-PBE0/ZORA-def2TZVP+SARC/J//SMD-O3LYP-D4/def2-SVP
[b] RIJCOSX-ZORA-SMD-O3LYP/OLD-ZORA-TZVP+SARC/J//SMD-PBE0-D3(BJ)/def2-SVP
[c] RIJCOSX-ZORA-SMD-M06/OLD-ZORA-TZVP+SARC/J//SMD-PBE0-D3(BJ)/def2-SVP
[d] T_1@S_0 Energy from S_0 single point calculation at the optimized T_1 geometry

Table S12. DFT (SMD-PBE0-D3(BJ)/def2-SVP) optimized ground state and lowest energy excited triplet state bond lengths (Å) and angles (°).

**Bond/Å**	**[(P^P)Cu(*p*-biphe)]$^+$**			**[Cu(*p*-biphe)$_2$]$^+$**		
	**S_0**	**T_1**	**T_1-S_0**	**S_0**	**T_1**	**T_1-S_0**
Cu-N_1	2.053	1.962	-0.091	2.003	1.982	-0.021
Cu-N_2	2.054	2.036	-0.018	2.007	1.985	-0.022
Cu-L_1 [a]	2.237	2.308	0.071	2.006	1.976	-0.03
Cu-L_2 [a]	2.262	2.340	0.078	2.005	1.975	-0.03
C=N_1	1.314	1.343	0.029	1.316	1.327	0.011
C=N_2	1.315	1.344	0.029	1.316	1.327	0.011
C-C (Bridge)	1.454	1.418	-0.036	1.451	1.436	-0.015
**Angle/°**	**[(P^P)Cu(*p*-biphe)]$^+$**			**[Cu(*p*-biphe)$_2$]$^+$**		
	**S_0**	**T_1**	**T_1-S_0**	**S_0**	**T_1**	**T_1-S_0**
N_1-Cu-L_1/L_2	128.2	137.1	8.9	136.9	142.7	13.9
N_2-Cu-N_4/L_1	101.4	112.2	10.8	108.2	143.0	13.9
N_1-Cu-N_2	80.0	82.7	2.7	81.2	82.9	1.2
L_1-Cu-L_2	120.5	112.3	-8.2	81.1	83.2	1.5
τ_δ	0.74	0.64	0.10	0.72	0.53	0.20

[a] L_1 = P_1 and L_2 = P_2 for **[(P^P)Cu(*p*-biphe)]$^+$**; L_1 = N_3 and L_2 = N_4 for **Cu(*p*-biphe)$_2$]$^+$**.

Table S13. DFT (SMD-PBE0-D3(BJ)/def2-SVP) optimized ground state and lowest energy excited triplet state bond lengths (Å) and angles (°).

**Bond/Å**	**[Ru(bpy)$_2$(*p*-biphe)]$^{2+}$**			**[Ir(ppy)$_2$(*p*-biphe)]$^{+}$**		
	**S_0**	**T_1**	**T_1-S_0**	**S_0**	**T_1**	**T_1-S_0**
M-N_1	2.085	2.052	-0.033	2.217	2.181	-0.036
M-N_2	2.078	2.057	-0.021	2.210	2.187	-0.023
M-N_3	2.042	2.043	0.001	2.046	2.044	-0.002
M-N_4	2.050	2.069	0.019	2.064	2.066	0.002
M-L_1 [a]	2.065	2.085	0.020	2.002	2.006	0.004
M-L_2 [a]	2.070	2.076	0.006	2.008	2.014	0.006
C=N_1	1.331	1.372	0.041	1.324	1.381	0.057
C=N_2	1.327	1.361	0.034	1.320	1.373	0.053
C-C (Bridge)	1.451	1.410	-0.041	1.465	1.409	-0.056
**Angle/°**	**[Ru(bpy)$_2$(*p*-biphe)]$^{2+}$**			**[Ir(ppy)$_2$(*p*-biphe)]$^{+}$**		
	**S_0**	**T_1**	**T_1-S_0**	**S_0**	**T_1**	**T_1-S_0**
N_1-M-L_1/L_2	168.4	169.8	1.4	169.5	169.8	0.3
N_2-M-N_4/L_1	179.1	176.6	-2.5	178.8	179.3	0.5
N_3-M-L_2/N_4	173.7	175.7	2.0	174.1	174.2	0.1

[a] $L_1 = N_5$ and $L_2 = N_6$ for **[Ru(bpy)$_2$(*p*-biphe)]$^{2+}$**; $L_1 = C_1$ and $L_2 = C_2$ for **[Ir(ppy)$_2$(*p*-biphe)]$^{+}$**.

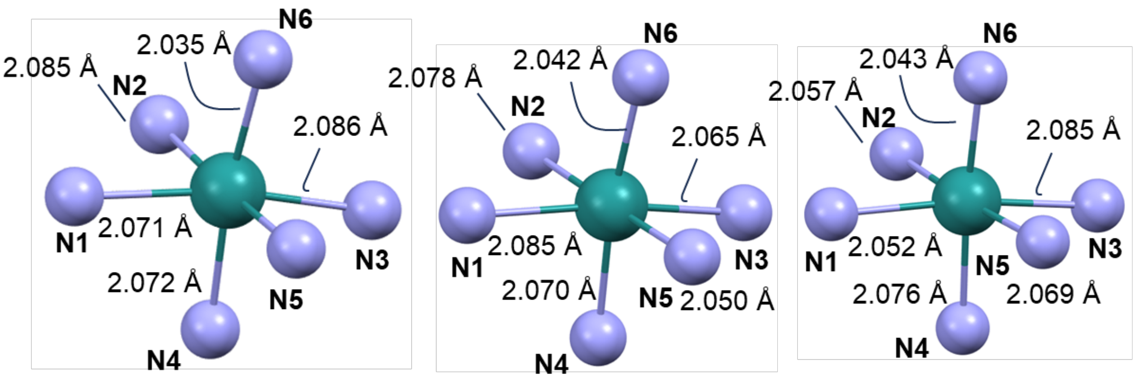

Parameter	XRD	GS (DFT)	^{3}MLCT (DFT)
d_{mean}[a] / Å	2.07	2.07	2.06
ζ[b] / Å	0.072	0.078	0.078
Δ[c]	0.00007	0.00006	0.00005
Σ[d] / °	92.0	94.7	96.0
Θ[e] / °	245	256	269

[a] Average Metal-Ligand Distance [b] $\zeta = \sum_{i=1}^{6} |d_i - d_{\mathrm{mean}}|$ [c] $\Delta = \frac{1}{6}\sum_{i=1}^{6}(\frac{d_i - d}{d})^2$
[d] $\Sigma = \sum_{i=1}^{12} |\phi_i - 90|$ [e] $\Theta = \sum_{i=1}^{24} |\theta_i - 60|$

Figure S18. Octahedricity parameters of [Ru(bpy)$_2$(*p*-biphe)]$^{2+}$ comparing XRD with the computationally determined ground state and lowest energy triplet state geometry (SMD-PBE0-D3(BJ)/def2-SVP)

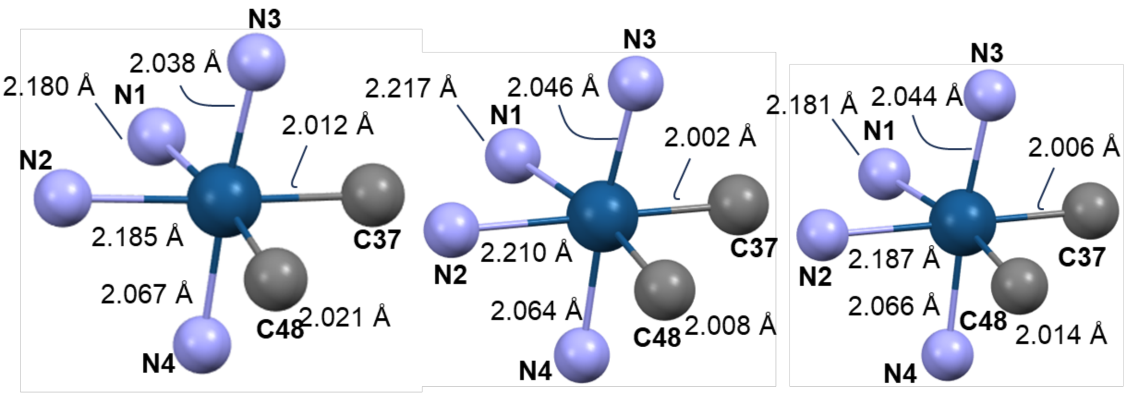

Parameter	XRD	GS (DFT)	^{3}MLCT (DFT)
d_{mean}[a] / Å	2.08	2.09	2.08
ζ[b] / Å	0.396	0.489	0.404
Δ[c]	0.00119	0.00181	0.00127
Σ[d] / °	99.1	96.5	91.7
Θ[e] / °	284	268	254

[a] Average Metal-Ligand Distance [b] $\zeta = \sum_{i=1}^{6} |d_i - d_{\text{mean}}|$ [c] $\Delta = \frac{1}{6}\sum_{i=1}^{6}(\frac{d_i - d}{d})^2$
[d] $\Sigma = \sum_{i=1}^{12} |\phi_i - 90|$ [e] $\Theta = \sum_{i=1}^{24} |\theta_i - 60|$

Figure S19. Octahedricity parameters of $[Ir(ppy)_2(p\text{-biphe})]^+$ comparing XRD with the computationally determined ground state and lowest energy triplet state geometry (SMD-PBE0-D3(BJ)/def2-SVP)

Table S14. Tabulated Cyclic Voltammetry (CV) and Spectroscopic Data[a]

**Compound**[b]	$E_{1/2(red)}$ / V	$E_{1/2(ox)}$ / V	$\Delta E_{1/2}$ / V	**Absorption λ_{max}/nm**	**Emission λ_{max}/nm at 295 K**
6,6'-biphenanthridine	-2.09, -2.39	0.93, 1.03	3.02	350	404
$[Cu(p\text{-biphe})_2]^+$	-1.23, -1.35, -1.50, -1.69 -1.92	0.51	1.74	700-850(sh)	-
$[(P^\wedge P)Cu(p\text{-biphe})]^+$	-1.26, -1.88	1.01	2.27	550(sh)	735[d] 753[d] 818[d]
$[Ru(biphe)_3]^{2+}$	-1.14, -1.27, -1.70, -1.92	1.02	2.16	549	783[d]
$[Ru(bpy)_2(biphe)]^{2+}$	-1.18, -1.59, -2.02, -2.42	0.48[c], 0.90	2.08	533	752
p-biphe	-1.23, -1.54	1.47	2.70	446	468, 492
$[Ru(bpy)_2(p\text{-biphe})]^{2+}$	-0.91, -1.44, -1.90, -2.01, -2.35	0.963	1.87	589	-
$[Ir(ppy)_2(p\text{-biphe})]^+$	-0.95, -1.53	0.948	1.90	611	810

[a] Cyclic voltammograms were performed in CH_3CN with 0.1 M $n$$Bu_4NPF_6$, GCE disk working electrode, Ag/AgCl reference electrode, Pt wire counter electrode, scan rate = 100 mV s^{-1}. All reported $E_{1/2}$ values are referenced to the $FcH^{0/+}$ (FcH = ferrocene) redox couple.

[c] Unidentified origin.

[d] at 77 K.

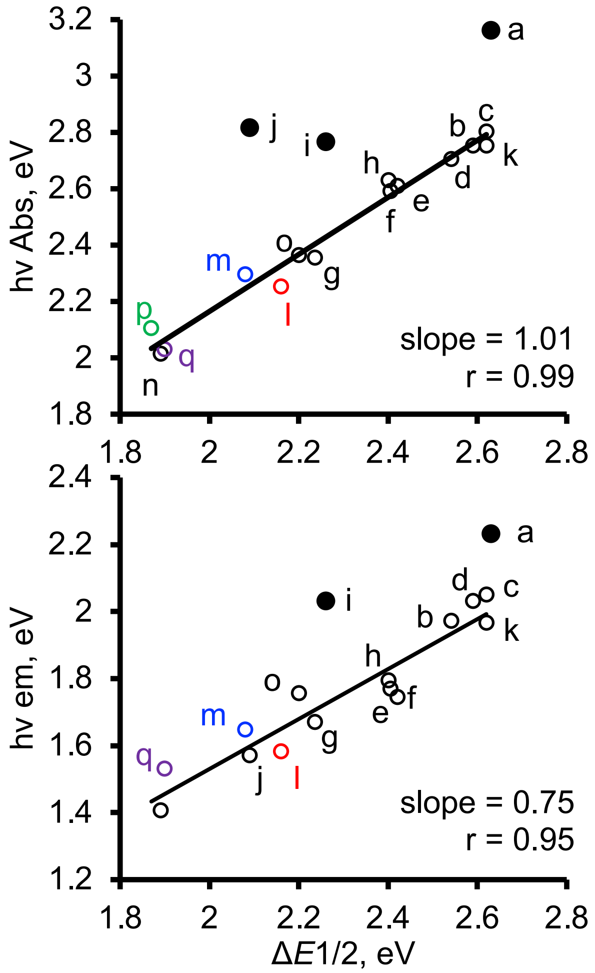

Figure S20. Correlation between (i) absorption and (ii) emission energies and redox energy $\Delta E_{1/2}$ for (a) $[Ru(3,3'\text{-biisoquinoline})_3]^{2+}$, (b) $[Ru(3,3'\text{-biisooquinoline})_2(2,2'\text{-bipyridine})]^{2+}$, (c) $[Ru(1,10\text{-phenanthroline})_3]^{2+}$, (d) $[Ru(2,2'\text{-bipyridine})_2(4,4'\text{-diphenyl-2-2'-bipyridine})]^{2+}$, (e) $[Ru(2,2'\text{-bipyridine})_2(2,2'\text{-bipyrimidine})]^{2+}$, (f) $[Ru(2,2'\text{-bipyridine})_2(2\text{-}(2\text{-pyridyl})\text{-quinoline})]^{2+}$, (g) $[Ru(2,2'\text{-bipyridine})_2(2,2'\text{-biquinoline})]^{2+}$, (h) $[Ru(2,2'\text{-bipyridine})_2(2,2'\text{-bipyrazine})]^{2+}$, (i) [Ru(2,2'-bipyridine)$_2$(dipyrido[3,2-*a*:2',3'-*c*]phenazine)]$^{2+}$, (j) [Ru(2,2'-bipyridine)$_2$(dipyrido[3,2-*c*:2',3'-*e*]pyridazine)]$^{2+}$, (k) $[Ru(2,2'\text{-bipyridine})_3]^{2+}$, (l) $[Ru(6,6'\text{-biphenanthridine})_3]^{2+}$ o, (m) $[Ru(2,2'\text{-bipyridine})_2(6,6'\text{-biphenanthridine})]^{2+}$ o, (n) [Ru(2,2'-bipyridine)$_2$(tetra-*peri*-(*tert*-butyl-benzo)-di-peri-(pyrimidino)-coronene)]$^{2+}$,[6] (o) $[Ru(3,3'\text{-biisooquinoline})_3]^{2+}$, (p) Ru(bpy)$_2$(*p*-biphe)$^{2+}$ o, and (q) Ir(ppy)$_2$(*p*-biphe)$^{+}$ o. Both the adaptation of the figure and the acquisition of the data (unless otherwise cited) was from Juris *et al.*[7] Line of best fit was generated using only open circles.

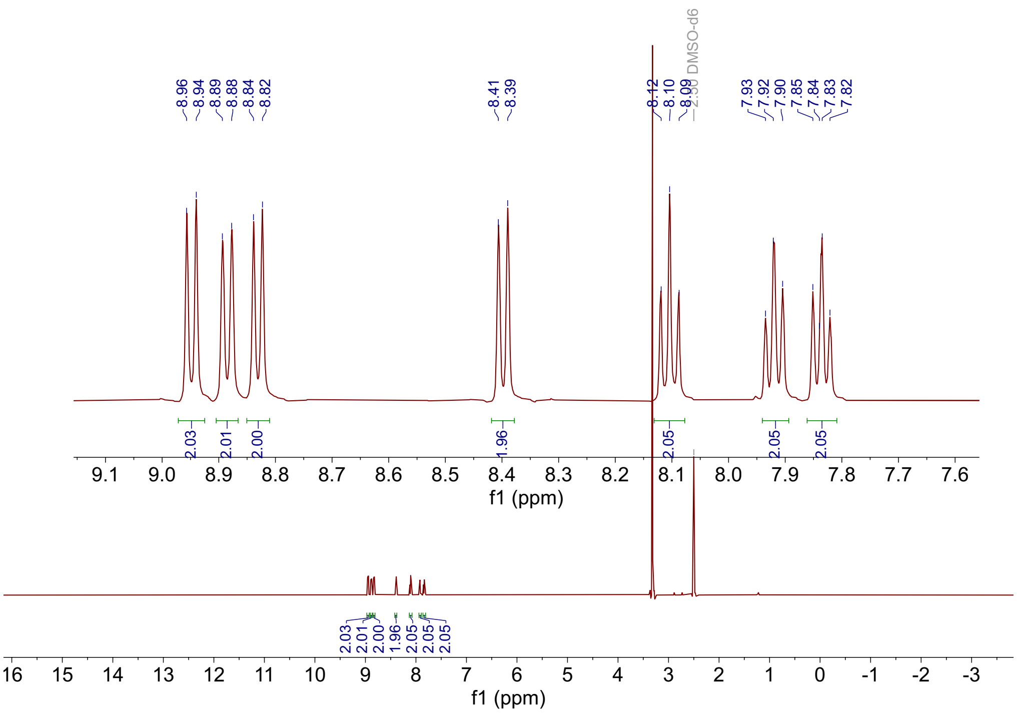

Figure S21. ^{1}H NMR (500 MHz, 22 °C, DMSO-d_6) of *p*-biphe.

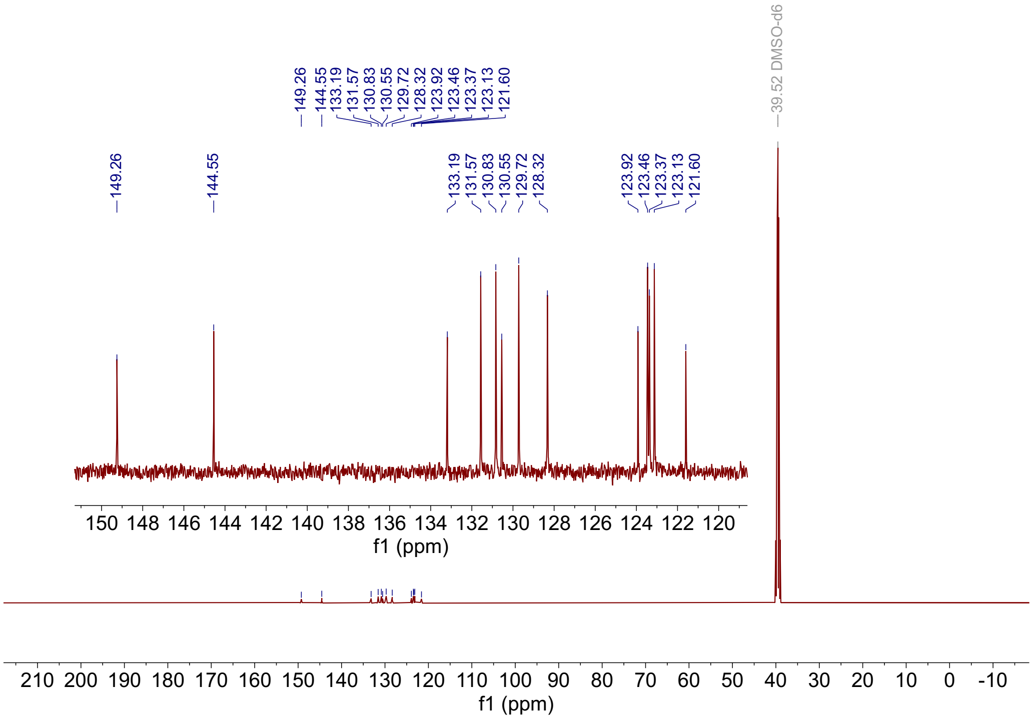

Figure S22. ^{13}C NMR (125 MHz, 22 °C, DMSO-d_6) of *p*-biphe.

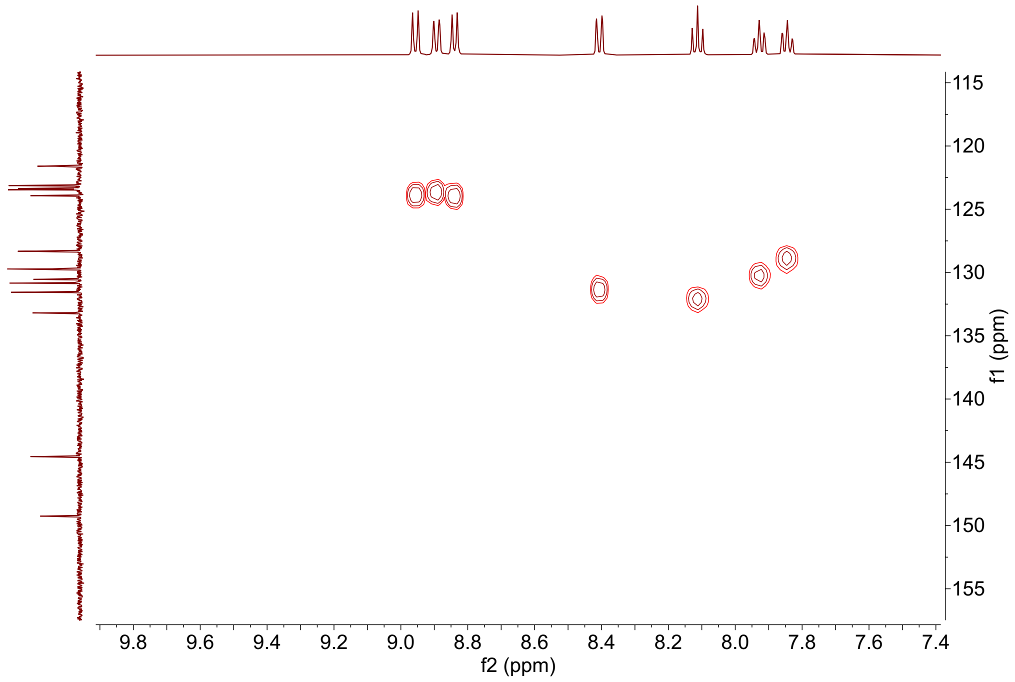

Figure S23. ^{1}H-^{13}C HSQC NMR (22 °C, DMSO-d_6) of *p*-biphe.

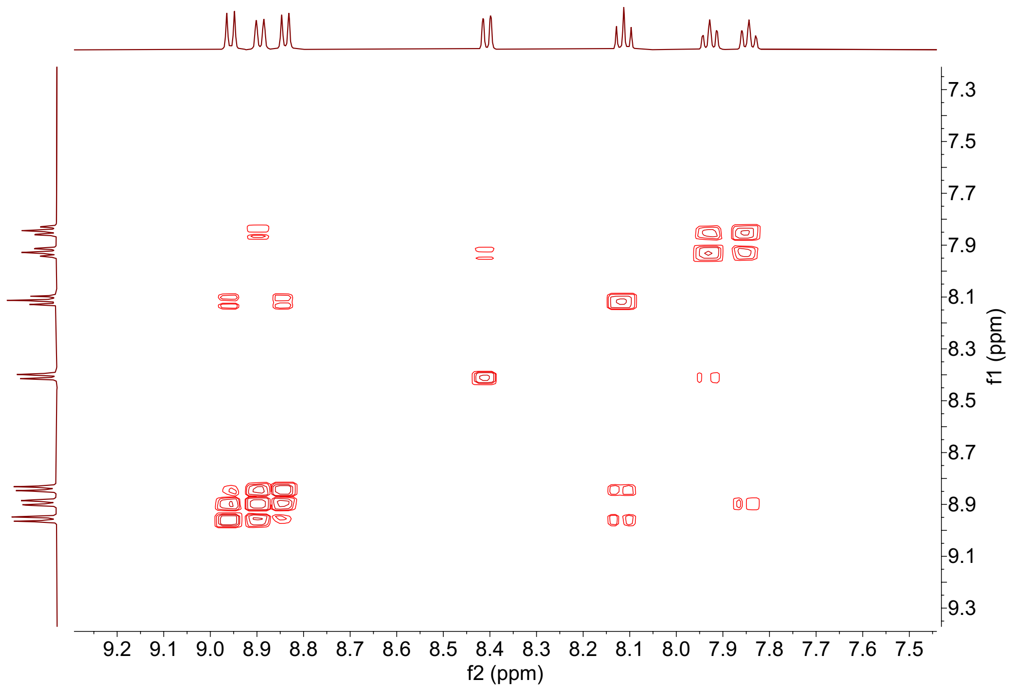

Figure S24. ^{1}H-^{1}H COSY NMR (22 °C, DMSO-d_6) of *p*-biphe.

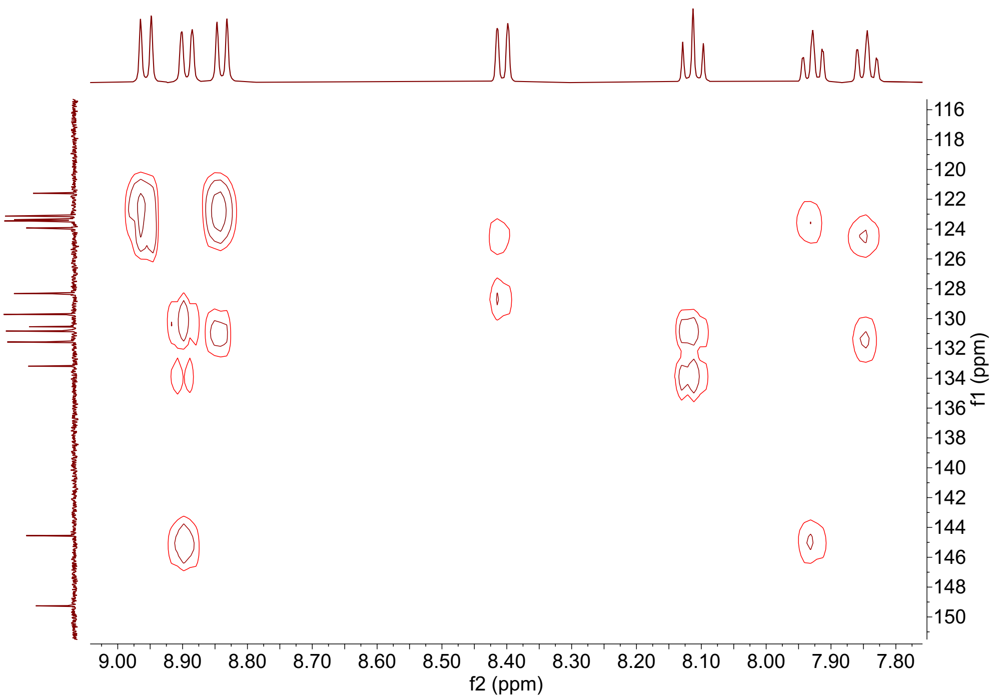

Figure S25. ^{1}H-^{13}C HMBC NMR (22 °C, DMSO-d_6) of *p*-biphe.

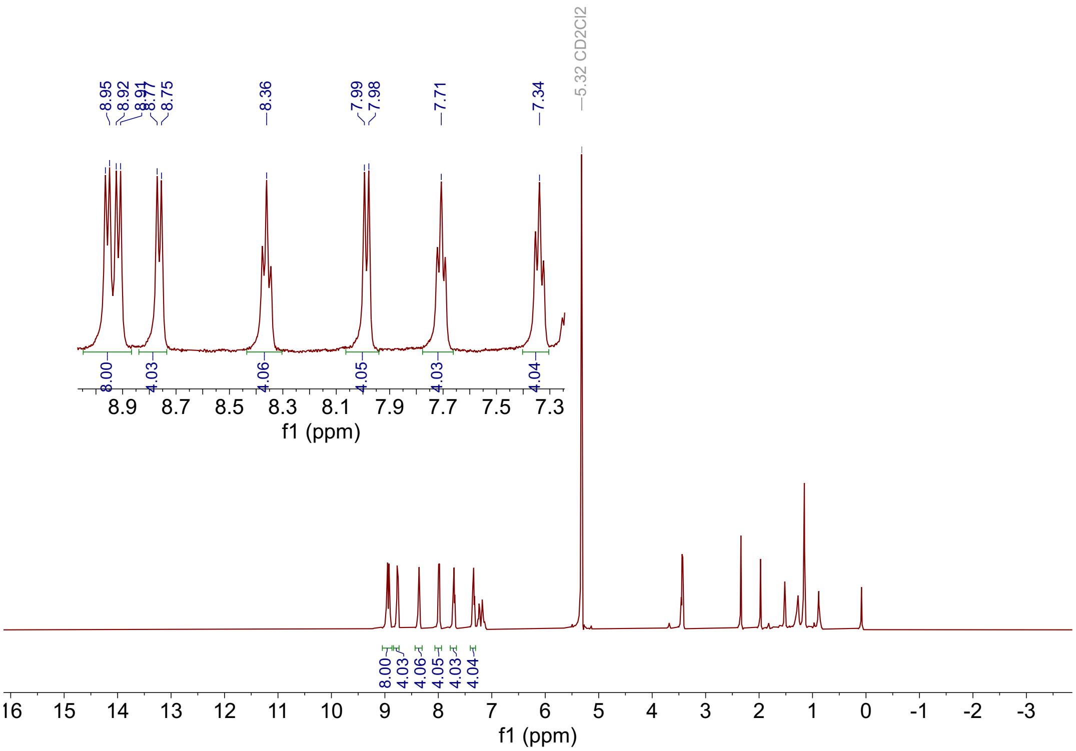

Figure S26. ^{1}H NMR (400 MHz, 22 °C, CD_2Cl_2) of $[Cu(\textit{p}\text{-biphe})_2]PF_6$.

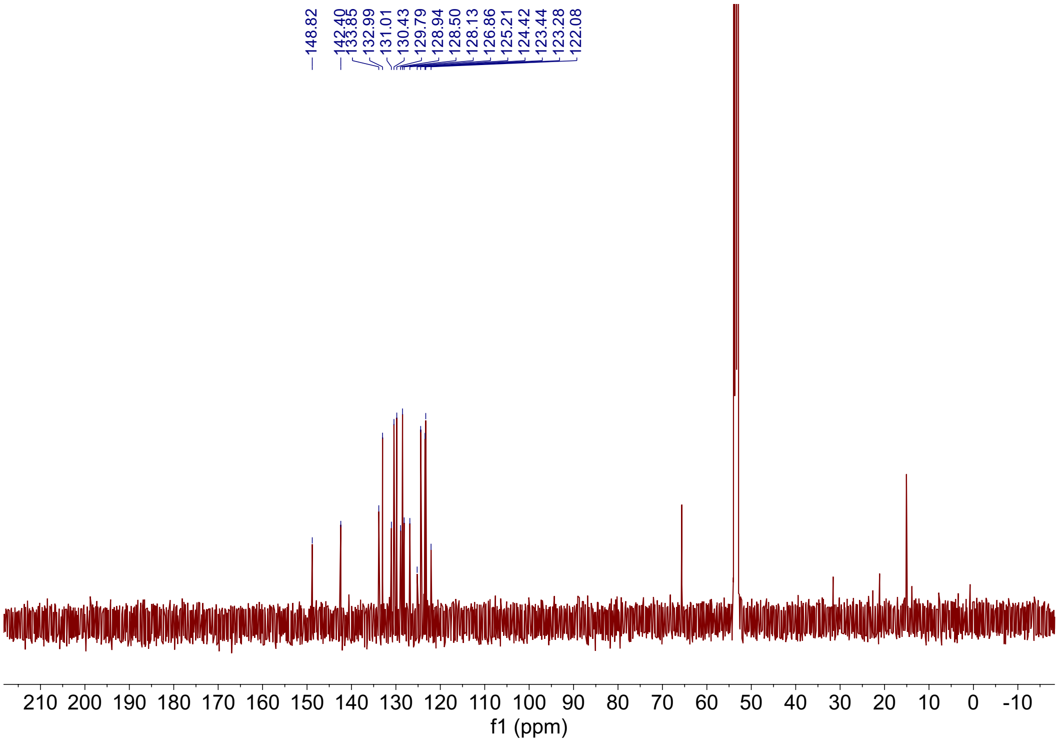

Figure S27. ^{13}C NMR (100 MHz, 22 °C, CD_2Cl_2) of [Cu(*p*-biphe)$_2$]PF_6.

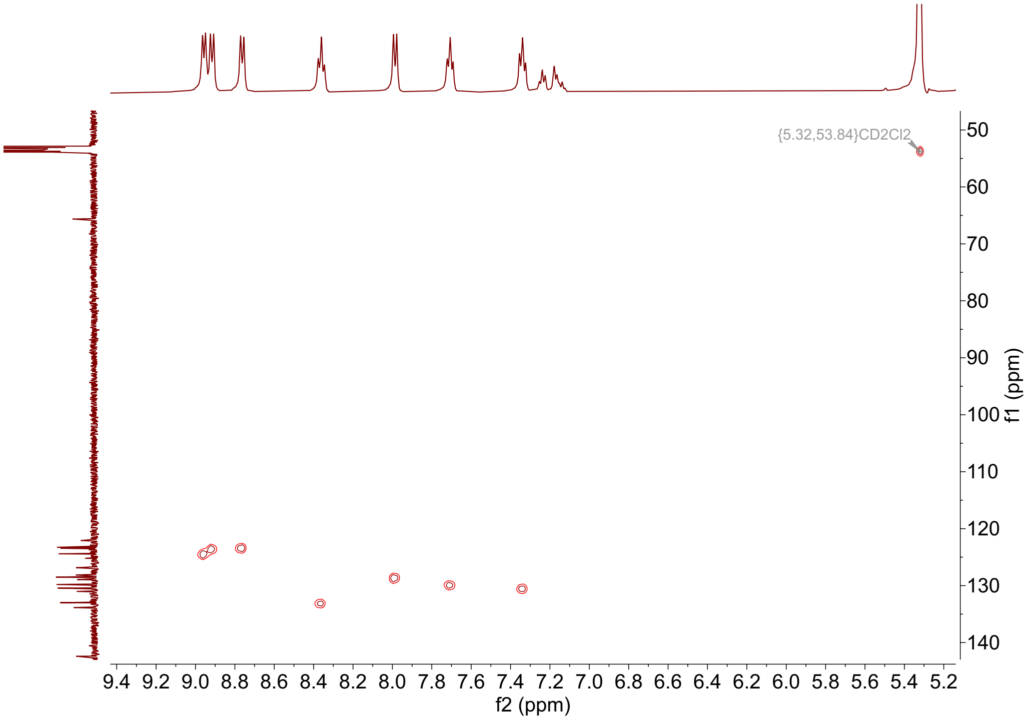

Figure S28. ^{1}H-^{13}C HSQC NMR (22 °C, CD_2Cl_2) of [Cu(*p*-biphe)$_2$]PF_6.

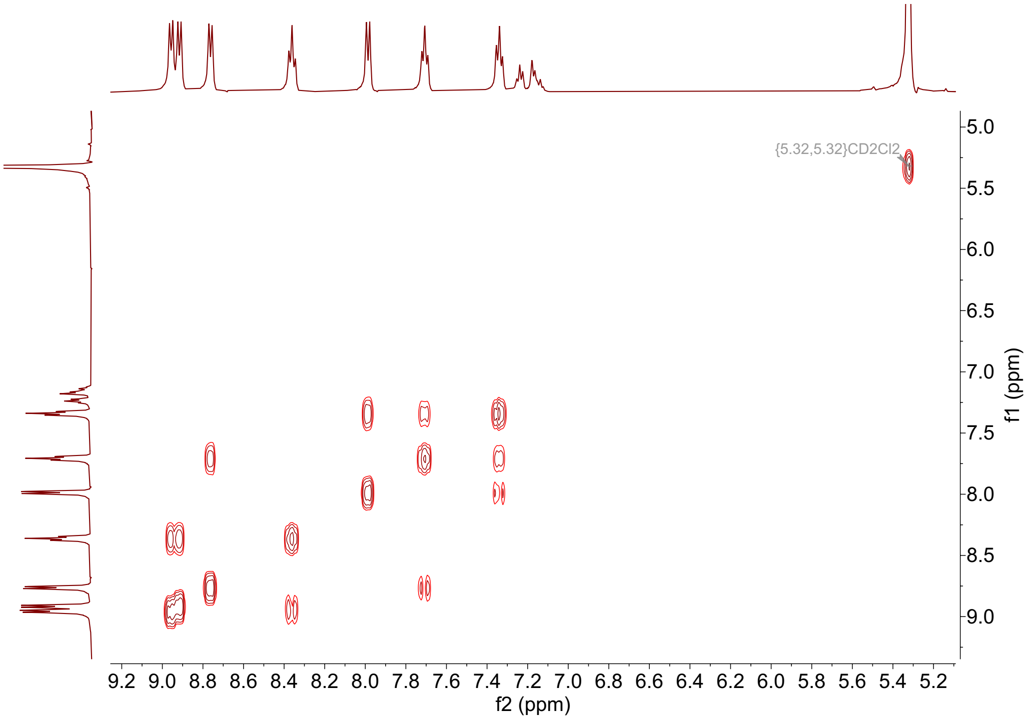

Figure S29. ^{1}H-^{1}H COSY NMR (22 °C, CD_2Cl_2) of $[Cu(\textit{p}\text{-biphe})_2]PF_6$.

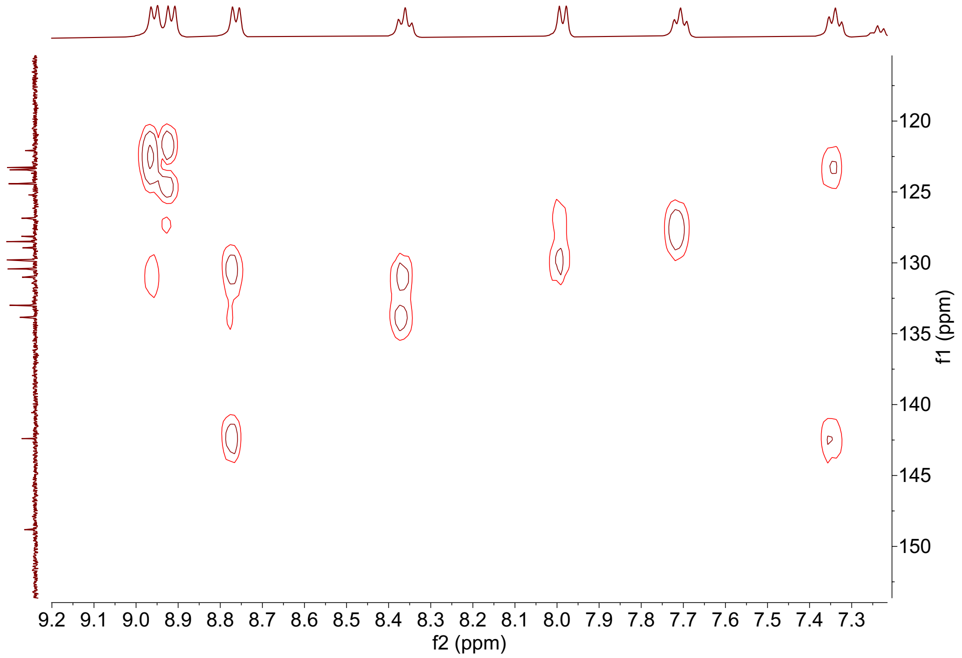

Figure S30. ^{1}H-^{13}C HMBC NMR (22 °C, CD_2Cl_2) of $[Cu(\textit{p}\text{-biphe})_2]PF_6$.

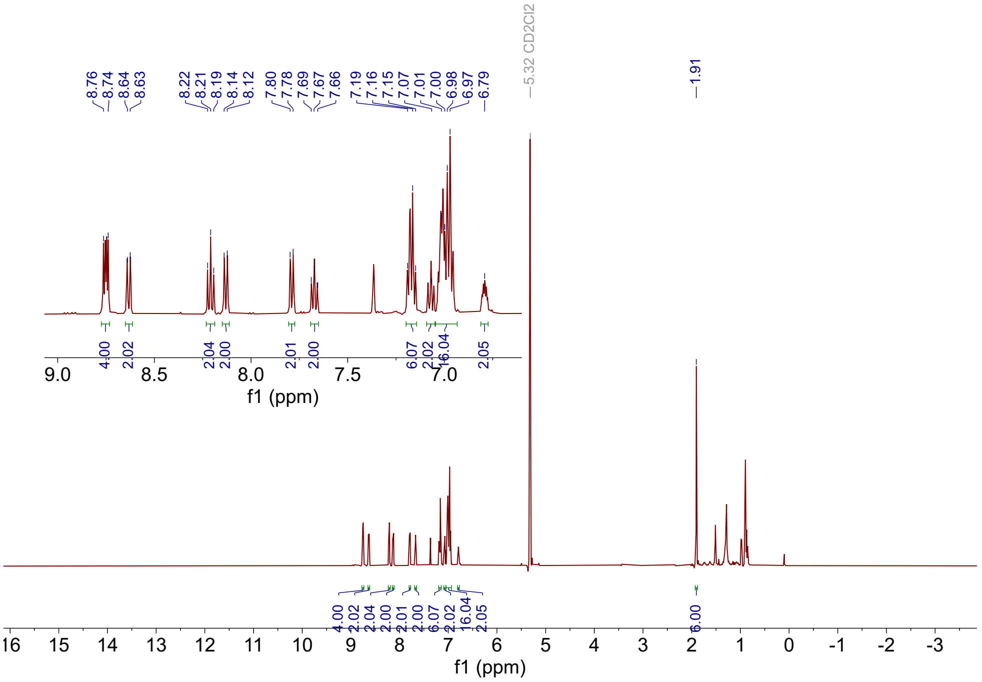

Figure S31. ^{1}H NMR (500 MHz, 22 °C, CD_2Cl_2) of [(P^P)Cu(*p*-biphe)]PF_6.

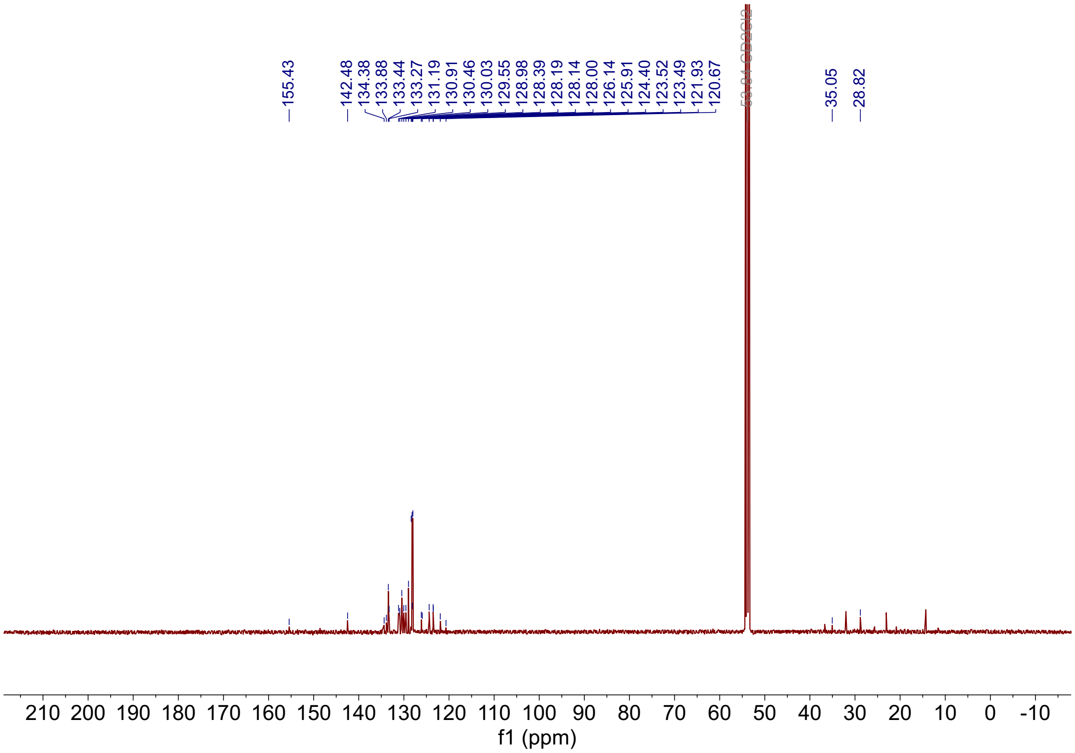

Figure S32. ^{13}C NMR (125 MHz, 22 °C, CD_2Cl_2) of [(P^P)Cu(*p*-biphe)]PF_6.

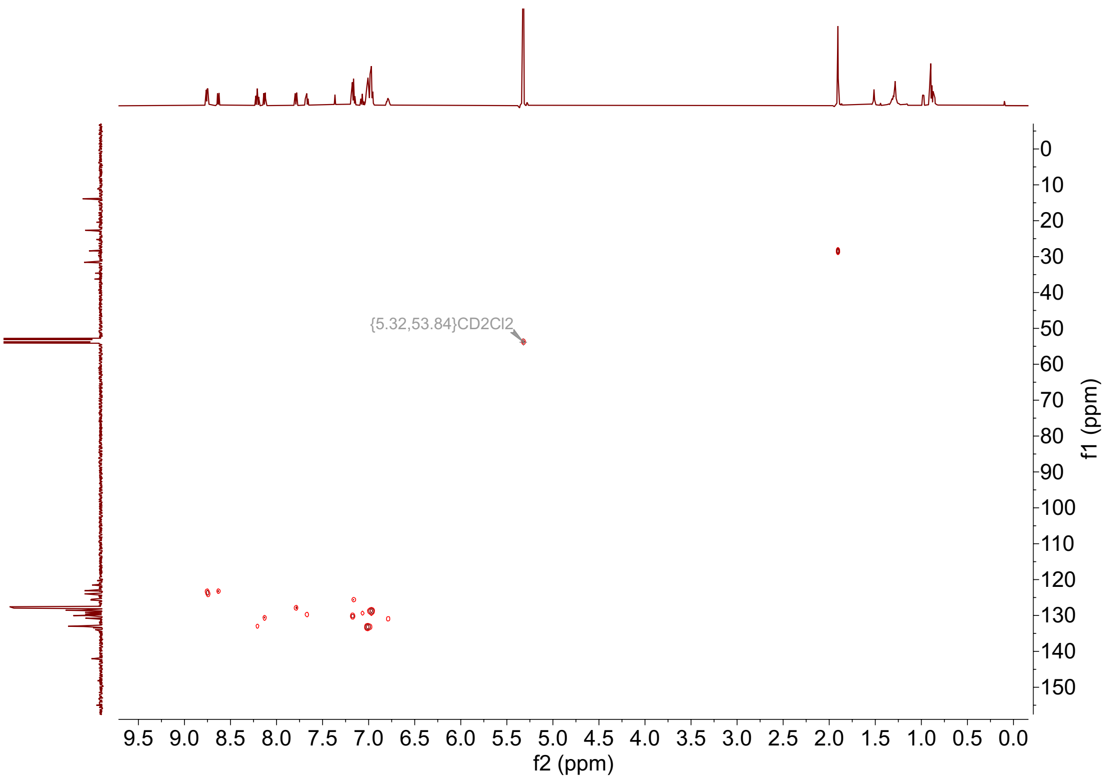

Figure S33. ^1H-^{13}C HSQC NMR (22 °C, CD_2Cl_2) of [(P^P)Cu(*p*-biphe)]PF_6.

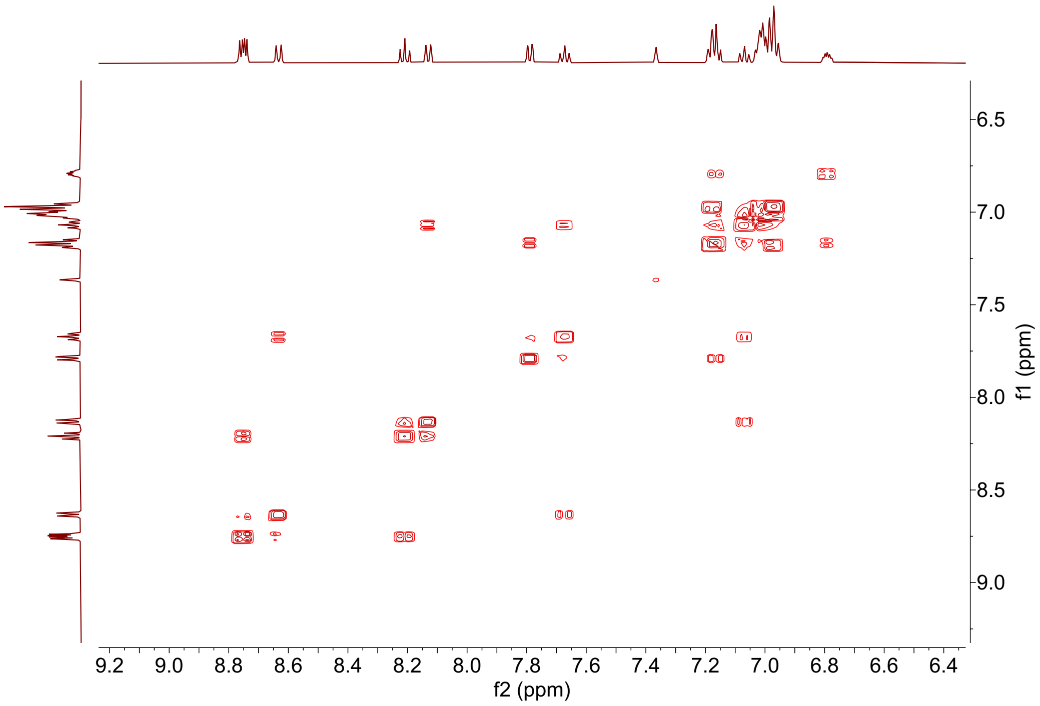

Figure S34. ^1H-^1H COSY NMR (22 °C, CD_2Cl_2) of [(P^P)Cu(*p*-biphe)]PF_6.

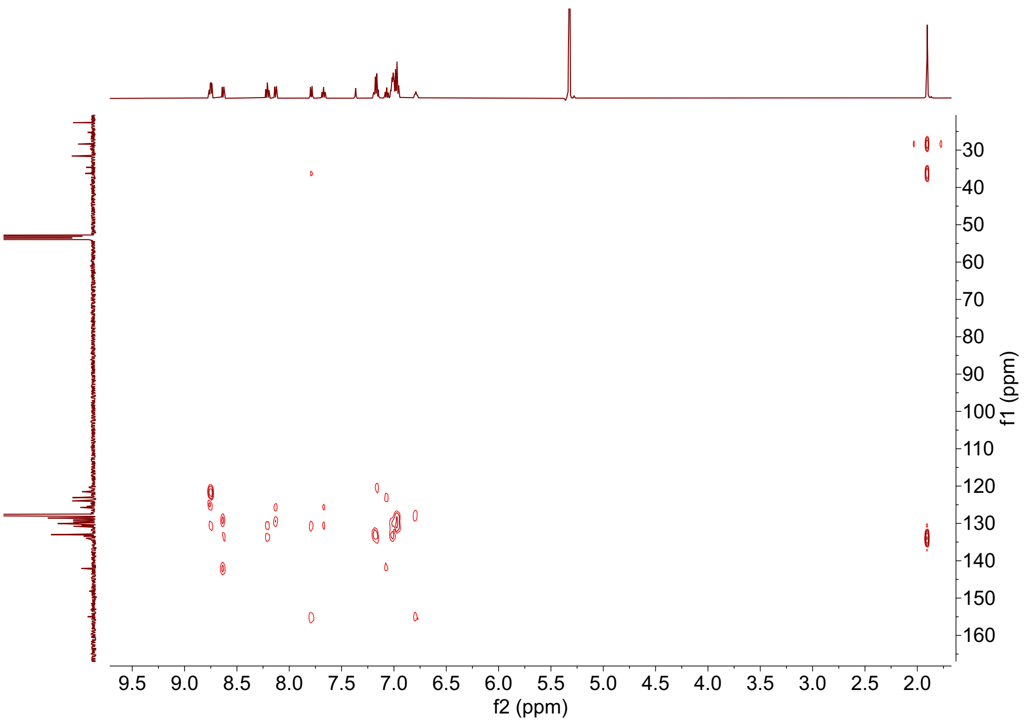

Figure S35. ^{1}H-^{13}C HMBC NMR (22 °C, CD_2Cl_2) of [(P^P)Cu(*p*-biphe)]PF_6.

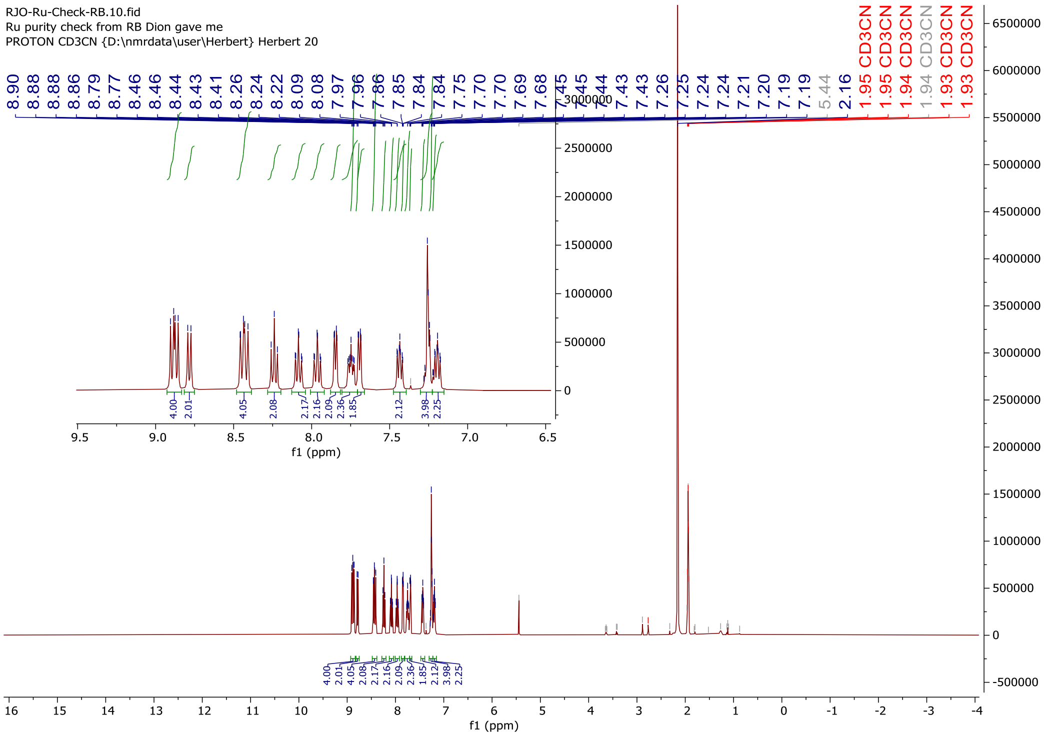

Figure S36. ^{1}H NMR (400 MHz, 22 °C, CD_3CN) of $[Ru(bpy)_2(p\text{-biphe})](PF_6)_2$.

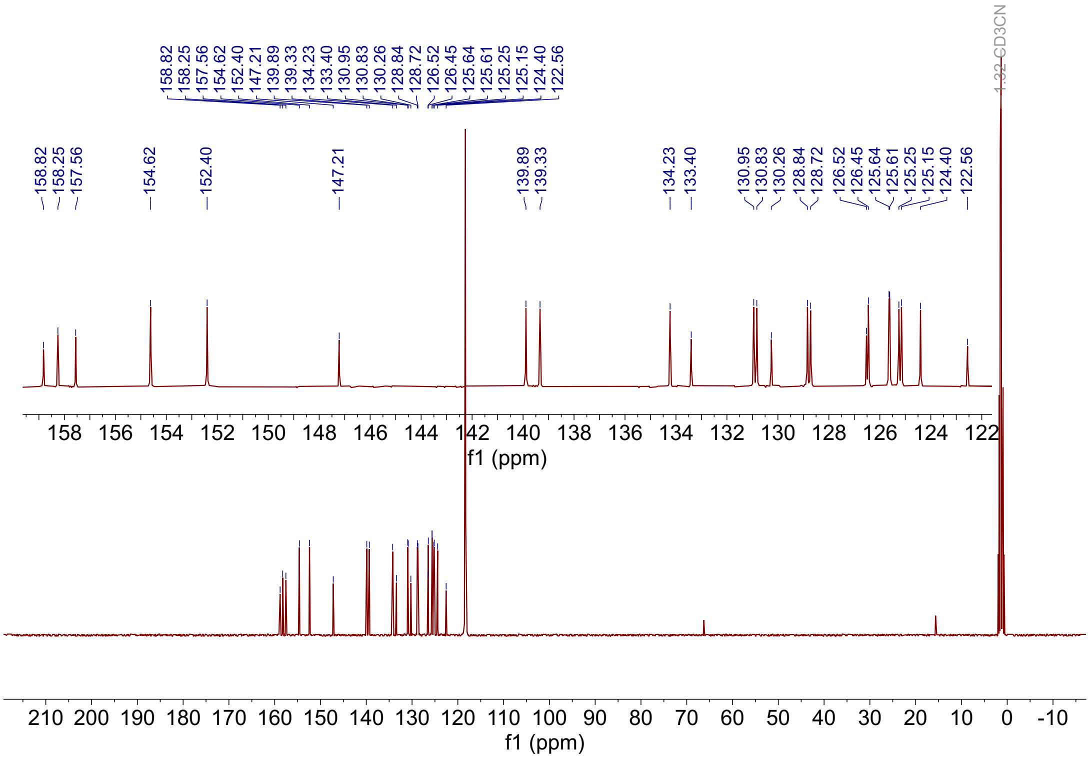

Figure S37. ^{13}C NMR (100 MHz, 22 °C, CD_3CN) of $[Ru(bpy)_2(p\text{-biphe})](PF_6)_2$.

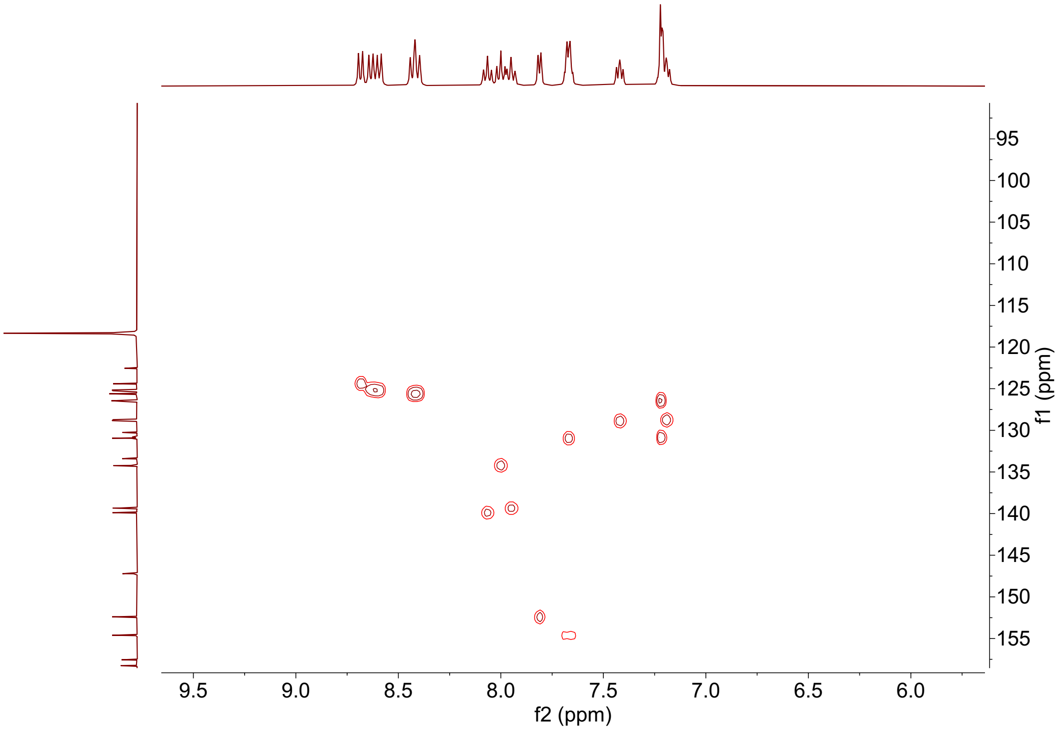

Figure S38. ^{1}H-^{13}C HSQC NMR (22 °C, CD_3CN) of $[Ru(bpy)_2(p\text{-biphe})](PF_6)_2$.

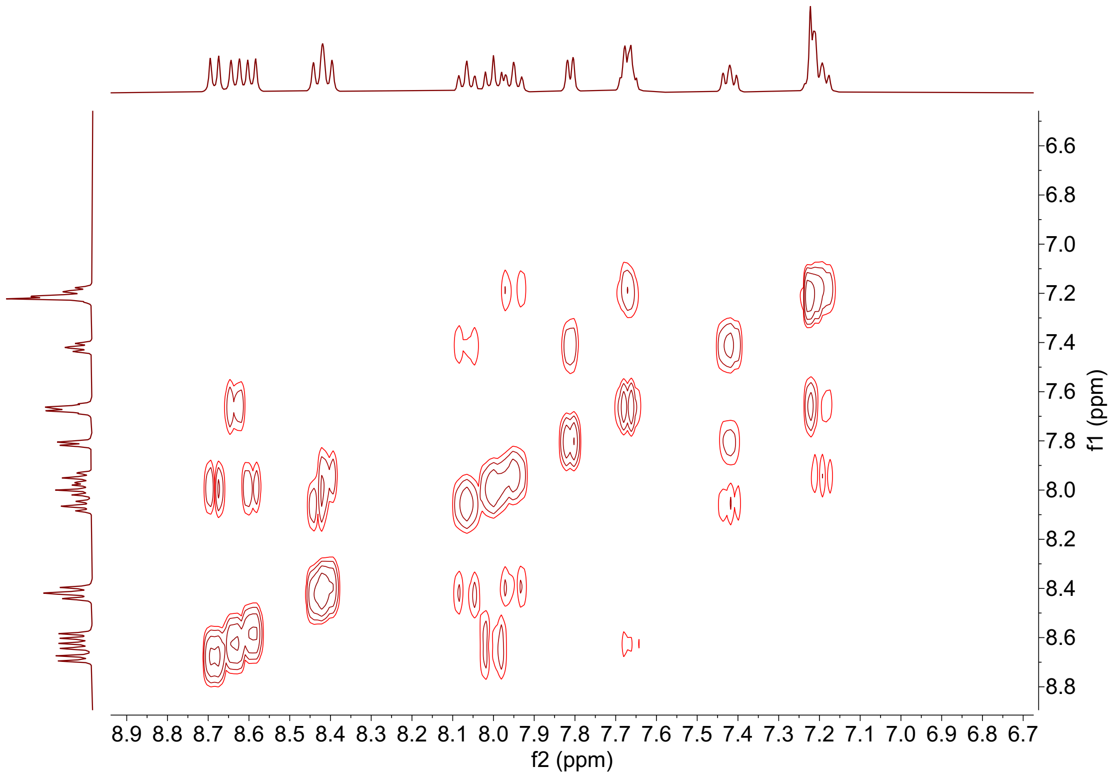

Figure S39. ^{1}H-^{1}H COSY NMR (22 °C, CD_3CN) of [Ru(bpy)$_2$(*p*-biphe)](PF_6)$_2$.

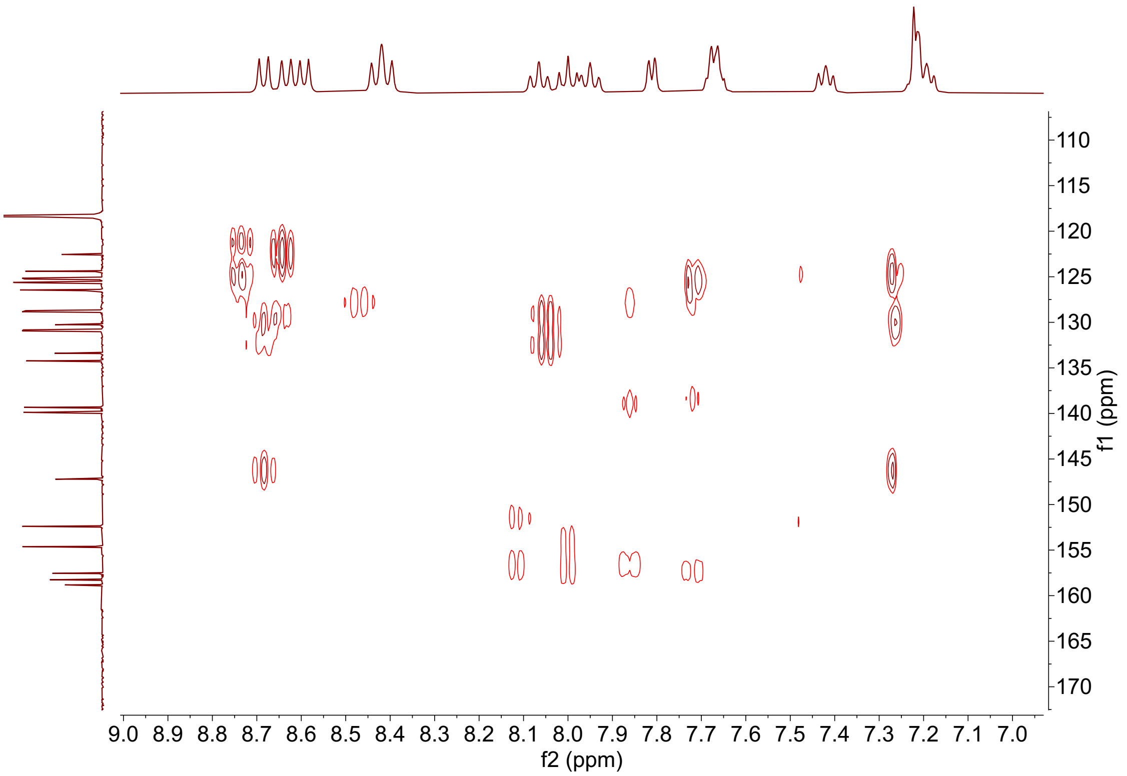

Figure S40. ^{1}H-^{13}C HMBC NMR (22 °C, CD_3CN) of [Ru(bpy)$_2$(*p*-biphe)](PF_6)$_2$.

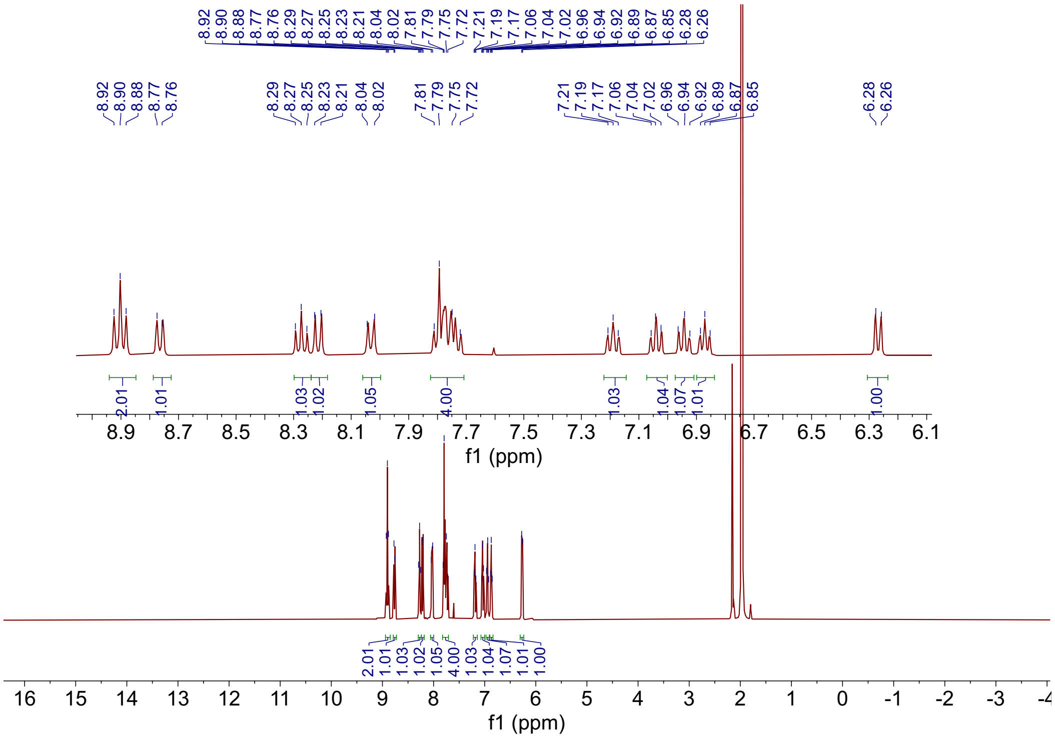

Figure S41. ^{1}H NMR (400 MHz, 22 °C, CD_3CN) of $[Ir(ppy)_2(p\text{-biphe})]PF_6$.

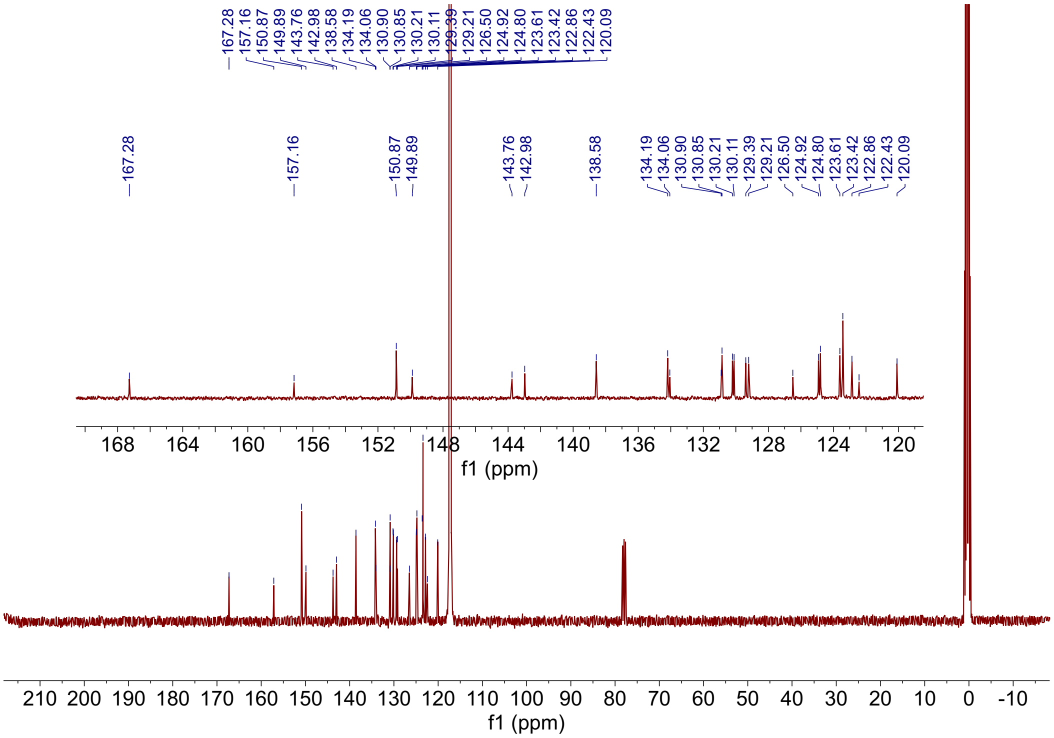

Figure S42. ^{13}C NMR (100 MHz, 22 °C, CD_3CN) of $[Ir(ppy)_2(p\text{-biphe})]PF_6$.

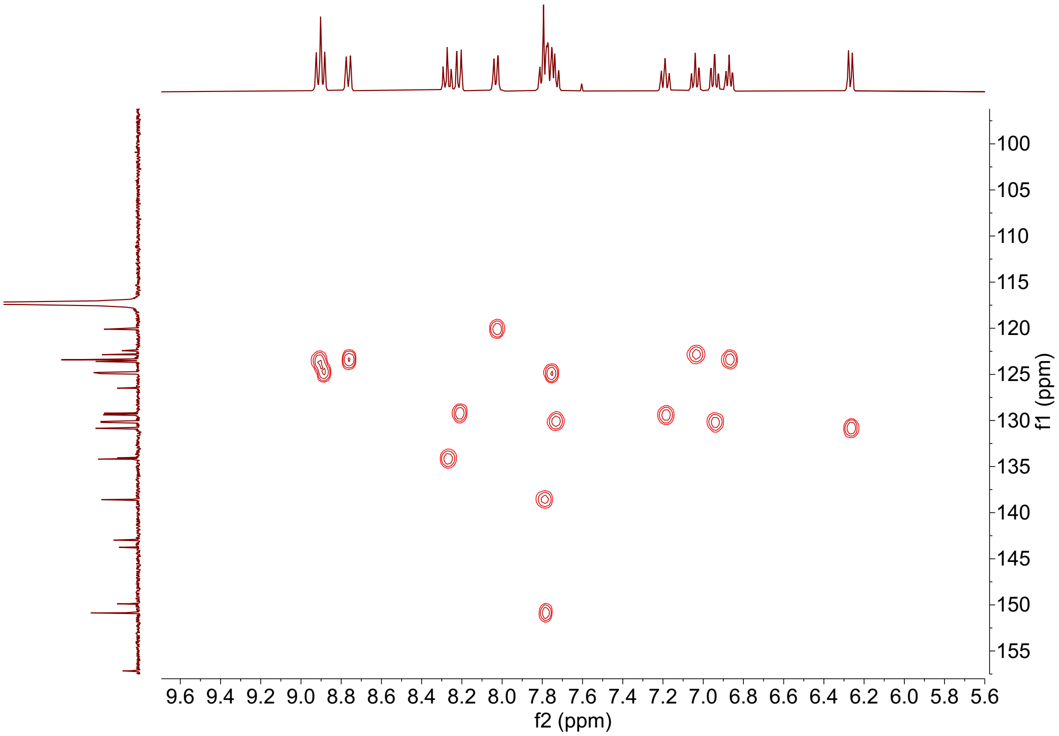

Figure S43. ^{1}H-^{13}C HSQC NMR (22 °C, CD_3CN) $[Ir(ppy)_2(p\text{-biphe})]PF_6$.

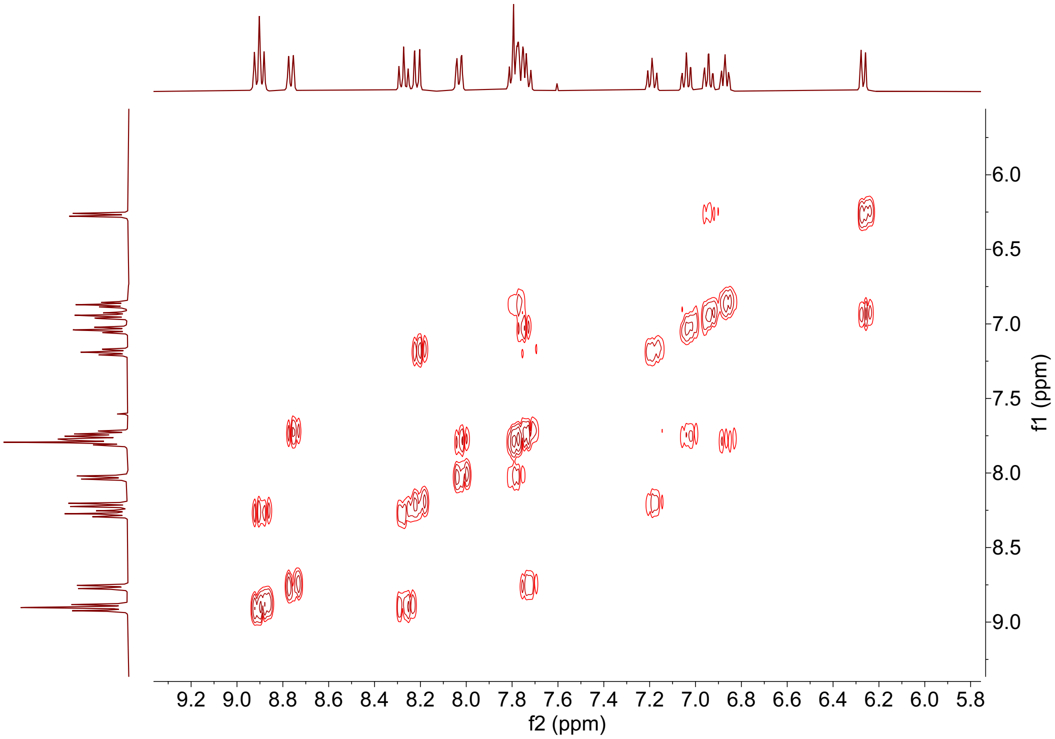

Figure S44. ^{1}H-^{1}H COSY NMR (22 °C, CD_3CN) of $[Ir(ppy)_2(p\text{-biphe})]PF_6$.

MASS SPECTRA

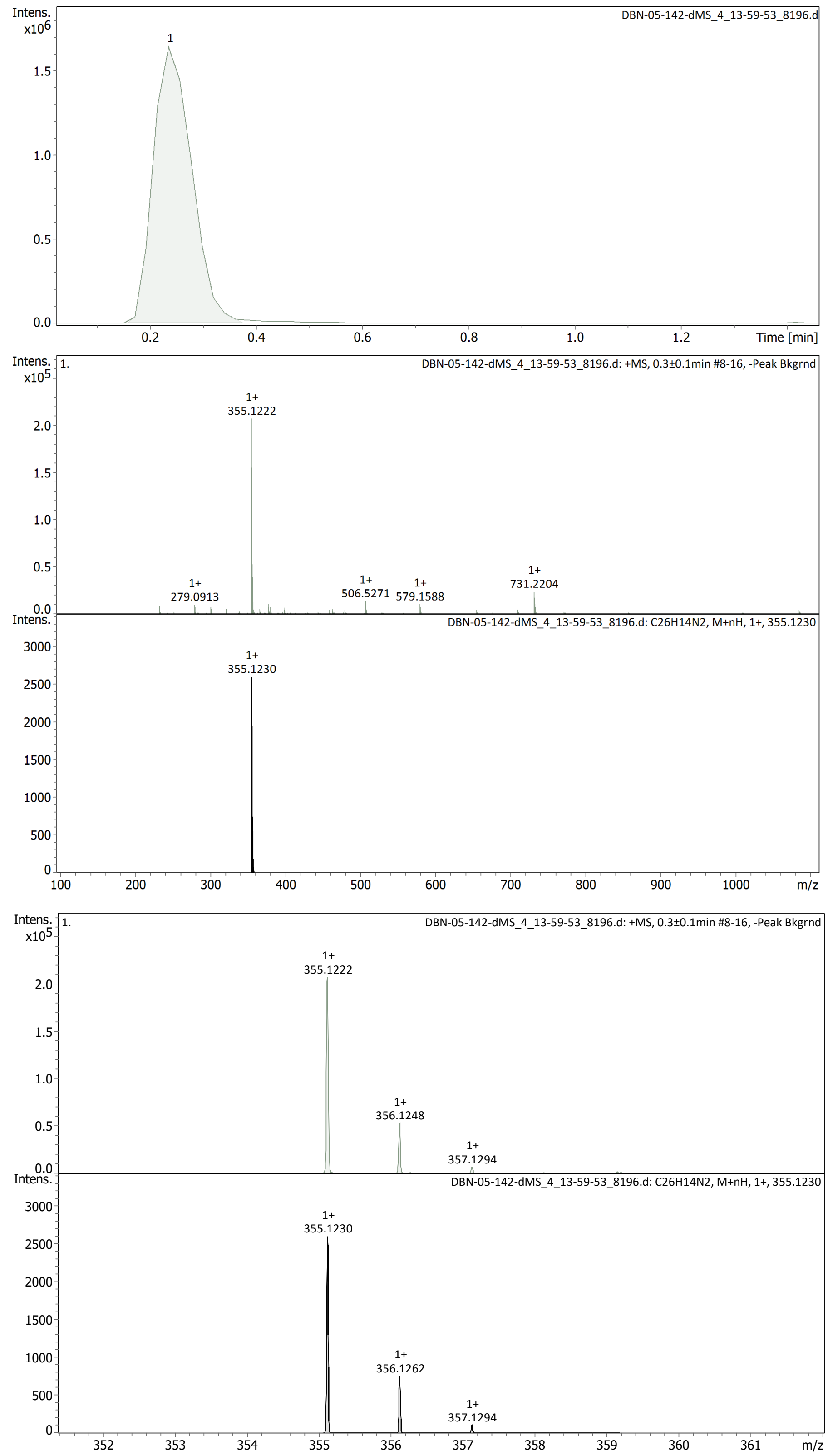

Figure S45: HR-MS (ESI-TOF/MS) of *p*-biphe.

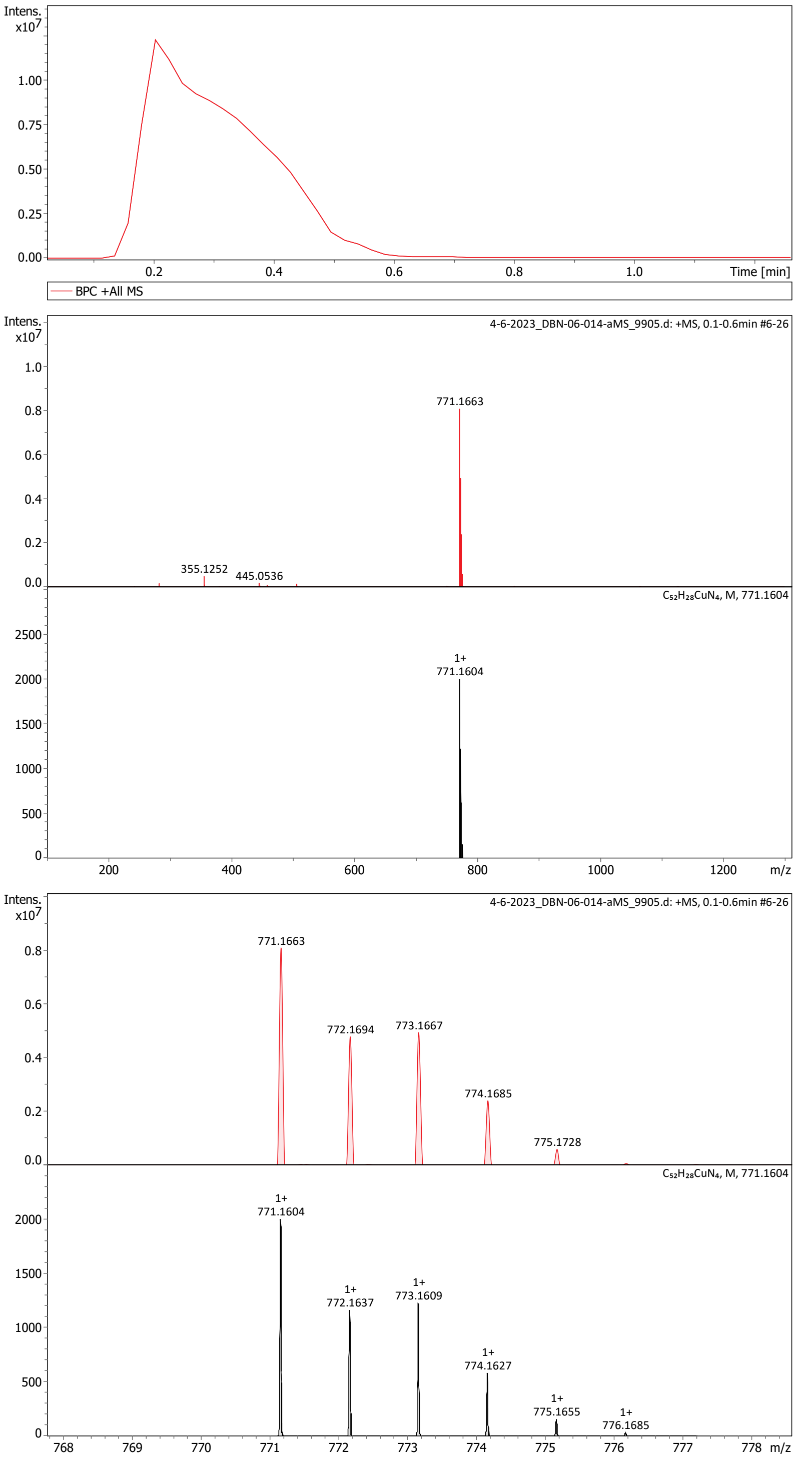

Figure S46. HR-MS (ESI-TOF/MS) of [Cu(p-biphe)$_2$]PF_6.

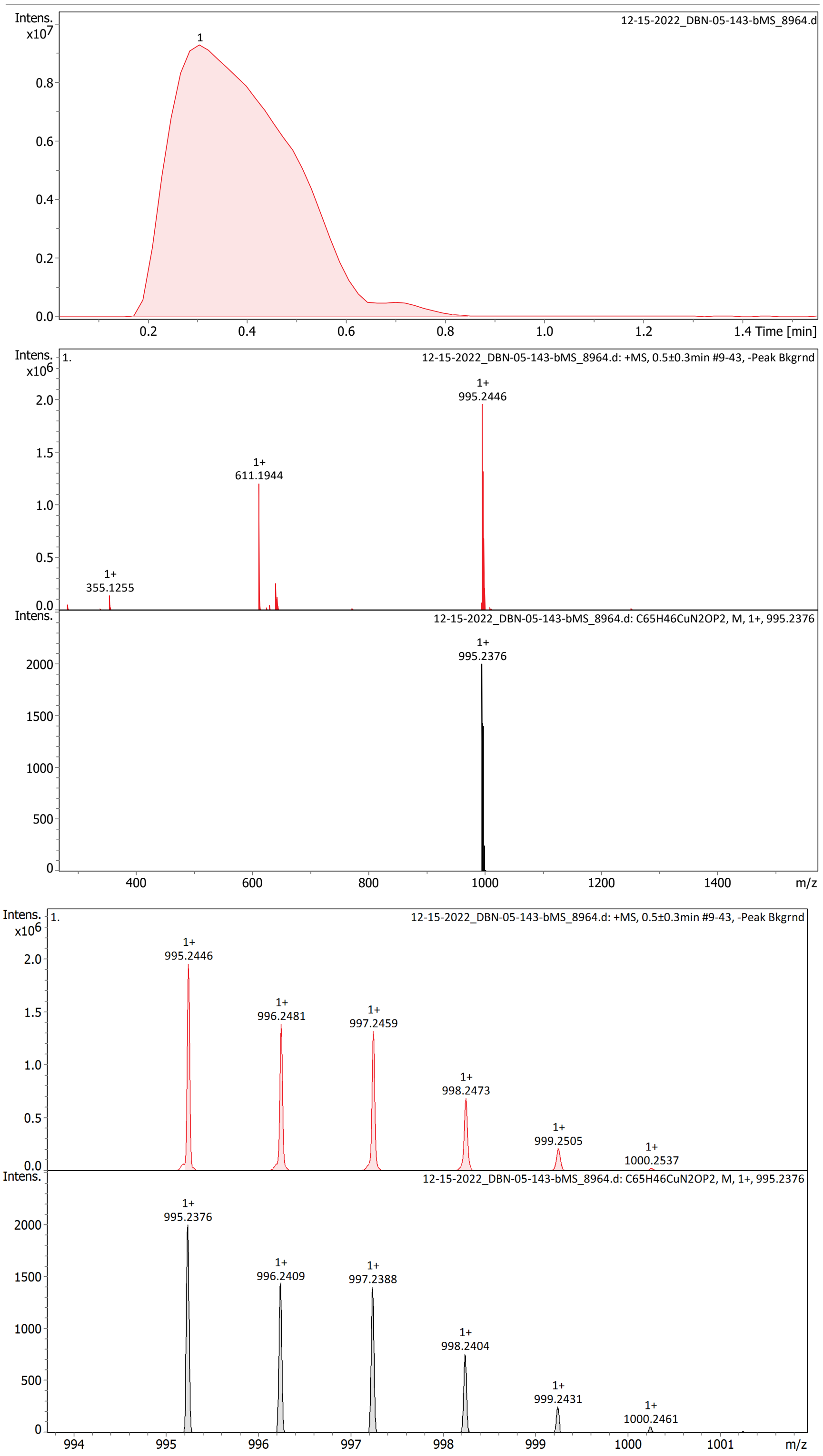

Figure S47. HR-MS (ESI-TOF/MS) of [(P^P)Cu(*p*-biphe)]PF_6.

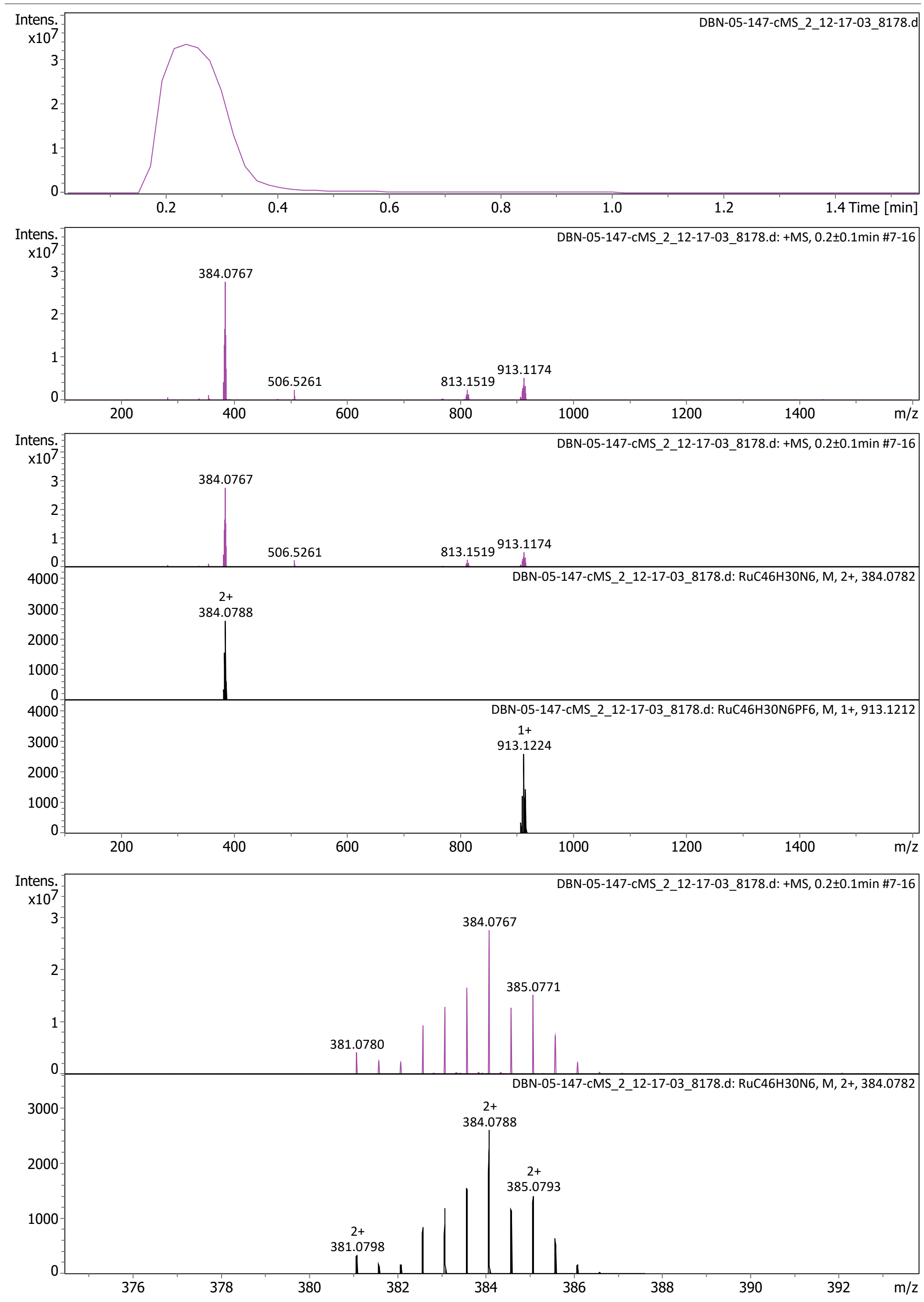

Figure S48. HR-MS (ESI-TOF/MS) of $[Ru(bpy)_2(\textit{p}\text{-biphe})](PF_6)_2$.

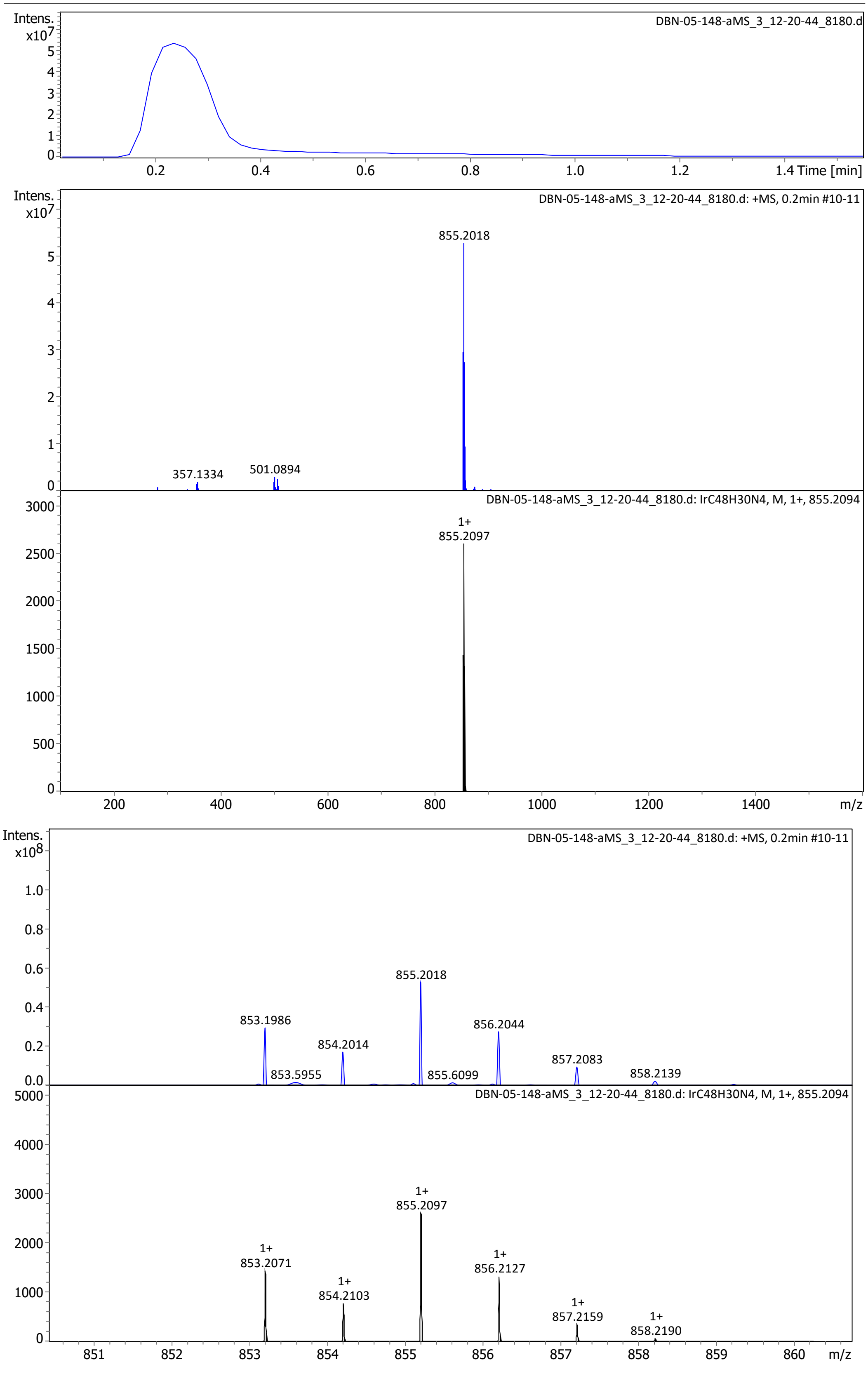

Figure S49. HR-MS (ESI-TOF/MS) of **[Ir(ppy)$_2$(pBiphe)]PF$_6$.**

Table S15. Comparison of experimentally and computationally[a] determined bond distances (Å) and angles (°) for $[Cu(p\text{-biphe})_2]^+$.

Bond/Å	$[Cu(p\text{-biphe})_2]^+$		
	XRD	DFT [a]	|Δd|
$Cu\text{-}N_1$	1.995	2.003	0.008
$Cu\text{-}N_2$	2.053	2.007	0.046
$Cu\text{-}N_3$	2.000	2.006	0.006
$Cu\text{-}N_4$	2.038	2.005	0.033
MAD [b]			0.023
**Angle/°**	**$[Cu(p\text{-biphe})_2]^+$**		
	XRD	DFT	|Δd|
$N_1\text{-}Cu\text{-}N_3$	136.9	128.8	8.1
$N_2\text{-}Cu\text{-}N_4$	108.2	129.1	20.9
$N_1\text{-}Cu\text{-}N_2$	81.2	81.7	0.5
$N_1\text{-}Cu\text{-}N_4$	125.0	120.9	4.1
$N_2\text{-}Cu\text{-}N_3$	126.6	121.3	5.3
$N_3\text{-}Cu\text{-}N_4$	81.1	81.7	0.6
MAD [b]			6.6
τ_δ [c]	0.63	0.72	

[a] SMD-O3LYP-D4/def2-SVP

[b] Mean absolute deviation

[c] τ_δ calculated as per reference [[8]]

Table S16. Comparison of experimentally and computationally[a] determined bond distances (Å) and angles (°) for [(P^P)Cu(*p*-biphe)]$^+$.

Bond/Å	**[(P^P)Cu(*p*-biphe)]$^+$**		
	XRD	DFT [a]	|Δd|
Cu-N_1	2.085	2.053	0.032
Cu-N_2	2.072	2.054	0.018
Cu-P_1	2.275	2.247	0.038
Cu-P_2	2.281	2.262	0.019
MAD [b]			0.027
Angle/°	**[(P^P)Cu(*p*-biphe)]$^+$**		
	XRD	DFT	|Δd|
N_1-Cu-P_1	115.1	128.2	13.1
N_2-Cu-P_2	109.0	101.4	7.6
N_1-Cu-N_2	78.4	80.0	1.6
P_1-Cu-P_2	118.5	120.5	2.0
N_1-Cu-P_2	115.7	96.7	19.0
N_2-Cu-P_1	113.0	121.2	8.2
MAD [b]			8.6
τ_δ [c]	0.87	0.74	

[a] SMD-O3LYP-D4/def2-SVP

[b] Mean absolute deviation

[c] τ_δ calculated as per reference [[8]]

Table S17. Comparison of experimentally and computationally[a] determined bond distances (Å) and angles (°) for **[Ru(bpy)$_2$(*p*-biphe)]$^{2+}$.**

Bond/Å	**[Ru(bpy)$_2$(*p*-biphe)]$^{2+}$**		
	XRD	DFT [a]	|Δd|
Ru-N$_1$	2.044	2.085	0.041
Ru-N$_2$	2.082	2.078	0.004
Ru-N$_3$	2.059	2.042	0.017
Ru-N$_4$	2.059	2.050	0.009
Ru-N$_5$	2.074	2.065	0.009
Ru-N$_6$	2.090	2.070	0.020
MAD [b]			0.017
**Angle/°**	**[Ru(bpy)$_2$(*p*-biphe)]$^{2+}$**		
	XRD	DFT	|Δd|
N$_1$-Ru-N$_5$	165.3	168.4	3.1
N$_2$-Ru-N$_4$	176.9	179.1	2.2
N$_3$-Ru-N$_6$	173.1	173.7	0.6
N$_1$-Ru-N$_2$	77.7	78.0	0.3
N$_1$-Ru-N4	102.1	102.8	0.7
N$_3$-Ru-N$_4$	78.3	79.1	0.8
MAD [b]			1.3

[a] SMD-PBE0-D3(BJ)/def2-SVP

[b] Mean absolute deviation

Table S18. Comparison of experimentally and computationally[a] determined bond distances (Å) and angles (°) for $[Ir(ppy)_2(p\text{-biphe})]^+$.

Bond/Å	$[Ir(ppy)_2(p\text{-biphe})]^+$		
	XRD	DFT [a]	|Δd|
$Ir\text{-}N_1$	2.180	2.217	0.037
$Ir\text{-}N_2$	2.185	2.210	0.025
$Ir\text{-}N_3$	2.038	2.046	0.008
$Ir\text{-}N_4$	2.067	2.064	0.003
$Ir\text{-}C_1$	2.012	2.002	0.010
$Ir\text{-}C_2$	2.021	2.008	0.013
MAD [b]			0.016
**Angle/°**	$[Ir(ppy)_2(p\text{-biphe})]^+$		
	XRD	DFT	|Δd|
$N_1\text{-}Ir\text{-}C_2$	171.0	169.5	1.5
$N_2\text{-}Ir\text{-}C_1$	177.3	178.8	1.6
$N_3\text{-}Ir\text{-}N_4$	175.2	174.1	1.1
$N_1\text{-}Ir\text{-}N_2$	75.0	74.3	0.7
$N_1\text{-}Ir\text{-}C_1$	104.5	104.8	0.3
$N_3\text{-}Ir\text{-}C_1$	80.1	80.6	0.5
MAD [b]			0.9

[a] SMD-PBE0-D3(BJ)/def2-SVP

[b] Mean absolute deviation

REFERENCES

[1] U. Mayer, V. Gutmann, W. Gerger, *Monatsh. Chem.* **1975**, *106*, 1235–1257.
[2] K. A. Veilleux, G. Schreckenbach, D. E. Herbert, *Mol. Syst. Des. Eng.* **2024**, *9*, 423–435.
[3] X. Xu, T. Xia, X.-L. Chen, X. Hao, T. Liang, H.-R. Li, H.-Y. Gong, *New J. Chem.* **2022**, *46*, 11835–11839.
[4] R. Ketkaew, Y. Tantirungrotechai, P. Harding, G. Chastanet, P. Guionneau, M. Marchivie, D. J. Harding, *Dalton Trans.* **2021**, *50*, 1086–1096.
[5] F. L. Hirshfeld, *Theoret. Chim. Acta* **1977**, *44*, 129–138.
[6] S. M. Draper, D. J. Gregg, E. R. Schofield, W. R. Browne, M. Duati, J. G. Vos, P. Passaniti, *J. Am. Chem. Soc.* **2004**, *126*, 8694–8701.
[7] A. Juris, V. Balzani, F. Barigelletti, S. Campagna, P. Belser, A. Von Zelewsky, *Coord. Chem. Rev.* **1988**, *84*, 85–277.
[8] M. H. Reineke, M. D. Sampson, A. L. Rheingold, C. P. Kubiak, *Inorg. Chem.* **2015**, *54*, 3211–3217.
